# Supplementary material for: Prognostic models for predicting in-hospital paediatric mortality in resource-limited countries: a systematic review
Source: BMJ Open. 2020 Oct 19;10(10):e035045. doi: 10.1136/bmjopen-2019-035045 (PMC7574949; doi:10.1136/bmjopen-2019-035045)
Supplement: Supplementary data [file bmjopen-2019-035045supp001.pdf]

Table 1: Systematic review framework as recommended by CHARMS checklist

Item	Criteria
Prognostic or diagnostic model	Prognostic model predicting in-hospital mortality.
Scope	Prognostic models to inform clinicians about the risk of deterioration or death.
Type of prediction models	Prognostic models with and/or without external validation.
Prediction target population	Children aged > 1 month to 15 years admitted in pediatric wards in developing countries
Outcome of interest	All-cause in-hospital mortality.
Prediction period	Any
Intended moment to apply the prediction tool	Prognostic model to be used in primary prevention to assess risk of deterioration and thus guide prevention/treatment.

KEY:

CHARMS= Checklist for critical Appraisal and data extraction for systematic Reviews of prediction Modelling Studies

Table 2: Search terms for prognostic models

Search ID	Sub-heading	Search Terms
S4	Children	paediatric* OR pediatric* OR (MH "Pediatrics+") OR child*
S3	Hospital based	(MH "Hospitals+") OR hospital*
S2	Low-income countries	(MH "Developing Countries+") OR (MH "Africa+") OR TI ("low income" OR "low and middle income"OR "LMIC" OR "LIC" OR "limited resource*" OR "poor resource*" OR "resource* poor" OR ("developing countries") OR ("developing nations") OR ("third world") OR "resource-constrained" OR ("global south")
S1	Predictive models	prognos* OR (MH "prognosis") OR (Predict* AND (Outcome* OR Risk* OR Model* OR Mortality OR Index OR Rule* OR decision* OR scor*)) OR "risk score" OR "scor* system" OR "logistic model*" "risk prediction" OR "risk calculation" OR "risk assessment" OR "c statistic" OR discrimination OR calibration OR AUC OR "area under the curve" OR "area under the receiver operator characteristic curve"

Table 3: List of domains and signaling questions used for assessment of risk of bias according to the PROBAST tool.

Domain	Signalling question
**Participants selection**	Were appropriate data sources used, e.g., cohort, RCT, or nested case–control study data?
	Were all inclusions and exclusions of participants appropriate?
**Predictors**	Were predictors defined and assessed in a similar way for all participants?
	Were predictor assessments made without knowledge of outcome data?
	Are all predictors available at the time the model is intended to be used?
**Outcome**	Was the outcome determined appropriately?
	Was a prespecified or standard outcome definition used?
	Were predictors excluded from the outcome definition?
	Was the outcome defined and determined in a similar way for all participants?
	Was the outcome determined without knowledge of predictor information?
	Was the time interval between predictor assessment and outcome determination appropriate?
**Analysis**	Were there a reasonable number of participants with the outcome?
	Were continuous and categorical predictors handled appropriately?
	Were all enrolled participants included in the analysis?
	Were participants with missing data handled appropriately?
	Was selection of predictors based on univariable analysis avoided?
	Were complexities in the data (e.g., censoring, competing risks, sampling of control participants) accounted for appropriately?
	Were relevant model performance measures evaluated appropriately?
	Were model overfitting, underfitting, and optimism in model performance accounted for?
	Do predictors and their assigned weights in the final model correspond to the results from the reported multivariable analysis?

KEY:

PROBAST= Prediction study Risk of Bias Assessment Tool
