# Supplementary material for: Prognostic models for predicting in-hospital paediatric mortality in resource-limited countries: a systematic review
Source: BMJ Open. 2020 Oct 19;10(10):e035045. doi: 10.1136/bmjopen-2019-035045 (PMC7574949; doi:10.1136/bmjopen-2019-035045)
Supplement: Supplementary data [file bmjopen-2019-035045supp002.pdf]

Study	***Model name***	***Country***	***Source of data***	***Study year***	***Inclusion criteria***	***Age***	***Outcome***
Berkley 2003	PEDIA Immediate death	Kenya	Prospective cohort	1998-2000	Aged over 90 days	3 months-13 years	Mortality
Berkley 2003	PEDIA Early death	Kenya	Prospective cohort	1998-2000	Aged over 90 days	3 months-13 years	Mortality
Berkley 2003	PEDIA Late death	Kenya	Prospective cohort	1998-2000	Aged over 90 days	3 months-13 years	Mortality
Bitwe 2006	Goma 1 Model	Democratic Republic of Congo	Prospective cohort	2003-2004	<12 months	Median: 12.8 months	Mortality
Draimax 1996		Congo	Prospective cohort	1986-1988	Malnutrition	Median: 27 months	Mortality
Kumar 2003	SICK score	India	Prospective cohort	1998-1999	Paediatric patients	No Information	Mortality
Geoge 2015	PET Score	Kenya, Uganda, Tanzania	RCT	2009-2011	Malaria	Median: 24 (IQR=13-38)	Mortality
Emukule 2014	mRISC	Kenya	Surveillance	2009-2012	Under 5 years hospitalized with severe acute respiratory illness	<59 months	Mortality
Reed 2012	RISC-HIV positive	South Africa	RCT	1998-2001	LRTI hospitalizations under 24 months with HIV infection	<24 months	Mortality
Reed 2012	RISC-HIV Negative	South Africa	RCT	1998-2001	LRTI hospitalizations under 24 months without HIV infection	<24 months	Mortality
Hooli 2016	RISC-Malawi	Malawi	Retrospective observational study	2011-2014	0-59 months hospitalized with pneumonia	<59 months	Mortality
Gallagher 2019	PERCH Score	Kenya, Zambia, South Africa, Mali, Gambia, Bangladesh, Thailand	Case-control study	2011–2014	1–59 months HIV negative hospitalized with severe or very severe pneumonia	Median: 9(4-19) months	In-hospital mortality and 7-days post-

							discharge mortality
Helbok 2009	LOD score	Gambia,Malawi, Kenya,Ghana,Gabon	Prospective cohort	2000-2005	Hospitalized children with severe malaria	28(0-180)	Mortality
Erdman 2011 (Logistic regression)	Biomarker score	Uganda	Retrospective nested case-control study	2007-2009	6 months - 12 years	6 months - 12 years	Mortality
Erdman 2011 (Classification tree)		Uganda	Retrospective nested case-control study	2007-2009	6 months - 12 years	6 months - 12 years	Mortality
Lowlaavar 2016	Model 1	Uganda	Prospective observational study	2012-2013	6–60 months admitted with infectious illness	Median 18.2 (IQR 11.9–33.1) months	Mortality
Lowlaavar 2016	Model 2	Uganda	Prospective observational study	2012-2013	6–60 months admitted with infectious illness	Median 18.2 (IQR 11.9–33.1) months	Mortality
Lowlaavar 2016	Model 3	Uganda	Prospective observational study	2012-2013	6–60 months admitted with infectious illness	Median 18.2 (IQR 11.9–33.1) months	Mortality
Mpimbaza 2015		Uganda	Surveillance	2010-2013	General paediatrics	18 months (IQR 9–36)	Mortality
Olson 2013	ITAT score	Malawi	Nested case–control	2010-2011	age <15 years on the acute care and malnutrition wards	≤15 years	Mortality
Rosman 2019	PEWS-RL	Rwanda	Case-control study	2016-2017	0-18 years patients admitted to pediatric department	0-18 years	Mortality

Study	***Sample size***	***Number of outcome events***	***Missing data handling***	***Number of participants with missing data reported?***	***Regression method***	***Were model assumptions verified***
Berkley 2003	429	60	No Information	No Information	Spiegelhalter/Knill-Jones method	Yes

Berkley 2003	439	193	No Information	No Information	Spiegelhalter/Knill-Jones method	Yes
Berkley 2003	436	183	No Information	No Information	Spiegelhalter/Knill-Jones method	Yes
Bitwe 2006	414	66	No Information	No Information	Logistic regression	Yes
Draimax 1996	1129	196	No Information	No Information	Logistic regression	No Information
Kumar 2003	1099	44	No Information	No Information	Logistic regression	No Information
Geoge 2015	3170	315	Complete case analyses	Yes	Cox proportional hazards regression	No Information
Emukule 2014	3581	218	Complete case analyses	No Information	Logistic regression	Yes
Reed 2012	1502	265	Complete case analyses	No Information	Logistic regression	No Information
Reed 2012	2646	33	Complete case analyses	No Information	Logistic regression	No Information
Hooli 2016	14665	464	Multiple imputation	Yes	Logistic regression	Yes
Gallagher 2019	1802	120	Complete case analyses	No Information	Logistic regression	No Information
Helbok 2009	23980	1004	Complete case analyses	Yes	Logistic regression	No Information
Erdman 2011 (Logistic regression)	103	23	No missing values	Yes	Logistic regression	Yes
Erdman 2011 (Classification tree)	103	23	No missing values	Yes	Classification tree	No Information
Lowlaavar 2016	1307	65	Multiple imputation	No Information	Logistic regression	No Information
Lowlaavar 2016	1307	65	Multiple imputation	No Information	Logistic regression	No Information
Lowlaavar 2016	1307	65	Multiple imputation	No Information	Logistic regression	No Information

Mpimbaza 2015	50249	1742	Complete case analyses	Yes	Logistic regression	No Information
Olson 2013	1606	54	Single imputation	Yes	Logistic regression	Yes
Rosman 2019	168	57	Complete case analyses	No Information	Logistic regression	No Information

Study	***Predictor selection***	***Was a shrinkage method used***	***Calibration method***	***Discrimination***
Berkley 2003	Univariate	No Information	No Information	0.93(0.92-0.94)
Berkley 2003	Univariate	No Information	No Information	0.82(0.80-0.83)
Berkley 2003	Univariate	No Information	No Information	0.82(0.81-0.84)
Bitwe 2006	Univariate & Stepwise	No Information	Yes	0.83 (0.78-0.88)
Draimax 1996	A priori	No Information	No Information	0.85(No information)
Kumar 2003	Univariate(but included all variables in final model)	No Information	No Information	0.89
Geoge 2015	A priori	No Information	Hosmer-Lemeshow test, P=0.30	0.82(0.77–0.87)
Emukule 2014	Univariate	Yes	Calibration plot	0.85
Reed 2012	Univariate	No Information	Hosmer-Lemeshow test, P=0.95	0.78
Reed 2012	Univariate	No Information	Hosmer-Lemeshow test, P=0.87	0.92
Hooli 2016	A priori	No Information	Risk predictiveness curve	0.79 (95% CI: 0.76±0.82)
Gallagher 2019	Univariate	No Information	Calibration plot	0.84(No Information)
Helbok 2009	Forward & backward Stepwise	No Information	No Information	80 (79–82)

Erdman 2011 (Logistic regression)	Univariate	No Information	Hosmer-Lemeshow test and calibration slope analysis	0.96(0.90–0.99)
Erdman 2011 (Classification tree)	No Information	No Information	No Information	No Information
Lowlaavar 2016	Univariate & Stepwise	No Information	No Information	0.85 (0.80–0.89)
Lowlaavar 2016	Univariate & Stepwise	No Information	No Information	0.84 (0.79–0.89)
Lowlaavar 2016	Univariate & Stepwise	No Information	No Information	0.82 (0.72–0.91)
Mpimbaza 2015	Backward	No Information	General paediatrics	0.76(No information)
Olson 2013	Univariate	No Information	No Information	0.76(No information)
Rosman 2019	Univariate	No Information	No Information	0.96 (95% CI 0.93–0.99).

Study	***Classification measures reported***	***Method used for internal validation***	***External validation***	***Was a simplified model presented***	***Were coefficients (including intercept) of the regression model presented***
Berkley 2003	No Information	Separate dataset	Yes	Yes	NA
Berkley 2003	No Information	Separate dataset	Yes	Yes	NA
Berkley 2003	No Information	Separate dataset	Yes	Yes	NA
Bitwe 2006	No Information	No Information	No	Yes	No
Draimax 1996	Positive predictive values 40% and negative predictive value of 97.9%	Separate dataset	No	Yes	No
Kumar 2003	Maximum discrimination was observed at a score of 2.5 with a sensitivity of 84.1% and of specificity 82.2%	No Information	Yes	Yes	No
Geoge 2015	No Information	Separate dataset	No	Yes	No
Emukule 2014	A score of >6 has a sensitivity of 1.8% and specificity 99.9%	Bootstrapping	Yes	Yes	No
Reed 2012	Score of 7 has a sensitivity of 4% and specificity of 99%	Bootstrapping	No	Yes	No

Reed 2012	Score of 6 has a sensitivity: 16% Specificity: 99%	Bootstrapping	Yes	Yes	No
Hooli 2016	a score of 8 has sensitivity of 57% and specificity of 88%	No Information	No	Yes	Yes
Gallagher 2019	positive predictive value 23.6%, positive predictive value 95.8%	Bootstrapping & separate dataset	No	Yes	No
Helbok 2009	LODS >=1, sensitivity was 85% and specificity was 63%	No Information	Yes	Yes	No
Erdman 2011 (Logistic regression)	sensitivity of 95.7% (95% CI: 78.1–99.9) and specificity of 88.8% (79.7–94.7) predicting death	Boostrappling	No	Yes	No
Erdman 2011 (Classification tree)	100% sensitivity and 92.5% specificity for predicting outcome	10-fold cross validation	No	No	No
Lowlaavar 2016	Sensitive: 0.83 (0.74–0.92), Specificity: 0.76 (0.73–0.78)	No Information	No	No	Yes
Lowlaavar 2016	Sensitive: 0.80 (0.70–0.90), Specificity: 0.76 (0.74–0.79)	No Information	No	No	Yes
Lowlaavar 2016	Sensitive: 0.82 (0.72–0.91), Specificity: 0.71 (0.68–0.73)	No Information	No	No	Yes
Mpimbaza 2015	No Information	Separate dataset	No	Yes	No
Olson 2013	sensitivity: 0.44, specificity: 0.86,PPV: 0.18, NPV: 0.96 for a cut-off of 4	No Information	No	Yes	No
Rosman 2019	PEWS-RL of >=3, sensitivity was 96.2%, and specificity was 87.3%	No Information	No	Yes	No

Study	***Number of predictors in final model***	***Predictors in the final model***	***Are there laboratory-based predictors***	***Handling of continuous predictors***	***Events per variable***
Berkley 2003	10	Severe anaemia, Jaundice, Subcostal indrawing, Deep breathing, prostrated with seizures, prostrated without	No	NA	6

		seizures, Impaired consciousness with seizures, Impaired consciousness without seizures, Axillary temperature <36 °C, Axillary temperature >39 °C			
Berkley 2003	8	Jaundice, Subcostal indrawing, Prostrated with seizures, Prostrated without seizures, Impaired consciousness with seizures, Impaired consciousness without seizures,Wasting, Kwashiorkor	No	NA	24.125
Berkley 2003	9	History >7 days, Prostrated with seizures, Prostrated without seizures, Impaired consciousness with seizures, Impaired consciousness without seizures, Axillary temperature <36 °C, Axillary temperature >39 °C, Wasting, Kwashiorkor	No	Dichotomized History	20.33333
Bitwe 2006	8	Age(<12, >=12months), Brachial Perimeter(<=115mm, >115mm), State of consciousness(Unconscious, Aware), Infectious diagnosis(Acute respiratory infection, Malaria, Gastroenteritis, Septicemia / bacteremia, Other infections)	No	Dichotomized Brachial perimeter & Age	8.25

Draimax 1996	4	MUAC, edema, Serum albumin, Transthyretin	Yes	MUAC	49
Kumar 2003	9	Temperature(Normal, Abnormal), Heart rate(Normal, Abnormal), Respiratory rate(Normal, Abnormal), Systolic blood pressure(Normal, Abnormal), Capillary refill time(Normal, Abnormal), Consciousness(Normal, Abnormal), Age(≥60, ≥12 to <60, ≥1to<12, <1)	No	Dichotomized most variables	4.888889
Geoge 2015	11	Temperature(≤37, >37), Heart rate(<80 bpm, ≥80 to <105 bpm, ≥220 bpm), Capillary refill time(≥2sec, <2sec), Conscious level(prostrate, coma), Respiratory distress, Lung crepitations, Severe pallor, Weak pulse, Weight(<6 kg, 6–8 kg), Deep breathing	No	multivariable fractional polynomials	28.63636
Emukule 2014	9	Lab confirmed malaria, Weight for age(Low, Very Low), Dehydration, Unconscious, Unable to drink/breastfeed, Night sweats, Chest wall in-drawing, Interaction between malaria and chest wall in-drawing, A.V.P.U scale - Not alert	Yes	Categorized weight for age	24.22222

Reed 2012	7	Oxygen saturation <90%, Chest indrawing, Wheezing, Refusing feeds, HIV classification(Severe, Mild or moderate), IMCI age group(<2 months, 3–12 months)	No		37.85714
Reed 2012	5	Oxygen saturation <90%, Chest indrawing, Wheezing, Refusing feeds, Weight for age(Low (<= -2 z-score), Very Low (<= -3 z-score))	No	Categorized weight for age	6.6
Hooli 2016	5	Oxygen saturation(moderate, severe), MUAC(moderate, severe), Gender, Wheeze, Consciousness	No	Categorized MUAC and Oxygen saturation	92.8
Gallagher 2019	12	Age(1-11, 12-59), sex, Unresponsiveness and/or deep breathing(Deep breathing, but alert, Unresponsive but no deep breathing, Unresponsive and deep breathing), cough, grunting, hypoxemia, length of illness(0–2, 3–5, >5), Weight-for-height z-score(Very low (< –3), Low (≥ –3 to < –2), Normal-high (≥ –2))	No	Categorized most variables	10
Helbok 2009	8	Convulsion, vomiting, deep breathing, intercostal recession, Coma,	Yes	NA	125.5

		Prostration,hyperparastemia, severe anemia			
Erdman 2011 (Logistic regression)	8	angiopoietin-2, soluble ICAM-1, soluble Flt-1, procalcitonin, IP-10, soluble TREM-1, age, parasitemia	Yes	NA	2.875
Erdman 2011 (Classification tree)	3	IP-10, Ang-2, sICAM-1	Yes	NA	7.666667
Lowlaavar 2016	3	Abnormal BCS, Positive HIV diagnosis, Weight-age z-score	Yes	Treated as continuous	21.66667
Lowlaavar 2016	3	Abnormal BCS, HIV diagnosis, MUAC	Yes	Treated as continuous	21.66667
Lowlaavar 2016	2	Abnormal BCS, MUAC	No	Treated as continuous	32.5
Mpimbaza 2015	13	Age, fever, difficulty breathing, altered consciousness, unable to drink or breastfeed, convulsions, temperature, unconsciousness, pallor, jaundice, deep breathing, meningeal signs, unable to sit up	No	NA	134
Olson 2013	4	Oxygen saturation, Temperature, Heart rate, Respiratory rate	No	Used splines	13.5
Rosman 2019	6	PEWS-RL score(0 to 6)	No	NA	9.5
