# Supplementary material for: Prognostic models for predicting in-hospital paediatric mortality in resource-limited countries: a systematic review
Source: BMJ Open. 2020 Oct 19;10(10):e035045. doi: 10.1136/bmjopen-2019-035045 (PMC7574949; doi:10.1136/bmjopen-2019-035045)
Supplement: Supplementary data [file bmjopen-2019-035045supp003.pdf]

Study	Participant Domain		
	Were appropriate data sources used, e.g., cohort, RCT, or nested case–control study data?	*Were all inclusions and exclusions of participants appropriate?*	*Risk of Bias in participants*
Berkley 2003 (PEDIA -Immediate)	Yes	Yes	Low
Berkley 2003(PEDIA -Early)	Yes	Yes	Low
Berkley 2003(PEDIA -Late)	Yes	Yes	Low
Bitwe 2006 (Goma score)	Yes	Yes	Low
Draimax 1996	Yes	Yes	Low
Kumar 2003(SICK score)	Yes	Yes	Low
Geoge 2015 (PET score)	Yes	Yes	Low
Emukule 2014 (mRISC score)	No	Yes	High
Reed 2012 (RISC HIV+)	Yes	Yes	Low
Reed 2012 (RISC HIV-)	Yes	Yes	Low
Hooli 2016(RISC-Malawi)	No	Yes	High
Gallagher 2019(PERCH Score)	Yes	Yes	Low
Helbok 2009(LOD score)	Yes	Yes	Low
Erdman 2011(logistic regression)	Yes	Yes	Low
Erdman 2011(CRT)	Yes	Yes	Low
Lowlaavar 2016 (Model 1)	Yes	Yes	Low
Lowlaavar 2016 (Model 2)	Yes	Yes	Low
Lowlaavar 2016 (Model 3)	Yes	Yes	Low
Mpimbaza 2015	No	Yes	High
Olson 2013(ITAT score)	Yes	Yes	Low
Rosman 2019(PEWS-RL score)	No	Yes	High

Study	Predictor Domain			
	Were predictors defined and assessed in a similar way for all participants	*Were predictor assessments made without knowledge of outcome data?*	*Are all predictors available at the time the model is intended to be used?*	*Risk of Bias in predictors*
Berkley 2003 (PEDIA -Immediate)	Yes	Yes	Yes	Low
Berkley 2003(PEDIA -Early)	Yes	Yes	Yes	Low
Berkley 2003(PEDIA -Late)	Yes	Yes	Yes	Low
Bitwe 2006 (Goma score)	Yes	Yes	Yes	Low
Draimax 1996	Yes	Yes	No	High
Kumar 2003(SICK score)	Yes	Yes	Yes	Low
Geoge 2015 (PET score)	Yes	Yes	Yes	Low
Emukule 2014 (mRISC score)	Yes	Yes	No	High
Reed 2012 (RISC HIV+)	Yes	Yes	Yes	Low
Reed 2012 (RISC HIV-)	Yes	Yes	Yes	Low
Hooli 2016(RISC-Malawi)	Yes	Yes	Yes	Low
Gallagher 2019(PERCH Score)	Yes	Yes	Yes	Low
Helbok 2009(LOD score)	Yes	Yes	Yes	Low
Erdman 2011(logistic regression)	Yes	Yes	Yes	Low
Erdman 2011(CRT)	Yes	Yes	Yes	Low
Lowlaavar 2016 (Model 1)	Yes	Yes	No	High
Lowlaavar 2016 (Model 2)	Yes	Yes	No	High
Lowlaavar 2016 (Model 3)	Yes	Yes	Yes	Low
Mpimbaza 2015	Yes	Yes	Yes	Low
Olson 2013(ITAT score)	Yes	Yes	Yes	Low
Rosman 2019(PEWS-RL score)	Yes	Yes	Yes	Low

**Study**	**Outcome Domain**						
	Was the outcome determined appropriately?	*Was a prespecified or standard outcome definition used?*	*Were predictors excluded from the outcome definition?*	*Was the outcome defined and determined in a similar way for all participants?*	*Was the outcome determined without knowledge of predictor information?*	*Was the time interval between predictor assessment and outcome determination appropriate?*	*Risk of Bias in outcome*
Berkley 2003 (PEDIA -Immediate)	Yes	Yes	Yes	Yes	Yes	Yes	Low
Berkley 2003(PEDIA -Early)	Yes	Yes	Yes	Yes	Yes	Yes	Low
Berkley 2003(PEDIA -Late)	Yes	Yes	Yes	Yes	Yes	Yes	Low
Bitwe 2006 (Goma score)	Yes	Yes	Yes	Yes	Yes	Yes	Low
Draimax 1996	Yes	Yes	Yes	Yes	Yes	Yes	Low
Kumar 2003(SICK score)	Yes	Yes	Yes	Yes	Yes	Yes	Low
Geoge 2015 (PET score)	Yes	Yes	Yes	Yes	Yes	Yes	Low
Emukule 2014 (mRISC score)	Yes	Yes	Yes	Yes	Yes	Yes	Low
Reed 2012 (RISC HIV+)	Yes	Yes	Yes	Yes	Yes	Yes	Low
Reed 2012 (RISC HIV-)	Yes	Yes	Yes	Yes	Yes	Yes	Low
Hooli 2016(RISC-Malawi)	Yes	Yes	Yes	Yes	Yes	Yes	Low
Gallagher 2019(PERCH Score)	Yes	Yes	Yes	Yes	Yes	Yes	Low
Helbok 2009(LOD score)	Yes	Yes	Yes	Yes	Yes	Yes	Low
Erdman 2011(logistic regression)	Yes	Yes	Yes	Yes	Yes	Yes	Low
Erdman 2011(CRT)	Yes	Yes	Yes	Yes	Yes	Yes	Low
Lowlaavar 2016 (Model 1)	Yes	Yes	Yes	Yes	Yes	Yes	Low
Lowlaavar 2016 (Model 2)	Yes	Yes	Yes	Yes	Yes	Yes	Low
Lowlaavar 2016 (Model 3)	Yes	Yes	Yes	Yes	Yes	Yes	Low
Mpimbaza 2015	Yes	Yes	Yes	Yes	Yes	Yes	Low
Olson 2013(ITAT score)	Yes	Yes	Yes	Yes	Yes	Yes	Low
Rosman 2019(PEWS-RL score)	Yes	Yes	Yes	Yes	Yes	Yes	Low

**Study**	**Analyses Domain**				
	Were there a reasonable number of participants with the outcome?	*Were continuous and categorical predictors handled appropriately?*	*Were all enrolled participants included in the analysis?*	*Were participants with missing data handled appropriately?*	*Was selection of predictors based on univariable analysis avoided*
Berkley 2003 (PEDIA -Immediate)	No	Yes	Yes	Probably No	No
Berkley 2003(PEDIA -Early)	Yes	Yes	Yes	Probably No	No
Berkley 2003(PEDIA -Late)	Yes	No	Yes	Probably No	No
Bitwe 2006 (Goma score)	No	No	Yes	No Information	No
Draimax 1996	Yes	No	Yes	No Information	Yes
Kumar 2003(SICK score)	No	No	Yes	No Information	No
Geoge 2015 (PET score)	Yes	Yes	Yes	No	Yes
Emukule 2014 (mRISC score)	Yes	No	Yes	No	No
Reed 2012 (RISC HIV+)	Yes	Yes	Yes	No Information	No
Reed 2012 (RISC HIV-)	No	No	Yes	No Information	No
Hooli 2016(RISC-Malawi)	Yes	No	Yes	Yes	Yes
Gallagher 2019(PERCH Score)	Yes	No	Yes	No	No
Helbok 2009(LOD score)	Yes	Yes	Yes	No	No
Erdman 2011(logistic regression)	No	Yes	Yes	Yes	No
Erdman 2011(CRT)	No	Yes	Yes	Yes	No Information
Lowlaavar 2016 (Model 1)	Yes	Yes	Yes	Yes	No
Lowlaavar 2016 (Model 2)	Yes	Yes	Yes	Yes	No
Lowlaavar 2016 (Model 3)	Yes	Yes	Yes	Yes	No
Mpimbaza 2015	Yes	Yes	Yes	No	No
Olson 2013(ITAT score)	Yes	Yes	Yes	Probably No	No
Rosman 2019(PEWS-RL score)	No	Yes	Yes	No	No

**Study**	**Analyses Domain**				
	Were complexities in the data (e.g., censoring, competing risks, sampling of control participants) accounted for appropriately?	*Were relevant model performance measures evaluated appropriately?*	*Were model overfitting, underfitting, and optimism in model performance accounted for?*	*Do predictors and their assigned weights in the final model correspond to the results from the reported multivariable analysis?*	*Risk of Bias in analysis*
Berkley 2003 (PEDIA -Immediate)	NA	Probably No	No Information	Probably Yes	Unclear
Berkley 2003(PEDIA -Early)	NA	Probably No	No Information	Probably Yes	Unclear
Berkley 2003(PEDIA -Late)	NA	Probably No	No Information	Probably Yes	Unclear
Bitwe 2006 (Goma score)	NA	Yes	No Information	Yes	Unclear
Draimax 1996	NA	No	No Information	Probably No	Unclear
Kumar 2003(SICK score)	NA	No	No Information	Yes	Unclear
Geoge 2015 (PET score)	No	No	No Information	Yes	Unclear
Emukule 2014 (mRISC score)	NA	Yes	Yes	Yes	High
Reed 2012 (RISC HIV+)	NA	Yes	Yes	Yes	Unclear
Reed 2012 (RISC HIV-)	NA	Yes	Yes	Yes	Unclear
Hooli 2016(RISC-Malawi)	NA	Yes	No Information	Yes	Unclear
Gallagher 2019(PERCH Score)	NA	Yes	Yes	Yes	High
Helbok 2009(LOD score)	NA	Probably No	No Information	Probably No	Unclear
Erdman 2011(logistic regression)	NA	No	Yes	No Information	Unclear
Erdman 2011(CRT)	NA	No Information	Yes	No Information	Unclear
Lowlaavar 2016 (Model 1)	NA	No	No Information	Yes	Unclear
Lowlaavar 2016 (Model 2)	NA	No	No Information	Yes	Unclear
Lowlaavar 2016 (Model 3)	NA	No	No Information	Yes	Unclear
Mpimbaza 2015	NA	No Information	No Information	Yes	Unclear
Olson 2013(ITAT score)	NA	No	No Information	No	Unclear
Rosman 2019(PEWS-RL score)	NA	No	No Information	No	Unclear
