# Supplementary material for: Prognostic models for predicting in-hospital paediatric mortality in resource-limited countries: a systematic review
Source: BMJ Open. 2020 Oct 19;10(10):e035045. doi: 10.1136/bmjopen-2019-035045 (PMC7574949; doi:10.1136/bmjopen-2019-035045)
Supplement: Author's manuscript [file bmjopen-2019-035045.draft_revisions.pdf]

BMJ Open is committed to open peer review. As part of this commitment we make the peer review history of every article we publish publicly available.

When an article is published we post the peer reviewers' comments and the authors' responses online. We also post the versions of the paper that were used during peer review. These are the versions that the peer review comments apply to.

The versions of the paper that follow are the versions that were submitted during the peer review process. They are not the versions of record or the final published versions. They should not be cited or distributed as the published version of this manuscript.

BMJ Open is an open access journal and the full, final, typeset and author-corrected version of record of the manuscript is available on our site with no access controls, subscription charges or pay-per-view fees (http://bmjopen.bmj.com).

If you have any questions on BMJ Open's open peer review process please email info.bmjopen@bmj.com

BMJ Open

Prognostic models for predicting in-hospital paediatric mortality in resource-limited countries: a systematic review

Journal:	*BMJ Open*
Manuscript ID	bmjopen-2019-035045
Article Type:	Original research
Date Submitted by the Author:	21-Oct-2019
Complete List of Authors:	ogero, morris; KEMRI-Wellcome Trust Research Programme,<br>Malla, Lucas; Kenya Medical Research Institute - Wellcome Trust; University of Oxford<br>Aluvaala, Jalemba ; Kenya Medical Research Institute/Wellcome Trust Research Programme, Health Services Unit<br>Agweyu, Ambrose; Kenya Medical Research Institute,<br>Sarguta, Rachel ; University of Nairobi School of Physical Sciences, School of Mathematics<br>English, Mike; University of Oxford, Nuffield Department of Medicine and Department of Paediatrics<br>Onyango, Nelson ; University of Nairobi School of Physical Sciences, School of mathematics<br>Akech, Samuel; KEMRI-Wellcome Trust Research Programme Nairobi
Keywords:	PAEDIATRICS, STATISTICS & RESEARCH METHODS, Community child health < PAEDIATRICS

SCHOLARONE™
Manuscripts

I, the Submitting Author has the right to grant and does grant on behalf of all authors of the Work (as defined in the below author licence), an exclusive licence and/or a non-exclusive licence for contributions from authors who are: i) UK Crown employees; ii) where BMJ has agreed a CC-BY licence shall apply, and/or iii) in accordance with the terms applicable for US Federal Government officers or employees acting as part of their official duties; on a worldwide, perpetual, irrevocable, royalty-free basis to BMJ Publishing Group Ltd ("BMJ") its licensees and where the relevant Journal is co-owned by BMJ to the co-owners of the Journal, to publish the Work in this journal and any other BMJ products and to exploit all rights, as set out in our licence.

The Submitting Author accepts and understands that any supply made under these terms is made by BMJ to the Submitting Author unless you are acting as an employee on behalf of your employer or a postgraduate student of an affiliated institution which is paying any applicable article publishing charge ("APC") for Open Access articles. Where the Submitting Author wishes to make the Work available on an Open Access basis (and intends to pay the relevant APC), the terms of reuse of such Open Access shall be governed by a Creative Commons licence – details of these licences and which Creative Commons licence will apply to this Work are set out in our licence referred to above.

Other than as permitted in any relevant BMJ Author's Self Archiving Policies, I confirm this Work has not been accepted for publication elsewhere, is not being considered for publication elsewhere and does not duplicate material already published. I confirm all authors consent to publication of this Work and authorise the granting of this licence.

For peer review only

Prognostic models for predicting in-hospital paediatric mortality in resource-limited countries: a systematic review

Morris Ogero[1, 2], Lucas Malla[1], Jalemba Aluvaala[1], Ambrose Agweyu[1], Rachel Sarguta[2], Mike English[1, 3], Nelson Onyango[2], Samuel Akech[1]

[1]Kenya Medical Research Institute (KEMRI)-Wellcome Trust Research Programme, Nairobi, Kenya; [2]School of Mathematics, University of Nairobi, Kenya; and [3]Nuffield Department of Medicine, University of Oxford, UK.

Correspondence: Morris Ogero,

KEMRI / Wellcome Trust Research Programme,

P.O Box 43640-00100 Nairobi, Kenya.

mogero@kemri-wellcome.org

For peer review only

Abstract

Objectives: To identify and appraise the methodological rigor of multivariable prognostic models predicting in-hospital paediatric mortality in low- and middle-income countries (LMIC).

Design: Systematic review of peer-reviewed journals.

Data sources: MEDLINE, CINAHL, Google Scholar, and Web of Science electronic databases since inception to August 2019.

Eligibility criteria: We included model development studies predicting in-hospital paediatric mortality in LMIC.

Data extraction and synthesis: This systematic review followed the CHARMS (Checklist for critical Appraisal and data extraction for systematic Reviews of prediction Modelling Studies) framework. The risk of bias assessment was conducted using PROBAST (Prediction model Risk of Bias Assessment Tool). No quantitative summary was conducted due to substantial heterogeneity that was observed after conducting a meta-analysis of the studies included.

Results: Our search strategy identified a total of 4054 unique articles. Among these, 3545 articles were excluded after review of titles and abstracts as they covered non-relevant topics. Full texts of 509 articles were screened for eligibility, of which 15 studies reporting 21 models met the eligibility criteria. Based on the PROBAST tool, risk of bias was assessed in four domains; participant, predictors, outcome, and analyses. The domain of statistical analyses (events per variable, missing data, etc) was the main area of concern where 20/21 models were judged to be of high risk and one model judged to be of unclear risk of bias among the included models.

For peer review only - http://bmjopen.bmj.com/site/about/guidelines.xhtml

Conclusion: This review identified 21 models predicting in-hospital paediatric mortality in LMIC. However, most reports characterising these models are of poor quality when judged against recent reporting standards and have a high risk of bias. Future studies should adhere to standardized methodological criteria and progress from identifying new risk scores to validating or adapting existing scores.

Trial registration number: CRD42018088599

Article summary

Strengths and limitations of this study

- This is the first systematic review on methodological quality of prognostic models predicting in-hospital paediatric mortality in resource-limited settings.
- The review was based on robust search strategy with no language restriction yielding a large number of potentially eligible studies, hence it is unlikely that any potentially eligible study was not included.
- Quality of the included models was assessed based on recent reporting standards which we applied to the identified studies and we also highlighted limitations in the existing prognostic models.
- We relied on what was reported to determine the risk of bias of the models. For instance, if no mention was made of internal validation or even verification of the model assumptions, we assumed they were not done as required in prognostic model development.

For peer review only - http://bmjopen.bmj.com/site/about/guidelines.xhtml

Introduction

Over recent decades, there has been considerable progress in improving child survival[1] but child mortality remains high in sub-Saharan Africa relative to the rest of the world.[2] Paediatric deaths in hospitalized children mostly occur soon after admission,[3] and are caused by common conditions such as malaria, pneumonia, and diarrhoeal diseases among others, which are readily treatable by cost-effective interventions.[3-5] In low- and middle-income countries (LMIC), clinicians often use a set of clinical signs as recommended in the guidelines by World Health Organization (WHO) to identify patients at risk of deterioration while making decisions on appropriate treatment.[6] Clinical criteria recommended by WHO were developed following expert recommendations based on review of evidence from studies reporting risk factors for mortality. Prognostic models, which use statistical equations to predict patients' risk based on the combination of prognostic factors, may improve clinicians' ability to identify high-risk patients and thus improve outcomes.[7]

Various clinical prediction models for hospitalised paediatric patients have been developed over the last 3 decades,[8] however, there are doubts whether appropriate methodology has been used in their development.[9] Notably, none are currently recommended for use in existing paediatric clinical practice guidelines in LMIC and systematic reviews of the methodology used in their development have been strongly recommended.[10] This systematic review addresses this need and aims at identifying and summarizing existing studies reporting prognostic models for predicting in-hospital paediatric mortality in LMIC. Specifically, the research summarises the evidence from the published studies and appraises the methodological rigor of each existing model.

For peer review only - http://bmjopen.bmj.com/site/about/guidelines.xhtml

Methods

Protocol and registration

We registered the protocol for this review at PROSPERO (International Prospective Register of Systematic Reviews) (CRD42018088599).[11] This work is reported as per guidelines by the PRISMA (Preferred Reporting Items for Systematic Reviews and Meta-Analyses).[12]

Eligibility criteria

Reports were eligible for inclusion if they met the following criteria:

1. Study design: peer-reviewed studies whose design was either a randomized controlled trial, cohort (prospective or retrospective), cross-sectional, or case-control observational study.
2. Outcome: studies fitting models predicting all-cause in-hospital mortality in a general paediatric ward were included. Studies predicting post-discharge mortality, trauma or operative mortality were excluded.
3. Target population and setting: studies on children aged over 1-month old admitted in general paediatric wards within LMIC as defined by the World Bank[13] were included. Studies whose predictive models were targeting patients in intensive care unit (ICU) or high dependency unit (HDU) were excluded because these facilities are largely unavailable in low-resource settings. We also excluded studies whose predictive models targeted uncommon conditions in children e.g., chronic kidney disease, cancer, diabetes. However, if a study focused on one of the common childhood illnesses such as malaria, pneumonia, meningitis, anaemia, and diarrhoea/dehydration[3] was included.

For peer review only - http://bmjopen.bmj.com/site/about/guidelines.xhtml

4. Type of studies: we included studies whose main objective was deriving a predictive model(s) or scoring system(s). We excluded commentaries, editorials, expert views, conference proceedings, case reports, case-series, reviews and explanatory studies that mainly generate hypothesis[14].
5. Models: studies that reported multivariable model with at least 2 variables/predictors were included.
6. Full text and language: We excluded studies that were not available in full text. Non-English language studies were translated using Google Translate. Hence no language restriction was made.

Search strategy

As recommended by CHARMS checklist, we came up with seven key items (supplementary file 1 (Table 1)) applicable to our study that guided the framing of the search strategy, review aim and eligibility criteria.

We used Medical Subject Headlines (MeSH) terms where appropriate keywords to identify articles with prognostic models relevant for this review. A search of articles was conducted in MEDLINE, Google Scholar, and CINAHL (via EbscoHost) since inception to August 2019. We also performed a search in Web of Science to identify additional reports that cited the identified studies. Reference lists of all identified articles were searched manually to identify other potentially eligible studies.

We manually searched reference lists of all relevant articles to identify additional eligible studies. Final search results were collated in EndNoteX7™. Detailed search terms and strategy are provided in supplementary file 1(Table 3).

For peer review only - http://bmjopen.bmj.com/site/about/guidelines.xhtml

Screening of articles

Prior to screening titles and abstracts, 2 reviewers (MO and LM) standardized the approach to be used in the process of screening. We used a sample of 30 search results to train and familiarize reviewers with the screening process. Titles and abstracts of the studies were screened by the two reviewers. Discrepancies were resolved via discussion and, when necessary, a final decision was adjudicated by a third reviewer (JA).

Data Extraction

Data were extracted from relevant articles in accordance with the Cochrane Prognosis Methods' guidance; the CHecklist for critical Appraisal and data extraction for systematic Reviews of prediction Modelling Studies (CHARMS).[15] From each study included, data were extracted on participant enrolment, study design, study population characteristics, location, sample size, number and selection of predictors, study dates, handling of continuous predictors, missing data, method of modelling (e.g., logistic regression, survival), verification of model assumptions, internal validation methods (e.g., random split of data, and resampling techniques); model presentation (e.g., nomogram, score chart, or regression formula with coefficients); and model performance metrics including discrimination -area under the receiver operating characteristic curve (AUC) with 95% confidence interval (CI); calibration; classification measures such as sensitivity, specificity, positive, and negative predictive values. We further investigated from literature to determine if included models have been externally validated elsewhere. For articles that described development of multiple prognostic models, we treated each model separately whenever the predictor-outcome association produced different model estimates. For each study, extracted data elements were compared between two reviewers (MO & LM), and any disagreements were resolved by discussion with the third reviewer (JA).

For peer review only - http://bmjopen.bmj.com/site/about/guidelines.xhtml

No quantitative summary was conducted due to substantial heterogeneity that was observed after conducting a meta-analysis of the studies included.

Assessment of methodological quality

The *risk of bias* (shortcomings in the predictive models that might lead to unreliable predictions)[15] of the included studies was assessed based on the Prediction study Risk Of Bias Assessment Tool (PROBAST).[16 17] We assessed the *risk of bias* (RoB) for each model, in four domains: participant selection (e.g. study design), predictors (e.g. assessment of candidate and final model predictors), outcome, and analysis (e.g. handling of missing data, the handling of continuous predictors) see supplementary file 1 (Table 2). For each domain, signalling questions have five possible answers: yes; probably yes; probably no; no; and no information. Any positive answer (yes, or probably yes) suggests low RoB. Each domain had three possible outcomes: low; high; or unclear RoB. Using these domain outcomes, we came up with an overall judgement of RoB for each model. As recommended by PROBAST, if a prediction model was judged as low on all five domains, we assigned it an overall judgment of "low RoB". If a model was rated as high at least in one domain, we judged it as having "high RoB". If at least one domain of the model was rated as unclear and the rest of the domains rated as low, it was judged as having "unclear RoB". If a predictive model was rated as low RoB for all domains, and it has not been subjected to any external validation, we downgraded it to "high RoB".

Patient and public involvement

No patient involved.

For peer review only - http://bmjopen.bmj.com/site/about/guidelines.xhtml

Results

Characteristics of the included studies

Our search strategy identified a total of 4054 unique articles, 3545 articles were excluded after review of titles and abstracts as they reported non-relevant topics. Full texts of 509 articles were screened for eligibility, of which 15 primary studies reporting 21 developed models met the eligibility criteria (Figure 1). The eligible studies analysed data for patients who were below 15 years of age with median mortality being 6.7% (range 1.2% to 43.9%).[18 19] While majority of the models were developed for general cases in paediatric wards (n=9), some were tailored for specific paediatric groups defined by common diagnoses such as febrile illness (n=1),[20] malaria (n=2), [21 22] pneumonia (n=4),[18 23-25] malnutrition (n=2) [26 27] and other infectious diseases (n=3) (see supplementary file 1).

Most of the included studies have been published post year 2000 (n = 20) except for one study [26] published in 1996. The latest data used in the models under review were from 2016 to 2017 by Rosman *et al.*[28] and the oldest data were utilized by Draimax *et al.*[26] from 1986 to 1988.

Five reports of the 15 included studies utilized data from at least two centres of which 3 studies [21] were conducted in multiple countries including sub-Saharan Africa and Asian countries (Figure 2). Of the reviewed studies, most of the information we were abstracting were either not reported or were partially reported, an indication of non-adherence to the TRIPOD (Transparent Reporting of a Multivariable Prognostic Model for Individual Prognosis or Diagnosis) guidelines.[21 22 29 30]

For peer review only - http://bmjopen.bmj.com/site/about/guidelines.xhtml

Summary of issues in methodology of the reviewed models

Candidate predictors

There were 61 distinct predictors used in the final reported models (a median of 7 predictors in any one model). Initial selection of the candidate predictors was mostly based on univariable analysis except for three studies[26] where the selection was based on literature reviews or clinical relevance. Backward stepwise selection method was used in 6 models in a multivariable analysis to determine final model predictors. Commonly included predictors in the final models were: altered consciousness, malnutrition indicators, vital signs, and signs of respiratory distress (see Figure 3). Some models included predictors that were either not easy to obtain or required laboratory techniques.[26] Of the 13 models including continuous predictors, 8 models categorized continuous predictors where a continuous scale would have been possible. Two out of 13 models applied other techniques such as fractional polynomial[29 31 32] and restricted cubic splines[27] to determine the suitable functional form of the continuous predictors (See supplementary file 2).

Sample size, events per variable (EPV) and missing data

Sample size ranged from 168 [28] to 50,249 [33] with a median of 1307. The median EPV was 21(IQR 8.3 – 32.5) of which 7 models had less than 10 EPVs, suggestive of insufficient sample sizes which is prone to over-fitting. For instance, 60 deaths were reported in the dataset used to develop PEDIA-Immediate score in the study by Berkley *et al*.[34] In reference to the rule that a study developing predictive model should have a minimum of 10 events for each independent predictor in a prognostic model,[35] a model with, at the most, 6 predictors should have been considered but 10 predictors were considered instead hence making EPV 6.

For peer review only - http://bmjopen.bmj.com/site/about/guidelines.xhtml

Proportions of missing data was not always reported. Handling of missing data varied across the reviewed studies as follows: 6 models did not report handling of missing data; 8 used complete case analysis; 4 used multiple imputation (using Chained Equations); and one study[27] used single imputation.

Model development

Majority of the studies applied logistic regression, one study[31] used Cox regression, one study[34] used Spiegelhalter/Knill-Jones method and another study[22] used a machine learning technique (classification and regression trees (CRT)) in model development. Verification of model assumptions was not reported in most of the studies. For instance, George *et al*[31] despite utilizing Cox regression to develop their model, did not report the verification of proportional hazard assumption nor explore the possibility of competing risks. Other regression assumptions e.g. multicollinearity was equally not reported. However, since backward elimination method disregards redundant variables, we inferred the satisfaction of multicollinearity assumption if this method was applied.[36] Five studies developed models using data from different countries/centres but none of them clustered their analysis by source of data in a multilevel model to account for heterogeneity. Ignoring clustering leads to a biased predictor effect.[37]

Model performance evaluation & presentation

Performance measures (both calibration and discrimination) were poorly reported in most of the studies and in most cases AUC for discrimination was reported (n=20). Performance of the derived models was evaluated in 12 models using either split-sample, resampling methods, or separate datasets. Except for the model derived by George *et al,*[20] all other models did not report both apparent discrimination (without any adjustment for optimism) and optimism-corrected discrimination measures. Despite inadequate reporting of the models' performance, 16 models

For peer review only - http://bmjopen.bmj.com/site/about/guidelines.xhtml

reported AUCs ≥ 0.80, an indication of promising models. Apart from the following exceptions; *Lambarene Organ Dysfunction* (LOD) score,[21] *Paediatric Early Death Index for Africa* (PEDIA) score,[19] *Signs of Inflammation in Children that Kill* (SICK) score,[38] *Respiratory Index of Severity in Children*(RISC) score,[18] and *Modified Respiratory Index of Severity in Children* (mRISC) score,[38] other scores have not been externally validated (by independent investigators using diverse populations). Only 2 studies[24] developing 4 models provided a full model formula (both coefficients and intercept/baseline function) in their results as recommended.[39 40] While most of the models (n=17) were presented as simplified integer scores, only a few were assigned weights according to the regression coefficients.

Risk of bias (RoB) assessment

Based on the PROBAST tool, RoB was assessed in four domains; participants, predictors, outcome, and analyses. Figure 4 summarizes the RoB assessment across all models included in this review, the domain of outcome was deemed to be of low risk in all models. The domain of statistical analyses was the main area of concern where 20/21 models were judged to be of high risk and one model judged to be of unclear RoB (see Figure 5). Full details are provided in supplementary file 3. Most of the models were downgraded to high RoB because of either inadequate sample size (EPV <10) (n=7/21), poor handling of missing data (n=17/21), or using discouraged techniques to perform model selection such as univariable analysis (n=18/21), a sub-optimal approach in analysing continuous predictors (n=8), and lack of reporting on verification of model assumptions (n=13). Details on each PROBAST criterion (20 signalling questions) across domains are provided in the supplementary file 3.

For peer review only - http://bmjopen.bmj.com/site/about/guidelines.xhtml

Discussion

Summary of key findings

We conducted a systematic review to identify published scores predicting in-hospital mortality for paediatrics in resource-limited countries. Fifteen studies that described the development of 21 prognostic models were identified. We described characteristics of these studies as well as the methodological quality of the included models by using agreed recent guidelines applicable to predictive models. We have identified several important quality deficiencies such as inadequate reporting and other methodological concerns, including poor handling of missing data, automated selection of predictors, categorization of continuous predictors, inadequate EPV and the poor presentation of the proposed model for use. As a result, a majority (21 out of 22) of the included models were found to be of poor methodological quality and consequently judged to potentially high risk of bias in predictions (Figure 5).

Our findings suggest that predictive models fail to meet recently agreed methodological criteria in various ways. Firstly, in this review we observed that univariable analysis was routinely used in 18 out of 21 models in the selection of candidate predictors to be used in a multivariable analysis. This strategy tends to leave out possibly important prognostic factors which might be insignificant in a univariable analysis but turn out to be significant when combined with other predictors.[39 40] *A priori* selection of predictors using expert opinion, clinical intuition or literature is recommended for this purpose,[41 42] however only two models in this review employed this approach.[39 40]

Small sample sizes in model development can lead to poor predictive performance, over-fitting, and biased effect estimates. Prognostic models must have a minimum of 10 events per candidate predictor, as this is the accepted norm[43-45] and underpowered models arising from inadequate

For peer review only - http://bmjopen.bmj.com/site/about/guidelines.xhtml

events per variable (EPV) increase the possibility of spurious results.[35] In this review, 7 of 21 models had inadequate sample sizes (EPV<10) and there was no information on whether bootstrapping, which serves to reduce overfitting was used in these models.[46]

Just like most of the epidemiological studies, missing data is a common problem which is solved using multiple imputation or other reasonable approaches, but this was rarely the case in the model development studies under this review. For instance, 8/21 models used complete case analysis (CCA), 4/21 used multiple imputation, and 6/21models did not report how missing data were handled and therefore we assumed CCA was used. Following Harrell's guidelines,[47] CCA should only be used if the percentage of missingness is < 5% but the appropriateness of the CCA approach could not be ascertained as most of the included studies failed to report the proportion of missing data per variable. Inappropriate use of CCA results in use of only a small subset of the data which cannot be regarded as a random sample from the target population unless data are missing completely at random(MCAR),[48] a mechanism which is rarely in practice.[15] Consequently, there are concerns about possible loss of precision in inferences and the potential biases of the estimated parameters[49] in the models employing CCA. Finally, handling of continuous predictors was a concern in this review. Of the 13 models including continuous predictors, 8 models categorized continuous predictors where a continuous scale would have been possible. While this approach is intuitive to most researchers, its simplicity comes at a considerable cost of predictive performance.[50] The resulting prognostic models have been shown to have poor predictive accuracy because of the loss of statistical power and information. It is recommended that the nature of continuous data should be retained or handled by using other techniques such as regression splines or LOESS functions.[50 51] In this review, appropriate

For peer review only - http://bmjopen.bmj.com/site/about/guidelines.xhtml

methods of transforming continuous data was done by only 2 models which applied fractional polynomial[29 31 32] and restricted cubic splines.[27]

Comparison with Other Studies

Methods used to assess quality measures of the included models in the current study have been applied previously to critically evaluate the quality of predictive models in other specialties.[52-54] Just like the findings of this review, other previous reviews [9 55-57] describing the development of prognostic models highlighted a lot of flaws including inappropriate statistical analyses, poor reporting of important clinical and methodological information needed for validation of the model, and lack of external validations. Incomplete reporting of clinical models stops future studies on prognostic research from building on the information of already existing models. This has been marked as an important source of wasted research efforts.[58] For example, external validation of prognostic models requires a full model formula to enable direct estimation of survival probabilities.[39] However, this was presented in only 4 models. Thus, this review highlights the need for researchers to adhere to the TRIPOD statement while developing prognostic models. Of note, the quality of clinical predictive models does not appear to have improved over time as previous reviews from 1996,[59] 1997,[60] 2001,[61] 2005,[62] 2011,[8] 2012,[63] 2016,[64 65] 2017, [66] to 2019[67] have consistently identified suboptimal methodologies in these predictive models. Poorly derived models may result in overoptimistic results and misleading performance. Presumably there are reasons why many prognostic models are of poor quality, including pressure to publish new predictive model regardless of the clinical value of the resultant model.[68]

For peer review only - http://bmjopen.bmj.com/site/about/guidelines.xhtml

Implications of this study

Most of the included models have not been externally validated despite repeated recommendations that this is needed.[39 40 69-72] This suggests that researchers focus more on deriving new models, often using similar prognostic factors, rather than validating and improving existing in-hospital mortality prediction models. This leaves healthcare policy makers with doubts as to which model to recommend in their setting. Large datasets such as that of the Clinical Information Network (CIN) [3 73 74] now exist in resource-limited settings. Future studies on prognostic research should leverage such datasets to externally validate competing models identified in this review for comparative performances as recommended by Collins *et al,*[75] and if necessary, predictive performance of such models should be improved by addition of new prognostic factors. Even so, the application of these models is likely to be impaired as most of the models reported have simplified the original predictor coefficients. This has an implication on model performance during external validation due to loss in predictive accuracy arising from rounding coefficients to nearest integers.[15]

Strengths and limitations

To our knowledge, this is the first review identifying models predicting in-hospital paediatric mortality in resource-limited settings. Our robust search strategy yielded a large number of potentially eligible studies, hence it is unlikely that any potentially eligible study was not included. The quality of the included models was assessed based on recent reporting standards and applied to the identified studies. For instance, if no mention was made of internal validation or even verification of the model assumptions, it could not be determined whether these crucial steps of model development were carried out or not. Thus, models that could have been

For peer review only - http://bmjopen.bmj.com/site/about/guidelines.xhtml

otherwise rated as low risk of bias were rated as either unclear or high risk of bias. Despite this, we still hold that complete reporting of any proposed model is necessary to facilitate external validation and its subsequent application in practice.

Conclusion

Rigorously developed and robustly validated promising predictive models have the potential for improving child survival in resource-limited countries. This review identified models predicting in-hospital mortality for paediatrics. However, most are of poor quality and have high risk of bias. Our research highlights the need to improve on the identified quality deficiencies when developing prognostic models in the future by adhering to existing generally accepted standardized methodological criteria. Majority of the derived models have not been externally validated as required. Inadequate reporting observed in the included models hinders rigorous external validation by other researchers, leave alone implementing them in practice. Rather than developing new prognostic models, researchers should carry out comprehensive joint external validation of the identified models using large datasets ideally collected over extended time periods and different locations. This will allow head-to-head comparisons and adaptation of the competing models, if necessary, to ascertain their generalizability.

For peer review only

Funding

Funds from The Wellcome Trust (#207522) awarded to ME as a Senior Fellowship together with additional funds from a Wellcome Trust core grant awarded to the KEMRI-Wellcome Trust Research Programme (#092654) supported this work. The funders had no role in drafting or submitting this manuscript.

Author Statement

The roles of the contributors were as follows: ME, SA, and AA conceptualized the study. MO, LM and JA conducted electronic searches to identify eligible models and did analyses. MO drafted the initial manuscript with SA, NO, RS, AA, and ME contributed to its development. All authors read and approved the final manuscript.

Competing interests

The authors declare no conflicts of interest.

Data sharing statement

No additional data are available.

For peer review only

References

1. World Health Organization. Child mortality rates plunge by more than half since 1990 but global MDG target missed by wide margin: WHO; 2015 [Available from: http://www.who.int/mediacentre/news/releases/2015/child-mortality-report/en/
2. Lucia Hug DS, Danzhen You. Levels and Trends in Child Mortality: UNICEF; 2019 [Available from: https://data.unicef.org/resources/levels-and-trends-in-child-mortality/
3. Ayieko P, Ogero M, Makone B, et al. Characteristics of admissions and variations in the use of basic investigations, treatments and outcomes in Kenyan hospitals within a new Clinical Information Network. *Archives of disease in childhood* 2015:archdischild-2015-309269.
4. World Health Organization. Serious childhood problems in countries with limited resources. Geneva2004.
5. World Health Organization. Children: reducing mortality: WHO; 2017 [Available from: http://www.who.int/mediacentre/factsheets/fs178/en/
6. World Health Organization. Handbook IMCI: integrated management of childhood illness. Geneva2005.
7. Vogenberg FR. Predictive and prognostic models: implications for healthcare decision-making in a modern recession. *American health & drug benefits* 2009;2(6):218.
8. Maguire JL, Kulik DM, Laupacis A, et al. Clinical prediction rules for children: a systematic review. *Pediatrics* 2011;128(3):e666-e77. doi: 10.1542/peds.2011-0043
9. Bouwmeester W, Zuithoff NP, Mallett S, et al. Reporting and methods in clinical prediction research: a systematic review. *PLoS medicine* 2012;9(5):e1001221.
10. Altman DG. Systematic reviews in health care: Systematic reviews of evaluations of prognostic variables. *BMJ: British Medical Journal* 2001;323(7306):224.
11. Ogero M, Malla L, Agweyu A, et al. Prognostic models predicting in-hospital mortality of paediatrics in resource-limited countries: systematic review. 2018
12. Moher D, Liberati A, Tetzlaff J, et al. Preferred reporting items for systematic reviews and meta-analyses: the PRISMA statement. *PLoS medicine* 2009;6(7):e1000097.
13. WORLD BANK. World Bank Country and Lending Groups: WOLD BANK; 2019 [Available from: https://datahelpdesk.worldbank.org/knowledgebase/articles/906519-world-bank-country-and-lending-groups
14. Shmueli G. To explain or to predict? *Statistical science* 2010:289-310.
15. Moons KG, de Groot JA, Bouwmeester W, et al. Critical appraisal and data extraction for systematic reviews of prediction modelling studies: the CHARMS checklist. *PLoS medicine* 2014;11(10):e1001744.
16. Wolff RF, Moons KG, Riley RD, et al. PROBAST: a tool to assess the risk of bias and applicability of prediction model studies. *Annals of Internal Medicine* 2019;170(1):51-58.
17. Moons KG, Wolff RF, Riley RD, et al. PROBAST: a tool to assess risk of bias and applicability of prediction model studies: explanation and elaboration. *Annals of Internal Medicine* 2019;170(1):W1-W33.
18. Reed C, Madhi SA, Klugman KP, et al. Development of the Respiratory Index of Severity in Children (RISC) score among young children with respiratory infections in South Africa. *Plos One* 2012;7(1):e27793-e93. doi: 10.1371/journal.pone.0027793
19. Berkley JA, Ross A, Mwangi I, et al. Prognostic indicators of early and late death in children admitted to district hospital in Kenya: cohort study. *BMJ (Clinical Research Ed)* 2003;326(7385):361-61.

For peer review only - http://bmjopen.bmj.com/site/about/guidelines.xhtml

20. George EC, Walker AS, Kiguli S, et al. Predicting mortality in sick African children: the FEAST Paediatric Emergency Triage (PET) Score. *BMC Medicine* 2015;13:174-74. doi: 10.1186/s12916-015-0407-3
21. Helbok R, Kendjo E, Issifou S, et al. The Lambaréné Organ Dysfunction Score (LODS) is a simple clinical predictor of fatal malaria in African children. *Journal of Infectious Diseases* 2009;200(12):1834-41. doi: 10.1086/648409
22. Erdman LK, Dhabangi A, Musoke C, et al. Combinations of host biomarkers predict mortality among Ugandan children with severe malaria: a retrospective case-control study. *Plos One* 2011;6(2):e17440-e40. doi: 10.1371/journal.pone.0017440
23. Emukule GO, McMorrow M, Ulloa C, et al. Predicting mortality among hospitalized children with respiratory illness in Western Kenya, 2009-2012. *Plos One* 2014;9(3):e92968-e68. doi: 10.1371/journal.pone.0092968
24. Hooli S, Colbourn T, Lufesi N, et al. Predicting Hospitalised Paediatric Pneumonia Mortality Risk: An External Validation of RISC and mRISC, and Local Tool Development (RISC-Malawi) from Malawi. *PLoS ONE* 2016;11(12):1-13. doi: 10.1371/journal.pone.0168126
25. Gallagher KE, Knoll MD, Prosperi C, et al. The Predictive Performance of a Pneumonia Severity Score in Human Immunodeficiency Virus–negative Children Presenting to Hospital in 7 Low-and Middle-income Countries. 2019
26. Dramaix M, Brasseur D, Donnen P, et al. Prognostic indices for mortality of hospitalized children in central Africa. *American Journal Of Epidemiology* 1996;143(12):1235-43.
27. Olson D, Davis NL, Milazi R, et al. Development of a severity of illness scoring system (inpatient triage, assessment and treatment) for resource-constrained hospitals in developing countries. *Tropical Medicine & International Health* 2013;18(7):871-78. doi: 10.1111/tmi.12137
28. Rosman SL, Karangwa V, Law M, et al. Provisional validation of a pediatric early warning score for resource-limited settings. *Pediatrics* 2019;143(5):e20183657.
29. von Seidlein L, Olaosebikan R, Hendriksen ICE, et al. Predicting the clinical outcome of severe falciparum malaria in african children: findings from a large randomized trial. *Clinical Infectious Diseases: An Official Publication Of The Infectious Diseases Society Of America* 2012;54(8):1080-90. doi: 10.1093/cid/cis034
30. Conroy ALP, Glover SJMD, Hawkes MMD, et al. Angiopoietin-2 levels are associated with retinopathy and predict mortality in Malawian children with cerebral malaria: A retrospective case-control study*. *Critical Care Medicine* 2012;40(3):952-59.
31. George EC, Walker AS, Kiguli S, et al. Predicting mortality in sick African children: the FEAST Paediatric Emergency Triage (PET) Score. *BMC Medicine* 2015;13(1):1-12. doi: 10.1186/s12916-015-0407-3
32. Hartley M-A, Young A, Tran A-M, et al. Predicting Ebola Severity: A Clinical Prioritization Score for Ebola Virus Disease. *Plos Neglected Tropical Diseases* 2017;11(2):e0005265-e65. doi: 10.1371/journal.pntd.0005265
33. Mpimbaza A, Sears D, Sserwanga A, et al. Admission Risk Score to Predict Inpatient Pediatric Mortality at Four Public Hospitals in Uganda. *Plos One* 2015;10(7):e0133950-e50. doi: 10.1371/journal.pone.0133950
34. Lowlaavar N, Larson CP, Kumbakumba E, et al. Pediatric in-Hospital Death from Infectious Disease in Uganda: Derivation of Clinical Prediction Models. *Plos One* 2016;11(3):e0150683-e83. doi: 10.1371/journal.pone.0150683
35. Concato J, Feinstein AR, Holford TR. The risk of determining risk with multivariable models. *Annals of internal medicine* 1993;118(3):201-10.
36. Vijay Kotu BD. Feature Selection. In: Vijay Kotu BD, ed. Data Science Concepts and Practice2019:467-90.

For peer review only - http://bmjopen.bmj.com/site/about/guidelines.xhtml

37. Debray T, Moons KG, Ahmed I, et al. A framework for developing, implementing, and evaluating clinical prediction models in an individual participant data meta-analysis. *Statistics in medicine* 2013;32(18):3158-80.
38. Kumar N, Thomas N, Singhal D, et al. Triage score for severity of illness. *Indian pediatrics* 2003;40(3):204-10.
39. Moons KGMP, Altman DGD, Reitsma JBMDP, et al. Transparent Reporting of a multivariable prediction model for Individual Prognosis Or Diagnosis (TRIPOD): Explanation and Elaboration. *Annals of Internal Medicine* 2015;162(1):W1-W73.
40. Collins GSP, Reitsma JBMDP, Altman DGD, et al. Transparent Reporting of a multivariable prediction model for Individual Prognosis Or Diagnosis (TRIPOD): The TRIPOD Statement. *Annals of Internal Medicine* 2015;162(1):55-63.
41. Justice AC, Covinsky KE, Berlin JA. Assessing the generalizability of prognostic information. *Annals of internal medicine* 1999;130(6):515-24.
42. Kattan MW, Harrell FE. We should not be so quick to abandon the use of domain experts and full models (letter commenting: J Clin Epidemiol. 2015; 71C: 76--85.). *Journal of clinical epidemiology* 2016;75:131.
43. Concato J, Peduzzi P, Holford TR, et al. Importance of events per independent variable in proportional hazards analysis I. Background, goals, and general strategy. *Journal of clinical epidemiology* 1995;48(12):1495-501.
44. Peduzzi P, Concato J, Feinstein AR, et al. Importance of events per independent variable in proportional hazards regression analysis II. Accuracy and precision of regression estimates. *Journal of clinical epidemiology* 1995;48(12):1503-10.
45. Peduzzi P, Concato J, Kemper E, et al. A simulation study of the number of events per variable in logistic regression analysis. *Journal of clinical epidemiology* 1996;49(12):1373-79.
46. A bootstrapping approach to reduce over-fitting in genetic programming. Proceedings of the 15th annual conference companion on Genetic and evolutionary computation; 2013. ACM.
47. Harrell FE. Regression Modeling Strategies. *BIOS* 2014;330
48. White IR, Royston P, Wood AM. Multiple imputation using chained equations: Issues and guidance for practice. *Stat Med* 2011;30(4):377-99. doi: 10.1002/sim.4067
49. Molenberghs G, Fitzmaurice G, Kenward MG, et al. Handbook of missing data methodology: CRC Press 2014.
50. Collins GS, Ogundimu EO, Cook JA, et al. Quantifying the impact of different approaches for handling continuous predictors on the performance of a prognostic model. *Statistics in medicine* 2016;35(23):4124-35.
51. Royston P, Altman DG, Sauerbrei W. Dichotomizing continuous predictors in multiple regression: a bad idea. *Statistics in medicine* 2006;25(1):127-41.
52. Ensor J, Riley RD, Moore D, et al. Systematic review of prognostic models for recurrent venous thromboembolism (VTE) post-treatment of first unprovoked VTE. *BMJ open* 2016;6(5):e011190.
53. Smit HA, Pinart M, Antó JM, et al. Childhood asthma prediction models: a systematic review. *The Lancet Respiratory Medicine* 2015;3(12):973-84. doi: 10.1016/S2213-2600(15)00428-2
54. Hodgson LE, Sarnowski A, Roderick PJ, et al. Systematic review of prognostic prediction models for acute kidney injury (AKI) in general hospital populations. *BMJ open* 2017;7(9):e016591.
55. Collins GS, de Groot JA, Dutton S, et al. External validation of multivariable prediction models: a systematic review of methodological conduct and reporting. *BMC medical research methodology* 2014;14(1):40.
56. Collins GS, Omar O, Shanyinde M, et al. A systematic review finds prediction models for chronic kidney disease were poorly reported and often developed using inappropriate methods. *Journal of clinical epidemiology* 2013;66(3):268-77.

For peer review only

For peer review only - http://bmjopen.bmj.com/site/about/guidelines.xhtml

57. Fahey M, Crayton E, Wolfe C, et al. Clinical prediction models for mortality and functional outcome following ischemic stroke: A systematic review and meta-analysis. *PloS one* 2018;13(1):e0185402.
58. Glasziou P, Altman DG, Bossuyt P, et al. Reducing waste from incomplete or unusable reports of biomedical research. *The Lancet* 2014;383(9913):267-76.
59. Kwakkel G, Wagenaar RC, Kollen BJ, et al. Predicting disability in stroke—a critical review of the literature. *Age and ageing* 1996;25(6):479-89.
60. Laupacis A, Sekar N. Clinical prediction rules: a review and suggested modifications of methodological standards. *Jama* 1997;277(6):488-94.
61. Counsell C, Dennis M. Systematic review of prognostic models in patients with acute stroke. *Cerebrovascular diseases* 2001;12(3):159-70.
62. Jacob M, Lewsey JD, Sharpin C, et al. Systematic Review and Validation of Prognostic Models in Liver Transplantation. *Liver Transplantation* 2005;11(7):814-25.
63. Moons KGM, Kengne AP, Grobbee DE, et al. Risk prediction models: II. External validation, model updating, and impact assessment. *Heart* 2012;98(9):691-98.
64. Damen JA, Hooft L, Schuit E, et al. Prediction models for cardiovascular disease risk in the general population: systematic review. *Bmj* 2016;353:i2416.
65. Cruz GD, Strathe AB, Rossow HA, et al. Characterizing bovine adipocyte distribution and its relationship with carcass and meat characteristics using a finite mixture model. *J Anim Sci* 2012;90(9):2995-3002. doi: 10.2527/jas.2011-4838
66. Singendonk MMJ, Tabbers MM, Benninga MA, et al. Pediatric Gastroesophageal Reflux Disease: Systematic Review on Prognosis and Prognostic Factors. *Journal Of Pediatric Gastroenterology And Nutrition* 2017 doi: 10.1097/MPG.0000000000001697
67. Carrillo-Larco RM, Altez-Fernandez C, Pacheco-Barrios N, et al. Cardiovascular Disease Prognostic Models in Latin America and the Caribbean: A Systematic Review. *Global heart* 2019;14(1):81-93.
68. Vickers AJ, Cronin AM. Everything you always wanted to know about evaluating prediction models (but were too afraid to ask). *Urology* 2010;76(6):1298-301.
69. Ewout W. Steyerberg KGMM, Danielle A. van der Windt3 Jill A. Hayden, Pablo Perel,, Sara Schroter RDR, Harry Hemingway, Douglas G. Altman*, for the PROGRESS Group. Prognosis Research Strategy (PROGRESS) 3: Prognostic Model Research. *PLOS Medicine* 201;10(2) doi: 10.1371/journal.pmed.100138110.1136/bmj.e559510.1371/jour10.1136/bmj.e5793
70. Bleeker S, Moll H, Steyerberg E, et al. External validation is necessary in prediction research:: A clinical example. *Journal of clinical epidemiology* 2003;56(9):826-32.
71. Altman DG, Royston P. What do we mean by validating a prognostic model? *Statistics in medicine* 2000;19(4):453-73.
72. Steyerberg EW, Vergouwe Y. Towards better clinical prediction models: seven steps for development and an ABCD for validation. *European heart journal* 2014:ehu207.
73. Tuti T, Bitok M, Malla L, et al. Improving documentation of clinical care within a clinical information network: an essential initial step in efforts to understand and improve care in Kenyan hospitals. *BMJ global health* 2016;1(1):e000028.
74. Gachau S, Ayieko P, Gathara D, et al. Does audit and feedback improve the adoption of recommended practices? Evidence from a longitudinal observational study of an emerging clinical network in Kenya. *BMJ Global Health* 2017;2(4):e000468.
75. Collins GS, Moons KG. Comparing risk prediction models: British Medical Journal Publishing Group, 2012.

For peer review only - http://bmjopen.bmj.com/site/about/guidelines.xhtml

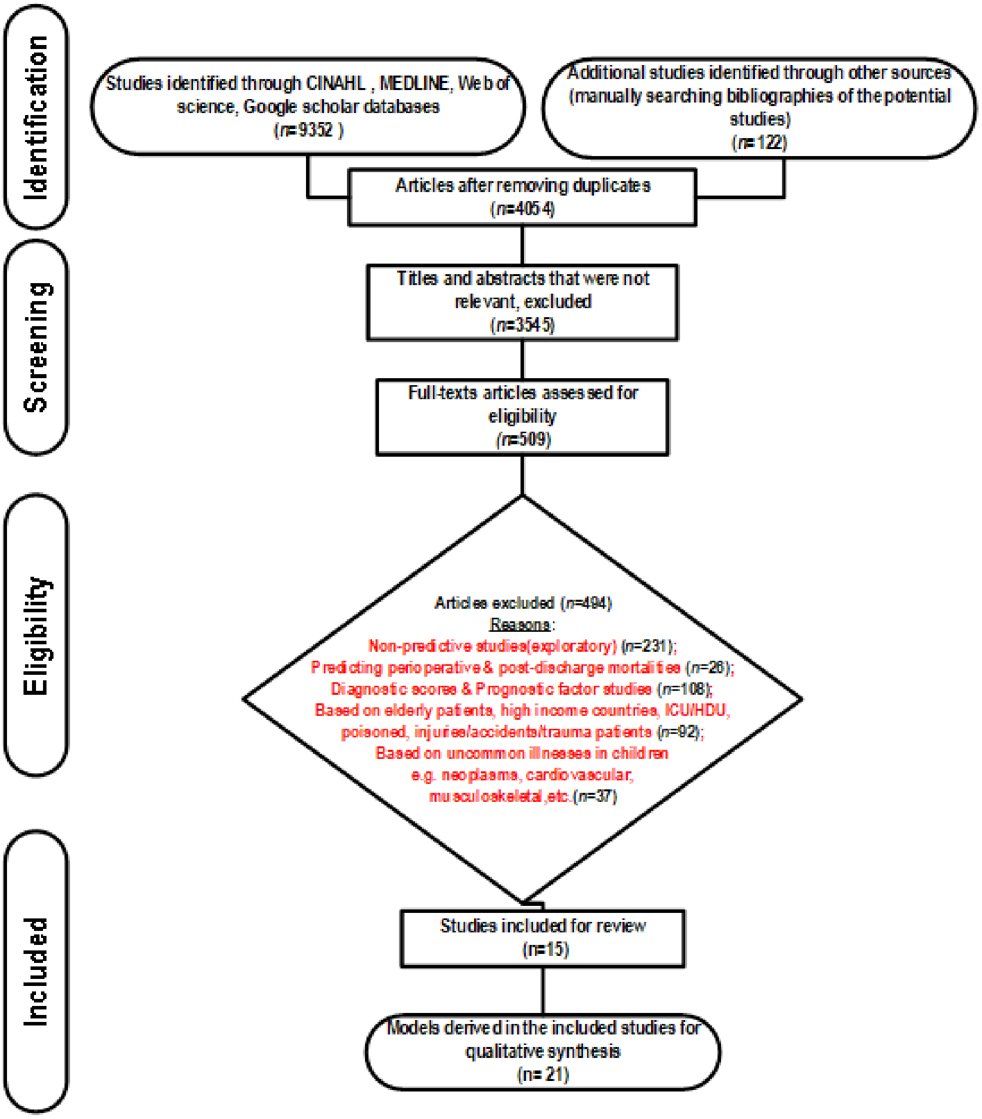

Figure 1: PRISMA flow diagram showing procedure used to identify and select pediatric prognostic models reviewed.

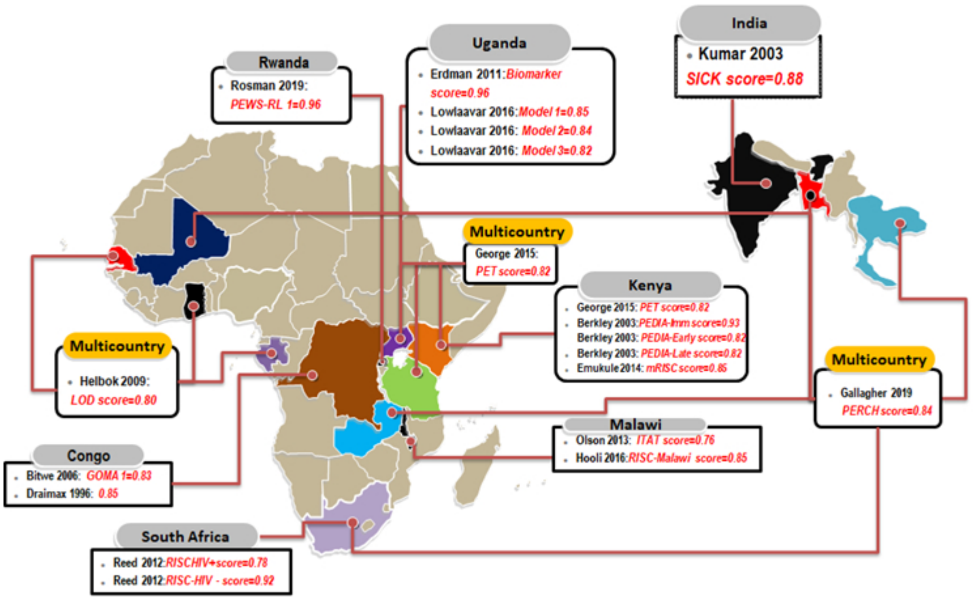

Figure 2: Included prognostic models by country. Text highlighted in red are the names of the models with their corresponding discrimination measures. Key: PEWS-RL score=Paediatric Early Warning Score for Resource-Limited Settings; SICK score=Signs of Inflammation in Children that Kill; PET score= Paediatric Emergency Triage; mRISC score= Modified Respiratory Index of Severity in Children score; RISC score= Respiratory Index of Severity in Children score; PERCH severity score= Pneumonia Etiology Research for Child Health severity score; LOD score= Lambarene Organ Dysfunction score; CRT= Classification and Regression Trees; ITAT Score= Inpatient Triage Assessment and Treatment score; PEDIA score= Paediatric Early Death Index for Africa score

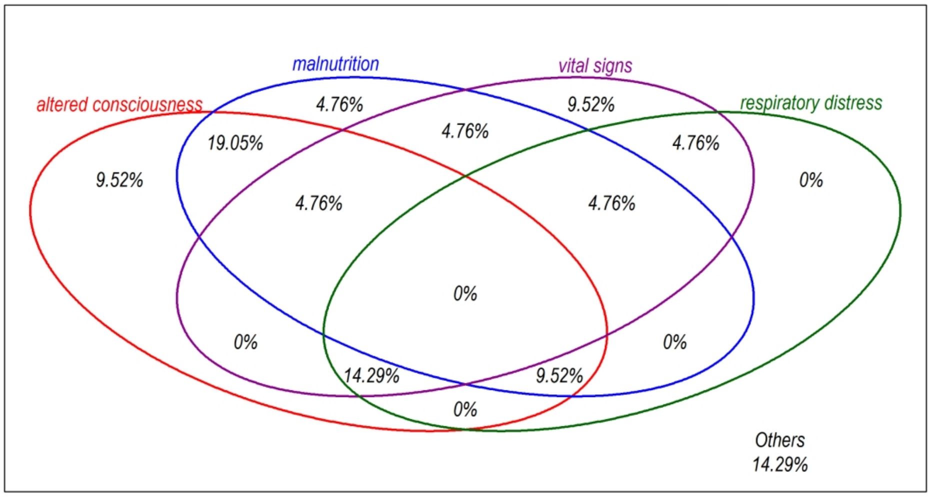

Figure 3: Top four categories of predictors in the models of the reviewed reports: altered consciousness (coma, prostration, not alert, unconscious); malnutrition indicators (kwashiorkor, edema, weight-for-height z-score, weight-for-age z-score, mid-upper arm circumference-MUAC, wasting); vital signs (temperature, respiratory rate, heart rate, oxygen saturation); signs of respiratory distress (indrawing, lung crepitation, difficult breathing, grunting).

228x127mm (300 x 300 DPI)

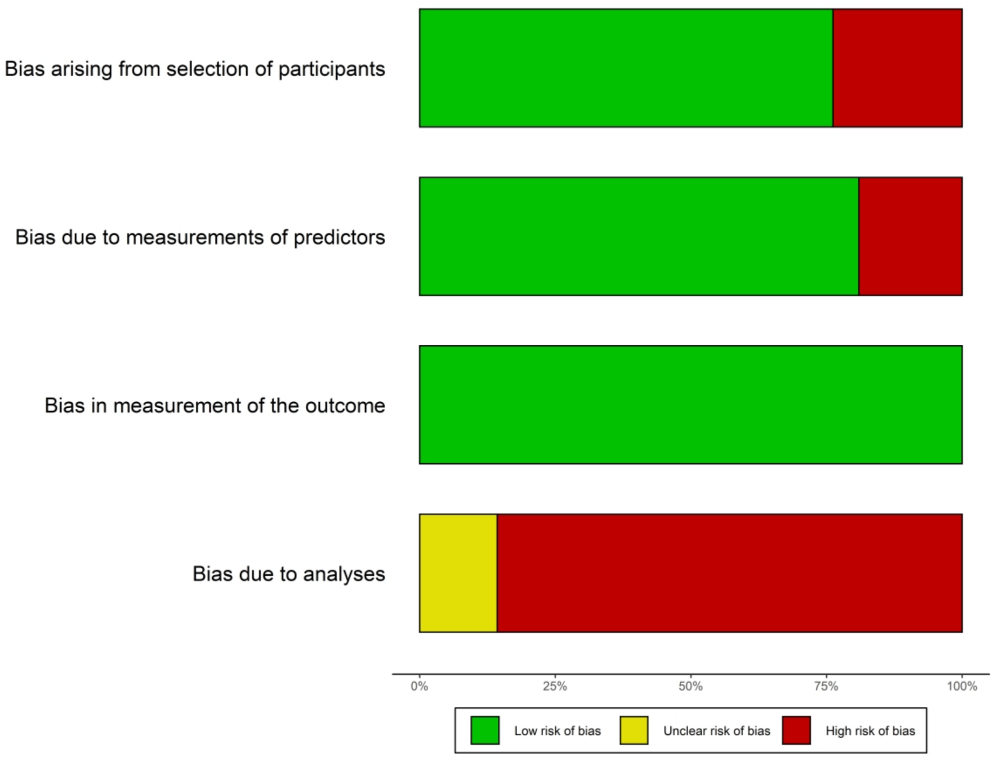

Figure 4: Summary of the risk of bias of the included models using PROBAST.

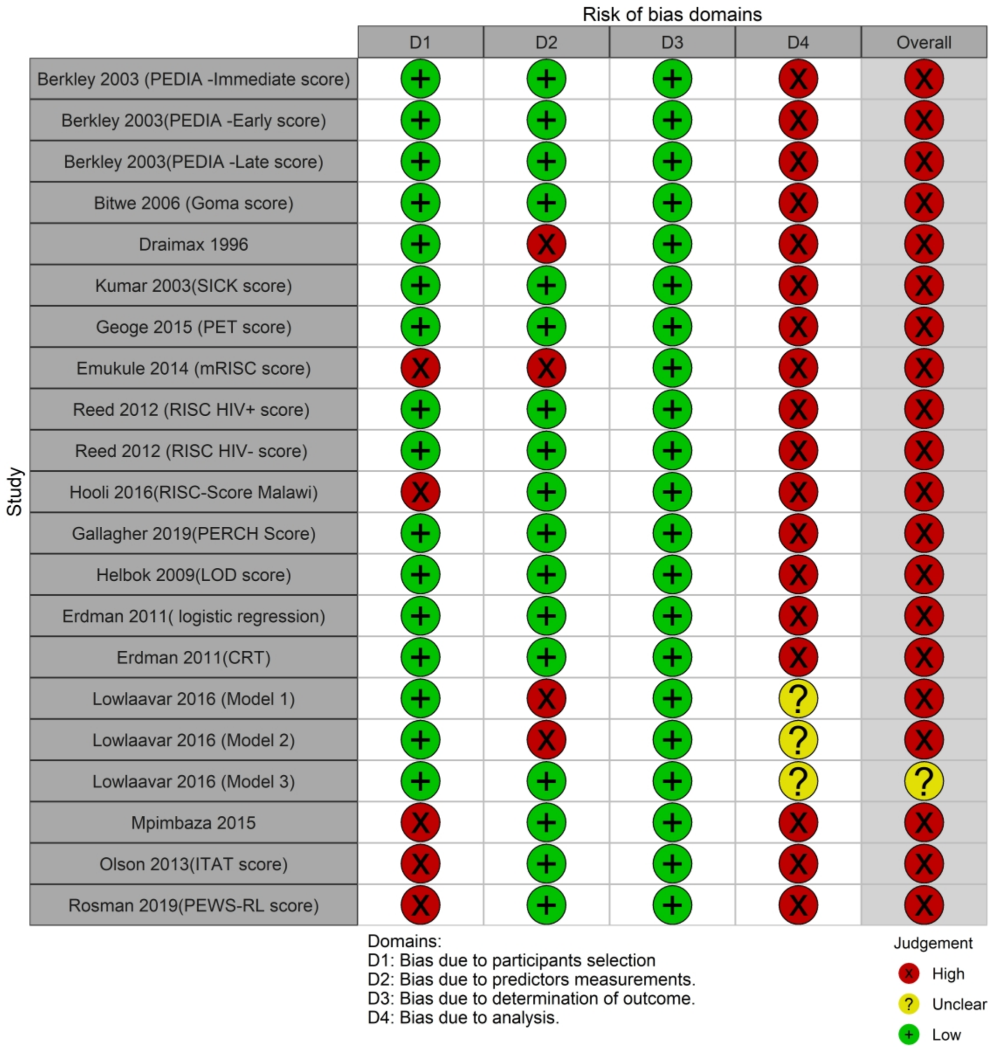

Figure 5: Risk of bias assessment. Low means low risk of bias, High means high risk of bias, and Unclear when it was not possible to assess the risk of bias.
Key: PEWS-RL score=Paediatric Early Warning Score for Resource-Limited Settings; SICK score=Signs of Inflammation in Children that Kill; PET score= Paediatric Emergency Triage; mRISC score= Modified Respiratory Index of Severity in Children score; RISC score= Respiratory Index of Severity in Children score; PERCH severity score= Pneumonia Etiology Research for Child Health severity score; LOD score= Lambarene Organ Dysfunction score; CRT= Classification and Regression Trees; ITAT Score= Inpatient Triage Assessment and Treatment score; PEDIA score= Paediatric Early Death Index for Africa score

Table 1: Review framework according to the CHARMS checklist

Item	Criteria
Prognostic or diagnostic model	Prognostic model predicting in-hospital mortality.
Scope	Prognostic models to inform clinicians about the risk of deterioration or death.
Type of prediction models	Prognostic models with and/or without external validation.
Prediction target population	Children aged > 1 month to 15 years admitted in pediatric wards in developing countries
Outcome of interest	All-cause in-hospital mortality.
Prediction period	Any
Intended moment to apply the prediction tool	Prognostic model to be used in primary prevention to assess risk of deterioration and thus guide prevention/treatment.

For peer review only

Table 2: List of domains and signaling questions used for risk of bias assessment.

Domain	Signalling question
Participants selection	Were appropriate data sources used, e.g., cohort, RCT, or nested case–control study data?
	Were all inclusions and exclusions of participants appropriate?
Predictors	Were predictors defined and assessed in a similar way for all participants?
	Were predictor assessments made without knowledge of outcome data?
	Are all predictors available at the time the model is intended to be used?
Outcome	Was the outcome determined appropriately?
	Was a prespecified or standard outcome definition used?
	Were predictors excluded from the outcome definition?
	Was the outcome defined and determined in a similar way for all participants?
	Was the outcome determined without knowledge of predictor information?
	Was the time interval between predictor assessment and outcome determination appropriate?
Analysis	Were there a reasonable number of participants with the outcome?
	Were continuous and categorical predictors handled appropriately?
	Were all enrolled participants included in the analysis?
	Were participants with missing data handled appropriately?
	Was selection of predictors based on univariable analysis avoided?
	Were complexities in the data (e.g., censoring, competing risks, sampling of control participants) accounted for appropriately?
	Were relevant model performance measures evaluated appropriately?
	Were model overfitting, underfitting, and optimism in model performance accounted for?
	Do predictors and their assigned weights in the final model correspond to the results from the reported multivariable analysis?

For peer review only

Table 3: Search terms

Search ID	Sub-heading	Search Terms
S4	Children	paediatric* OR pediatric* OR (MH “Pediatrics+”) OR child*
S3	Hospital based	(MH “Hospitals+”) OR hospital*
S2	Low-income countries	(MH “Developing Countries+”) OR (MH “Africa+") OR TI (“low income” OR “low and middle income“OR “LMIC” OR “LIC” OR “limited resource*” OR “poor resource*” OR "resource* poor" OR (“developing countries”) OR (“developing nations”) OR (“third world”) OR “resource-constrained” OR (“global south”)
S1	Predictive models	prognos* OR (MH “prognosis”) OR (Predict* AND (Outcome* OR Risk* OR Model* OR Mortality OR Index OR Rule* OR decision* OR scor*)) OR “risk score” OR “scor* system” OR “logistic model*” “risk prediction” OR “risk calculation” OR “risk assessment” OR “c statistic” OR discrimination OR calibration OR AUC OR “area under the curve” OR “area under the receiver operator characteristic curve”

For peer review only

Study	Model name	Country	Source of data	Study year	Inclusion criteria	Age
Berkley 2003	A Immediate	Kenya	Prospective cohort	1998-2000	Aged over 90 days	onths-13 y
Berkley 2003	DIA Early de	Kenya	Prospective cohort	1998-2000	Aged over 90 days	onths-13 y
Berkley 2003	EDIA Late dea	Kenya	Prospective cohort	1998-2000	Aged over 90 days	onths-13 y
Bitwe 2006	Goma 1 Mode	atic Republic c	Prospective cohort	2003-2004	<12 months	an: 12.8 m
Draimax 1996		Congo	Prospective cohort	1986-1988	Malnutrition	ian: 27 mo
Kumar 2003	SICK score	India	Prospective cohort	1998-1999	Paediatric patients	Informati
Geoge 2015	PET Score	a, Uganda, Ta	RCT	2009-2011	Malaria	: 24 (IQR=
Emukule 2014	mRISC	Kenya	Surveillance	2009-2012	spitalized with severe acute	<59 month
Reed 2012	SC-HIV posit	South Africa	RCT	1998-2001	zations under 24 months wit	<24 month
Reed 2012	SC-HIV Nega	South Africa	RCT	1998-2001	tions under 24 months with	<24 month
Hooli 2016	RISC-Malawi	Malawi	pective observationa	2011-2014	onths hospitalized with pne	<59 month
Gallagher 2019	PERCH Score	ca, Mali,Gam	Case-control study	2011–2014	ve hospitalized with severe c	n: 9(4-19)
Helbok 2009	LOD score	lawi,Kenya,G	Prospective cohort	2000-2005	alized children with severe r	28(0-180)
Erdman 2011 (Logistic regression)	iomarker sco	Uganda	tive nested case-co	2007-2009	6 months - 12 years	onths - 12
Erdman 2011 (Classification tree)		Uganda	tive nested case-co	2007-2009	6 months - 12 years	onths - 12
Lowlaavar 2016	Model 1	Uganda	ective observational	2012-2013	nths admitted with infectio	IQR 11.9-
Lowlaavar 2016	Model 2	Uganda	ective observational	2012-2013	nths admitted with infectio	IQR 11.9-
Lowlaavar 2016	Model 3	Uganda	ective observational	2012-2013	nths admitted with infectio	IQR 11.9-

For peer review only

Mpimbaza 2015		Uganda	Surveillance	2010-2013	General paediatrics	nths (IQR
Olson 2013	ITAT score	Malawi	Nested case–control	2010-2011	; on the acute care and maln	≤15 years
Rosman 2019	PEWS-RL	Rwanda	Case-control study	2016-2017	patients admitted to pediatric departme	

For peer review only

Ouctome	Sample size	Number of outcome events	Missing data handling	r of participant with missing data re	Regression methoc
Mortality	429	60	No Information	No Information	lhalter/Knill-Jones r
Mortality	439	193	No Information	No Information	lhalter/Knill-Jones r
Mortality	436	183	No Information	No Information	lhalter/Knill-Jones r
Mortality	414	66	No Information	No Information	Logistic regression
Mortality	1129	196	No Information	No Information	Logistic regression
Mortality	1099	44	No Information	No Information	Logistic regression
Mortality	3170	315	Complete case analyses	Yes	portional hazards re
Mortality	3581	218	Complete case analyses	No Information	Logistic regression
Mortality	1502	265	Complete case analyses	No Information	Logistic regression
Mortality	2646	33	Complete case analyses	No Information	Logistic regression
Mortality	14665	464	Multiple imputation	Yes	Logistic regression
d 7-days p	1802	120	Complete case analyses	No Information	Logistic regression
Mortality	23980	1004	Complete case analyses	Yes	Logistic regression
Mortality	103	23	No missing values	Yes	Logistic regression
Mortality	103	23	No missing values	Yes	Classification tree
Mortality	1307	65	Multiple imputation	No Information	Logistic regression
Mortality	1307	65	Multiple imputation	No Information	Logistic regression
Mortality	1307	65	Multiple imputation	No Information	Logistic regression

For peer review only

Mortality	50249	1742	Complete case analyses	Yes	Logistic regression
Mortality	1606	54	Single imputation	Yes	Logistic regression
nt	168	57	Complete case analyses	No Information	Logistic regression

For peer review only

Vere model assumptions verifie	Predictor selection	Vas a shrinkage method use	bration me	Discriminatio	Classification measures reported
Yes	Univariate	No Information	ɔ Informatio	0.93(0.92-0.94)	No Information
Yes	Univariate	No Information	ɔ Informatio	0.82(0.80-0.83)	No Information
Yes	Univariate	No Information	ɔ Informatio	0.82(0.81-0.84)	No Information
Yes	nivariate & Stepwi	No Information	Yes	).83 (0.78-0.88	No Information
No Information	A priori	No Information	ɔ Informatio	5(No informati	es 40% and negative pr
No Information	ncluded all variable	No Information	ɔ Informatio	0.89	a score of 2.5 with a se
No Information	A priori	No Information	emeshow te	0.82(0.77–0.87)	No Information
Yes	Univariate	Yes	alibration pl	0.85	a sensitivity of 1.8% an
No Information	Univariate	No Information	emeshow te	0.78	sensitivity of 4% and s
No Information	Univariate	No Information	emeshow te	0.92	ıs a sensitivity: 16% Sp
Yes	A priori	No Information	redictivenes	95% CI: 0.76	sensitivity of 57% and s
No Information	Univariate	No Information	alibration pl	4(No Informat	alue 23.6%, positive pr
No Information	ard & backward Ste	No Information	ɔ Informatio	80 (79–82)	itivity was 85% and spe
Yes	Univariate	No Information	est and cali	).96(0.90–0.9999.9)	and specificity o
No Information	No Information	No Information	ɔ Informatio	No Information	d 92.5% specificity for
No Information	nivariate & Stepwi	No Information	ɔ Informatio	.85 (0.80–0.8	9.74–0.92), Specificity:
No Information	nivariate & Stepwi	No Information	ɔ Informatio	.84 (0.79–0.8	9.70–0.90), Specificity:
No Information	nivariate & Stepwi	No Information	ɔ Informatio	.82 (0.72–0.9	).72–0.91), Specificity:

No Information	Backward	No Information	ıeral paedia	6(No informati	No Information
Yes	Univariate	No Information	ɔ Informati	6(No informati	ty: 0.86, PPV: 0.18, NI
No Information	Univariate	No Information	ɔ Informati	(95% CI 0.93–	ısitivity was 96.2%, and

For peer review only

Method used for internal validation	External validation	Was a simplified model presented	Were coefficients(including intercept) of the regression model presented	Number of predictors in final model
Separate dataset	Yes	Yes	NA	10
Separate dataset	Yes	Yes	NA	8
Separate dataset	Yes	Yes	NA	9
No Information	No	Yes	No	8
Separate dataset	No	Yes	No	4
No Information	Yes	Yes	No	9
Separate dataset	No	Yes	No	11
Bootstrapping	Yes	Yes	No	9
Bootstrapping	No	Yes	No	7
Bootstrapping	Yes	Yes	No	5
No Information	No	Yes	Yes	5
apping & separate	No	Yes	No	12
No Information	Yes	Yes	No	8
Boostrappling	No	Yes	No	8
-fold cross validati	No	No	No	3
No Information	No	No	Yes	3
No Information	No	No	Yes	3
No Information	No	No	Yes	2

For peer review only

Separate dataset	No	Yes	No	13
No Information	No	Yes	No	4
No Information	No	Yes	No	6

For peer review only

Predictors in the final model	Are there laboratory based predictors	Handling of continous predictors	Events per variable
out seizures, Impaired consciousness with se	No	NA	6
hout seizures, Impaired consciousness with s	No	NA	24.125
s with seizures, Impaired consciousness with	No	otomized H	20.33333
nconscious, Aware), Infectious diagnosis(A	No	Brachial pe	8.25
MUAC, edema, Serum albumin, Transthyreti	Yes	MUAC	49
olic blood pressure(Normal, Abnormal), Cap	No	nized most	4.888889
<2sec), Conscious level(prostrate, coma), R	No	e fractional	28.63636
to drink/breastfeed, Night sweats, Chest wal	Yes	ized weigh	24.22222
fusing feeds, HIV classification(Severe, Mil	No		37.85714
heezing, Refusing feeds, Weight for age(Lo	No	ized weigh	6.6
Oxygen saturation(moderate, severe), MUAC(moderate, severe), Gender, Wheeze, Consciousness	No	JAC and O	92.8
esponsive and deep breathing), cough, grunt	No	ized most	10
hing, intercostal recession, Coma, Prostratic	Yes	NA	125.5
I-1, soluble Flt-1, procalcitonin, IP-10, solul	Yes	NA	2.875
IP-10, Ang-2, sICAM-1	Yes	NA	7.666667
al BCS, Positive HIV diagnosis, Weight-age	Yes	ed as conti	21.66667
Abnormal BCS, HIV diagnosis, MUAC	Yes	ed as conti	21.66667
Abnormal BCS, MUAC	No	ed as conti	32.5

For peer review only

Age, fever, difficulty breathing, altered consciousness, unable to drink or breastfeed, convulsions, temperature, unconsciousness, pallor, jaundice, deep breathing, meningeal signs, unable to sit up	No	NA	134
saturation, Temperature, Heart rate, Respira	No	Jsed spline	13.5
PEWS-RL score(0 to 6)	No	NA	9.5

For peer review only

Signaling Questions	Study	Participants	
		Were appropriate data sources used, e.g., cohort, RCT, or nested case–control study data?	Were all inclusions and exclusions of participants appropriate?
	Berkley 2003 (PEDIA -Immed	Yes	Yes
	Berkley 2003(PEDIA -Early	Yes	Yes
	Berkley 2003(PEDIA -Late	Yes	Yes
	Bitwe 2006 (Goma score)	Yes	Yes
	Draimax 1996	Yes	Yes
	Kumar 2003(SICK score)	Yes	Yes
	Geoge 2015 (PET score)	Yes	Yes
	Emukule 2014 (mRISC scor	No	Yes
	Reed 2012 (RISC HIV+)	Yes	Yes
	Reed 2012 (RISC HIV-)	Yes	Yes
	Hooli 2016(RISC-Malawi)	No	Yes
	Gallagher 2019(PERCH Sco	Yes	Yes
	Helbok 2009(LOD score)	Yes	Yes
	Erdman 2011(logistic regress	Yes	Yes
	Erdman 2011(CRT)	Yes	Yes
	Lowlaavar 2016 (Model 1)	Yes	Yes
	Lowlaavar 2016 (Model 2)	Yes	Yes
	Lowlaavar 2016 (Model 3)	Yes	Yes
	Mpimbaza 2015	No	Yes
	Olson 2013(ITAT score)	Yes	Yes
	Rosman 2019(PEWS-RL sco	No	Yes

For peer review only

	Predictors			
Risk of Bias in participants	Were predictors defined and assessed in a similar way for all participants	Were predictor assessments made without knowledge of outcome data?	Are all predictors available at the time the model is intended to be used?	Risk of Bias in predictors
Low	Yes	Yes	Yes	Low
Low	Yes	Yes	Yes	Low
Low	Yes	Yes	Yes	Low
Low	Yes	Yes	Yes	Low
Low	Yes	Yes	No	High
Low	Yes	Yes	Yes	Low
Low	Yes	Yes	Yes	Low
High	Yes	Yes	No	High
Low	Yes	Yes	Yes	Low
Low	Yes	Yes	Yes	Low
High	Yes	Yes	Yes	Low
Low	Yes	Yes	Yes	Low
Low	Yes	Yes	Yes	Low
Low	Yes	Yes	Yes	Low
Low	Yes	Yes	Yes	Low
Low	Yes	Yes	No	High
Low	Yes	Yes	No	High
Low	Yes	Yes	Yes	Low
High	Yes	Yes	Yes	Low
Low	Yes	Yes	Yes	Low
High	Yes	Yes	Yes	Low

For peer review only

Outcome

Was the outcome determined appropriately?	Was a prespecified or standard outcome definition used?	Were predictors excluded from the outcome definition?	Was the outcome defined and determined in a similar way for all participants?	Was the outcome determined without knowledge of predictor information?
Yes	Yes	Yes	Yes	Yes
Yes	Yes	Yes	Yes	Yes
Yes	Yes	Yes	Yes	Yes
Yes	Yes	Yes	Yes	Yes
Yes	Yes	Yes	Yes	Yes
Yes	Yes	Yes	Yes	Yes
Yes	Yes	Yes	Yes	Yes
Yes	Yes	Yes	Yes	Yes
Yes	Yes	Yes	Yes	Yes
Yes	Yes	Yes	Yes	Yes
Yes	Yes	Yes	Yes	Yes
Yes	Yes	Yes	Yes	Yes
Yes	Yes	Yes	Yes	Yes
Yes	Yes	Yes	Yes	Yes
Yes	Yes	Yes	Yes	Yes
Yes	Yes	Yes	Yes	Yes
Yes	Yes	Yes	Yes	Yes
Yes	Yes	Yes	Yes	Yes
Yes	Yes	Yes	Yes	Yes
Yes	Yes	Yes	Yes	Yes
Yes	Yes	Yes	Yes	Yes

For peer review only

Was the time interval between predictor assessment and outcome determination appropriate?	Risk of Bias in outcome	Were there a reasonable number of participants with the outcome?	Were continuous and categorical predictors handled appropriately?
Yes	Low	No	Yes
Yes	Low	Yes	Yes
Yes	Low	Yes	No
Yes	Low	No	No
Yes	Low	Yes	No
Yes	Low	No	No
Yes	Low	Yes	Yes
Yes	Low	Yes	No
Yes	Low	Yes	Yes
Yes	Low	No	No
Yes	Low	Yes	No
Yes	Low	Yes	No
Yes	Low	Yes	Yes
Yes	Low	No	Yes
Yes	Low	No	Yes
Yes	Low	Yes	Yes
Yes	Low	Yes	Yes
Yes	Low	Yes	Yes
Yes	Low	Yes	Yes
Yes	Low	Yes	Yes
Yes	Low	No	Yes

For peer review only

			Analysis
Were all enrolled participants included in the analysis?	Were participants with missing data handled appropriately?	Was selection of predictors based on univariable analysis avoided	Were complexities in the data (e.g., censoring, competing risks, sampling of control participants) accounted for appropriately?
Yes	Probably No	No	No Information
Yes	Probably No	No	No Information
Yes	Probably No	No	No Information
Yes	No Information	No	No Information
Yes	No Information	Yes	No Information
Yes	No Information	Yes	No Information
Yes	No	Yes	No
Yes	No	No	No Information
Yes	No Information	No	No Information
Yes	No Information	No	No Information
Yes	Yes	Yes	No Information
Yes	No	No	No Information
Yes	No	Yes	No Information
Yes	Yes	No	No Information
Yes	Yes	No Information	No Information
Yes	Yes	Yes	No Information
Yes	Yes	Yes	No Information
Yes	Yes	Yes	No Information
Yes	No	Yes	No Information
Yes	Probably No	No	No Information
Yes	No	No	No Information

For peer review only

Were relevant model performance measures evaluated appropriately?	Were model overfitting, underfitting, and optimism in model performance accounted for?	Do predictors and their assigned weights in the final model correspond to the results from the reported multivariable analysis?	Risk of Bias in analysis
Probably No	No Information	Probably Yes	High
Probably No	No Information	Probably Yes	High
Probably No	No Information	Probably Yes	High
Yes	No Information	Yes	High
No	No Information	Probably No	High
No	No Information	Yes	High
No	No Information	Yes	High
Yes	Yes	Yes	High
Yes	Yes	Yes	High
Yes	Yes	Yes	High
Yes	No Information	Yes	High
Yes	Yes	Yes	High
Probably No	No Information	Probably No	High
No	Yes	No Information	High
No Information	Yes	No Information	Unclear
No	No Information	Yes	Low
No	No Information	Yes	Low
No	No Information	Yes	Low
No Information	No Information	Yes	High
No	No Information	No	High
No	No Information	No	High

For peer review only

Reporting checklist for systematic review and meta-analysis.

Based on the PRISMA guidelines.

Instructions to authors

Complete this checklist by entering the page numbers from your manuscript where readers will find each of the items listed below.

Your article may not currently address all the items on the checklist. Please modify your text to include the missing information. If you are certain that an item does not apply, please write "n/a" and provide a short explanation.

Upload your completed checklist as an extra file when you submit to a journal.

In your methods section, say that you used the PRISMAreporting guidelines, and cite them as:

Moher D, Liberati A, Tetzlaff J, Altman DG, The PRISMA Group. Preferred Reporting Items for Systematic Reviews and Meta-Analyses: The PRISMA Statement

		Reporting Item	Page Number
**Title**			
	#1	Identify the report as a systematic review, meta-analysis, or both.	1
**Abstract**			

For peer review only

Structured summary	#2	Provide a structured summary including, as applicable: background; objectives; data sources; study eligibility criteria, participants, and interventions; study appraisal and synthesis methods; results; limitations; conclusions and implications of key findings; systematic review registration number	2
**Introduction**			
Rationale	#3	Describe the rationale for the review in the context of what is already known.	3
Objectives	#4	Provide an explicit statement of questions being addressed with reference to participants, interventions, comparisons, outcomes, and study design (PICOS).	3
**Methods**			
Protocol and registration	#5	Indicate if a review protocol exists, if and where it can be accessed (e.g., Web address) and, if available, provide registration information including the registration number.	4
Eligibility criteria	#6	Specify study characteristics (e.g., PICOS, length of follow-up) and report characteristics (e.g., years considered, language, publication status) used as criteria for eligibility, giving rational	4
Information sources	#7	Describe all information sources in the search (e.g., databases with dates of coverage, contact with study authors to identify additional studies) and date last searched.	N/A
Search	#8	Present full electronic search strategy for at least one database, including any limits used, such that it could be repeated.	5

For peer review only

Study selection	#9	State the process for selecting studies (i.e., for screening, for determining eligibility, for inclusion in the systematic review, and, if applicable, for inclusion in the meta-analysis).	6
Data collection process	#10	Describe the method of data extraction from reports (e.g., piloted forms, independently by two reviewers) and any processes for obtaining and confirming data from investigators.	6
Data items	#11	List and define all variables for which data were sought (e.g., PICOS, funding sources), and any assumptions and simplifications made.	6
Risk of bias in individual studies	#12	Describe methods used for assessing risk of bias in individual studies (including specification of whether this was done at the study or outcome level, or both), and how this information is to be used in any data synthesis.	7
Summary measures	#13	State the principal summary measures (e.g., risk ratio, difference in means).	N/A
Planned methods of analyis	#14	Describe the methods of handling data and combining results of studies, if done, including measures of consistency (e.g., I2) for each meta-analysis.	N/A
Risk of bias across studies	#15	Specify any assessment of risk of bias that may affect the cumulative evidence (e.g., publication bias, selective reporting within studies).	7
Additional analyses	#16	Describe methods of additional analyses (e.g., sensitivity or subgroup analyses, meta-regression), if done, indicating which	N/A

For peer review only

		were pre-specified.	
**Results**			
Study selection	#17	Give numbers of studies screened, assessed for eligibility, and included in the review, with reasons for exclusions at each stage, ideally with a flow diagram.	8
Study characteristics	#18	For each study, present characteristics for which data were extracted (e.g., study size, PICOS, follow-up period) and provide the citation.	8
Risk of bias within studies	#19	Present data on risk of bias of each study and, if available, any outcome-level assessment (see Item 12).	11
Results of individual studies	#20	For all outcomes considered (benefits and harms), present, for each study: (a) simple summary data for each intervention group and (b) effect estimates and confidence intervals, ideally with a forest plot.	N/A
Synthesis of results	#21	Present the main results of the review. If meta-analyses are done, include for each, confidence intervals and measures of consistency.	N/A
Risk of bias across studies	#22	Present results of any assessment of risk of bias across studies (see Item 15).	11
Additional analysis	#23	Give results of additional analyses, if done (e.g., sensitivity or subgroup analyses, meta-regression [see Item 16]).	N/A
**Discussion**			

For peer review only

Summary of Evidence	#24	Summarize the main findings, including the strength of evidence for each main outcome; consider their relevance to key groups (e.g., health care providers, users, and policy makers	12
Limitations	#25	Discuss limitations at study and outcome level (e.g., risk of bias), and at review level (e.g., incomplete retrieval of identified research, reporting bias).	15
Conclusions	#26	Provide a general interpretation of the results in the context of other evidence, and implications for future research.	16

Funding

Funding	#27	Describe sources of funding or other support (e.g., supply of data) for the systematic review; role of funders for the systematic review.	17

The PRISMA checklist is distributed under the terms of the Creative Commons Attribution License CC-BY. This checklist was completed on 15. October 2019 using https://www.goodreports.org/, a tool made by the EQUATOR Network in collaboration with Penelope.ai

For peer review only

BMJ Open

Prognostic models for predicting in-hospital paediatric mortality in resource-limited countries: a systematic review

Journal:	*BMJ Open*
Manuscript ID	bmjopen-2019-035045.R1
Article Type:	Original research
Date Submitted by the Author:	01-Jul-2020
Complete List of Authors:	ogero, morris; KEMRI-Wellcome Trust Research Programme; University of Nairobi College of Biological and Physical Sciences, School of Mathematics<br>Sarguta, Rachel ; University of Nairobi College of Biological and Physical Sciences, School of Mathematics<br>Malla, Lucas; KEMRI-Wellcome Trust Research Programme Nairobi<br>Aluvaala, Jalemba ; KEMRI-Wellcome Trust Research Programme Nairobi, Health Services Unit<br>Agweyu, Ambrose; KEMRI-Wellcome Trust Research Programme Nairobi<br>English, Mike; Oxford University, Nuffield Department of Medicine and Department of Paediatrics; KEMRI-Wellcome Trust Research Programme Nairobi<br>Onyango, Nelson ; University of Nairobi College of Biological and Physical Sciences, School of Mathematics<br>Akech, Samuel; KEMRI-Wellcome Trust Research Programme Nairobi
**Primary Subject Heading**:	Paediatrics
Secondary Subject Heading:	Research methods, Paediatrics, Health services research
Keywords:	STATISTICS & RESEARCH METHODS, PAEDIATRICS, Paediatric intensive & critical care < PAEDIATRICS

SCHOLARONE™
Manuscripts

I, the Submitting Author has the right to grant and does grant on behalf of all authors of the Work (as defined in the below author licence), an exclusive licence and/or a non-exclusive licence for contributions from authors who are: i) UK Crown employees; ii) where BMJ has agreed a CC-BY licence shall apply, and/or iii) in accordance with the terms applicable for US Federal Government officers or employees acting as part of their official duties; on a worldwide, perpetual, irrevocable, royalty-free basis to BMJ Publishing Group Ltd ("BMJ") its licensees and where the relevant Journal is co-owned by BMJ to the co-owners of the Journal, to publish the Work in this journal and any other BMJ products and to exploit all rights, as set out in our licence.

The Submitting Author accepts and understands that any supply made under these terms is made by BMJ to the Submitting Author unless you are acting as an employee on behalf of your employer or a postgraduate student of an affiliated institution which is paying any applicable article publishing charge ("APC") for Open Access articles. Where the Submitting Author wishes to make the Work available on an Open Access basis (and intends to pay the relevant APC), the terms of reuse of such Open Access shall be governed by a Creative Commons licence – details of these licences and which Creative Commons licence will apply to this Work are set out in our licence referred to above.

Other than as permitted in any relevant BMJ Author's Self Archiving Policies, I confirm this Work has not been accepted for publication elsewhere, is not being considered for publication elsewhere and does not duplicate material already published. I confirm all authors consent to publication of this Work and authorise the granting of this licence.

For peer review only

Prognostic models for predicting in-hospital paediatric mortality in resource-limited countries: a systematic review

Morris Ogero[1, 2], Rachel Sarguta[2], Lucas Malla[1], Jalemba Aluvaala[1], Ambrose Agweyu[1], Mike English[1, 3], Nelson Onyango[2], Samuel Akech[1]

[1]Kenya Medical Research Institute (KEMRI)-Wellcome Trust Research Programme, Nairobi, Kenya; [2]School of Mathematics, University of Nairobi, Kenya; and [3]Nuffield Department of Medicine, University of Oxford, UK.

Correspondence: Morris Ogero,

KEMRI / Wellcome Trust Research Programme**,**

P.O Box 43640-00100 Nairobi, Kenya.

mogero@kemri-wellcome.org

For peer review only

Abstract

Objectives: To identify and appraise the methodological rigor of multivariable prognostic models predicting in-hospital paediatric mortality in low- and middle-income countries (LMIC).

Design: Systematic review of peer-reviewed journals.

Data sources: MEDLINE, CINAHL, Google Scholar, and Web of Science electronic databases since inception to August 2019.

Eligibility criteria: We included model development studies predicting in-hospital paediatric mortality in LMIC.

Data extraction and synthesis: This systematic review followed the CHARMS (Checklist for critical Appraisal and data extraction for systematic Reviews of prediction Modelling Studies) framework. The risk of bias assessment was conducted using PROBAST (Prediction model Risk of Bias Assessment Tool). No quantitative summary was conducted due to substantial heterogeneity that was observed after assessing the studies included.

Results: Our search strategy identified a total of 4054 unique articles. Among these, 3545 articles were excluded after review of titles and abstracts as they covered non-relevant topics. Full texts of 509 articles were screened for eligibility, of which 15 studies reporting 21 models met the eligibility criteria. Based on the PROBAST tool, risk of bias was assessed in four domains; participant, predictors, outcome, and analyses. The domain of statistical analyses was the main area of concern where none of the included models was judged to be of low risk of bias.

Conclusion: This review identified 21 models predicting in-hospital paediatric mortality in LMIC. However, most reports characterising these models are of poor quality when judged against recent reporting standards due to a high risk of bias. Future studies should adhere to

For peer review only - http://bmjopen.bmj.com/site/about/guidelines.xhtml

standardized methodological criteria and progress from identifying new risk scores to validating or adapting existing scores.

Review registration number: CRD42018088599

Article summary

Strengths and limitations of this study

- This is the first systematic review on methodological quality of prognostic models predicting in-hospital paediatric mortality in resource-limited settings.
- We used a robust search strategy with no language restriction yielding a large number of potentially eligible studies, hence it is unlikely that any potentially eligible study was not included.
- No meta-analysis was conducted due to substantial heterogeneity in the included studies.
- We relied on what was reported to determine the risk of bias of the models.
- Google Translate was used to translate one study from French to English. It is therefore possible that some statistical terminologies were not rendered correctly.

For peer review only

For peer review only - http://bmjopen.bmj.com/site/about/guidelines.xhtml

Introduction

Over recent decades, there has been considerable progress in improving child survival[1] but child mortality remains high in sub-Saharan Africa relative to the rest of the world.[2] Paediatric deaths in hospitalized children mostly occur soon after admission,[3] and are caused by common conditions such as malaria, pneumonia, and diarrhoeal diseases among others, which are readily treatable by cost-effective interventions.[3-5] In low- and middle-income countries (LMIC), clinicians often use a set of clinical signs as recommended in the guidelines by World Health Organization (WHO) to identify patients at risk of deterioration while making decisions on appropriate treatment.[6] Clinical criteria recommended by WHO were developed following expert recommendations based on review of evidence from studies reporting risk factors for mortality. Prognostic models, which use statistical equations to predict patients' risk based on the combination of prognostic factors, may improve clinicians' ability to identify high-risk patients and thus improve outcomes.[7]

Various clinical prediction models for hospitalised paediatric patients have been developed over the last 3 decades,[8] however, there are doubts whether appropriate methodology has been used in their development.[9] Notably, none are currently recommended for use in existing paediatric clinical practice guidelines in LMIC and systematic reviews of the methodology used in their development have been strongly recommended.[10] This systematic review addresses this need and aims at identifying and summarizing existing studies reporting prognostic models for predicting in-hospital paediatric mortality in LMIC. Specifically, the research summarises the evidence from the published studies and appraises the methodological rigor of each existing model.

For peer review only - http://bmjopen.bmj.com/site/about/guidelines.xhtml

Methods

Protocol and registration

As recommended, a research protocol for this systematic review was published in a peer-reviewed journal,[11] and we also registered at PROSPERO (International Prospective Register of Systematic Reviews) (CRD42018088599).[12] This work is reported as per guidelines by the PRISMA (Preferred Reporting Items for Systematic Reviews and Meta-Analyses).[13]

Eligibility criteria

Reports were eligible for inclusion if they met the following criteria:

1. Study design: peer-reviewed studies whose design was either a randomized controlled trial, cohort (prospective or retrospective), cross-sectional, or case-control observational study.
2. Outcome: studies fitting models predicting all-cause in-hospital mortality in a general paediatric ward were included. Studies predicting post-discharge mortality, trauma or operative mortality were excluded.
3. Target population and setting: studies on children aged over 1-month old admitted in general paediatric wards within LMIC as defined by the World Bank[14] were included. Studies whose predictive models were targeting patients in intensive care unit (ICU) or high dependency unit (HDU) were excluded because these facilities are largely unavailable in low-resource settings. We also excluded studies whose predictive models targeted uncommon conditions in children e.g., chronic kidney disease, cancer, diabetes.

For peer review only - http://bmjopen.bmj.com/site/about/guidelines.xhtml

However, if a study focused on one of the common childhood illnesses such as malaria, pneumonia, meningitis, anaemia, and diarrhoea/dehydration[3], then it was included.

4. Type of studies: we included studies whose main objective was deriving a predictive model(s) or scoring system(s). We excluded commentaries, editorials, expert views, conference proceedings, case reports, case-series, reviews and explanatory studies that mainly generate hypothesis[15].
5. Models: studies that reported multivariable model with at least 2 variables/predictors were included.
6. Full text and language: We excluded studies that were not available in full text. Non-English language studies were translated using Google Translate. Hence no language restriction was made.

Search strategy

As recommended by CHARMS (Checklist for critical Appraisal and data extraction for systematic Reviews of prediction Modelling Studies) checklist[16], we came up with seven key items (supplementary file 1 Table 1) applicable to our study that guided the framing of the search strategy, review aim and eligibility criteria.

We used Medical Subject Headlines (MeSH) where appropriate and keywords to identify articles with prognostic models relevant for this review. A search of articles was conducted in MEDLINE, Google Scholar, and CINAHL (via EbscoHost) since inception to August 2019. We also performed a search in Web of Science to identify additional reports that cited the identified studies. Reference lists of all identified articles were searched manually to identify other potentially eligible studies.

For peer review only - http://bmjopen.bmj.com/site/about/guidelines.xhtml

For peer review only

We manually searched reference lists of all relevant articles to identify additional eligible studies. Final search results were collated in EndNoteX7™ bibliography tool. Detailed search terms and strategy are provided in supplementary file 1 Table 2.

Screening of articles

Prior to screening titles and abstracts, 2 reviewers (MO and LM) standardized the approach to be used in the process of screening. We used a sample of 30 search results to train and familiarize reviewers with the screening process. Titles and abstracts of the studies were screened by the two reviewers. Discrepancies were resolved via discussion and, when necessary, a final decision was adjudicated by a third reviewer (JA).

Data Extraction

Data were extracted from relevant articles in accordance with the CHARMS. From each study included, data were extracted on participant enrolment, study design, study population characteristics, location, sample size, number and selection of predictors, study dates, handling of continuous predictors, missing data, method of modelling (e.g. logistic regression, survival), verification of model assumptions, internal validation methods (e.g. random split of data, resampling techniques); model presentation (e.g. nomogram, score chart, or regression formula with coefficients); and model performance metrics including discrimination -area under the receiver operating characteristic curve (AUC) with 95% confidence interval (CI); calibration; classification measures such as sensitivity, specificity, positive, and negative predictive values. We further investigated from literature to determine if included models have been externally validated elsewhere. For articles that described development of multiple prognostic models, we treated each model separately whenever the predictor-outcome association produced different

For peer review only - http://bmjopen.bmj.com/site/about/guidelines.xhtml

model estimates. For each study, extracted data elements were compared between two reviewers (MO & LM), and any disagreements were resolved by discussion with the third reviewer (JA). No quantitative summary was conducted due to substantial heterogeneity that was observed after assessing studies included.

Assessment of methodological quality

The *risk of bias* (shortcomings in the predictive models that might lead to unreliable predictions) of the included studies was assessed based on the Prediction study Risk Of Bias Assessment Tool (PROBAST).[17 18] We assessed the *risk of bias* (RoB) for each model in four domains: participant selection (e.g. study design), predictors (e.g. assessment of candidate and final model predictors), outcome, and analysis (e.g. handling of missing data, the handling of continuous predictors) see supplementary file 1 Table 3. For each domain, signalling questions had five possible answers: yes; probably yes; probably no; no; and no information. Any positive answer (yes, or probably yes) suggests low RoB. Each domain had three possible outcomes: low; high; or unclear RoB. Using these domain outcomes, we came up with an overall judgement of RoB for each model. As recommended by PROBAST, if a prediction model was judged as low on all four domains, we assigned it an overall judgment of "low RoB". If a model was rated as high at least in one domain, we judged it as having "high RoB". If at least one domain of the model was rated as unclear and the rest of the domains rated as low, it was judged as having "unclear RoB".

Patient and public involvement

No patient or public involvement.

For peer review only - http://bmjopen.bmj.com/site/about/guidelines.xhtml

Results

Characteristics of the included studies

Our search strategy identified a total of 4054 unique articles, 3545 articles were excluded after review of titles and abstracts as they reported non-relevant topics. Full texts of 509 articles were screened for eligibility, of which 15 primary studies reporting 21 developed models met the eligibility criteria (Figure 1). The eligible studies analysed data for patients who were below 15 years of age with median mortality being 6.7% (range 1.2% to 43.9%).[19 20] While majority of the models were developed for general cases in paediatric wards (n=9), some were tailored for specific paediatric groups defined by common diagnoses such as febrile illness (n=1),[21] malaria (n=2), [22 23] pneumonia (n=4),[19 24-26] malnutrition (n=2) [27 28] and other infectious diseases (n=3) (see supplementary file 2).

Most of the included studies have been published post year 2000 (n = 20) except for one study[27] published in 1996. The latest data used in the models under review were from 2016 to 2017 by Rosman *et al.*[29] and the oldest data were utilized by Draimax *et al.*[27] from 1986 to 1988.

Five reports of the 15 included studies utilized data from at least two hospitals of which 3 studies[21 22 26] were conducted in multiple countries including sub-Saharan Africa and Asian countries (Figure 2). Of the reviewed studies, most of the information we were abstracting were either not reported or were partially reported, an indication of non-adherence to the TRIPOD (Transparent Reporting of a Multivariable Prognostic Model for Individual Prognosis or Diagnosis) guidelines.[30 31]

For peer review only - http://bmjopen.bmj.com/site/about/guidelines.xhtml

Summary of issues in methodology of the reviewed models

Candidate predictors

There were 61 distinct predictors used in the final reported models (a median of 7 predictors in any one model). Initial selection of the candidate predictors was mostly based on univariable analysis except for three studies[21 25 27] where the selection was based on literature reviews or clinical relevance. Backward stepwise selection method was used in 6 models in a multivariable analysis to determine final model predictors. Commonly included predictors in the final models included altered consciousness, malnutrition indicators, vital signs, and signs of respiratory distress (see Figure 3). Some models included predictors that were either not easy to obtain or required laboratory techniques. Of the 13 models including continuous predictors, 8 models categorized continuous predictors where a continuous scale would have been possible. Two out of 13 models applied other techniques such as fractional polynomial[21] and restricted cubic splines[28] to determine the suitable functional form of the continuous predictors (see supplementary file 2).

Sample size, events per variable (EPV) and missing data

Sample size ranged from 168[29] to 50249[32] with a median of 1307. The median EPV was 21(IQR 8.3 – 32.5) of which 7 models had less than 10 EPVs, suggestive of insufficient sample sizes which is prone to over-fitting. For instance, 60 deaths were reported in the dataset used to develop *PEDIA-Immediate* score in the study by Berkley *et al*. In reference to the rule that a study developing a predictive model should have a minimum of 10 events for each independent predictor in a prognostic model,[33] a model with, at the most, 6 predictors should have been considered but 10 predictors were considered instead hence making EPV 6.

For peer review only - http://bmjopen.bmj.com/site/about/guidelines.xhtml

Proportions of missing data was not always reported. Handling of missing data varied across the reviewed studies as follows: 6 models did not report handling of missing data; 8 used complete case analysis; 4 used multiple imputations by chained equations; and one study[28] used single imputation.

Model development

Majority of the studies applied logistic regression, one study[21] used Cox regression, one study[20] used Spiegelhalter/Knill-Jones method and another study[23] used a machine learning technique (classification and regression trees (CRT)) in model development. Verification of model assumptions was not reported in most of the studies. For instance, George *et al*[21] despite utilizing Cox regression to develop their model, did not report the verification of proportional hazard assumption nor explore the possibility of competing risks as recommended[34]. Other regression assumptions e.g. multicollinearity was equally not reported. However, since backward elimination method disregards redundant variables, we inferred the satisfaction of multicollinearity assumption if this method was applied.[35] Five studies developed models using data from different countries/centres but none of them clustered their analysis by source of data in a multilevel model to account for heterogeneity. Ignoring clustering leads to a biased predictor effect.[36]

Model performance evaluation & presentation

Performance measures (both calibration and discrimination) were poorly reported in most of the studies and in most cases (n=20) AUC for discrimination was reported. Performance of the derived models was evaluated in 12 models using either split-sample, resampling methods, or separate datasets. Except for the model derived by George *et al,*[21] all other models did not report both apparent discrimination (without any adjustment for optimism) and optimism-corrected

For peer review only - http://bmjopen.bmj.com/site/about/guidelines.xhtml

discrimination measures. Despite inadequate reporting of the models' performance, 16 models reported AUCs ≥ 0.80, an indication of promising models. Apart from the following exceptions; *Lambarene Organ Dysfunction* (LOD) score,[22] *Paediatric Early Death Index for Africa* (PEDIA) score,[20] *Signs of Inflammation in Children that Kill* (SICK) score,[37] *Respiratory Index of Severity in Children*(RISC) score,[19] and *Modified Respiratory Index of Severity in Children* (mRISC) score,[24] other scores have not been externally validated (by independent investigators using diverse populations). Only 2 studies[25 38] developing 4 models provided a full model formula (both coefficients and intercept/baseline function) in their results as recommended.[30 31] While most of the models (n=17) were presented as simplified integer scores, only a few were assigned weights according to the regression coefficients.

Risk of bias (RoB) assessment

Based on the PROBAST tool, RoB was assessed in four domains; participants, predictors, outcome, and analyses. Figure 4 summarizes the RoB assessment across all models included in this review where the domain of outcome was deemed to be of low RoB in all models. The domain of statistical analyses was the main area of concern where 19 out of 21 models did not report comprehensive details of model development as expected to warrant a proper risk of bias assessment using the 9 signalling questions under analyses domain. As a result, these models were rated to be of unclear RoB under the domain of analyses (see Figure 5). Details on how models were scored against each of the PROBAST criterion (20 signalling questions) across four domains are provided in the supplementary file 3. In the overall judgement of RoB, 9 out of 21 models were judged to be of high risk of bias because at least one out of four domains in these models were rated as high RoB. The remaining models (12/21) were judged to be of unclear RoB on account of being rated low and unclear RoB in the domains. Two models[24 26] were judged to

For peer review only - http://bmjopen.bmj.com/site/about/guidelines.xhtml

be of high RoB because at least one of the domains was rated high RoB. No model was rated low RoB in all four domains.

Discussion

Summary of key findings

We conducted a systematic review to identify published scores predicting in-hospital mortality for paediatrics in resource-limited countries. Fifteen studies that described the development of 21 prognostic models were identified. We describe characteristics of these studies as well as the methodological quality of the included models by using agreed recent guidelines applicable to predictive models. We have identified several important quality deficiencies such as inadequate reporting and other methodological concerns, including poor handling of missing data, automated selection of predictors, categorization of continuous predictors, inadequate EPV and the poor presentation of the proposed model for use. As a result, no model was found to be of good methodological quality and consequently judged to be potentially high or unclear risk of bias in predictions (Figure 5).

Our findings suggest that predictive models fail to meet recently agreed methodological criteria in various ways. Firstly, in this review we observed that univariable analysis was routinely used in 18 out of 21 models in the selection of candidate predictors to be used in a multivariable analysis. This strategy tends to leave out possibly important prognostic factors which might be insignificant in a univariable analysis but turn out to be significant when combined with other predictors.[30 31] *A priori* selection of predictors using expert opinion, clinical intuition or literature is recommended for this purpose,[39 40] however only three studies in this review employed this approach.[21 25 27]

For peer review only - http://bmjopen.bmj.com/site/about/guidelines.xhtml

Small sample sizes in model development can lead to poor predictive performance, over-fitting, and biased effect estimates. Prognostic models must have a minimum of 10 events per candidate predictor, as this is the accepted norm[41-43] and underpowered models arising from inadequate events per variable (EPV) increases the possibility of spurious results.[33] In this review, 7/21 models had inadequate sample sizes (EPV<10) and there was no information on whether bootstrapping, which serves to reduce overfitting was used in these models.[44]

Just like most of the epidemiological studies, missing data is a common problem which is solved using multiple imputation or other appropriate approaches, but this was rarely the case in the model development studies under this review. For instance, 8/21 models used complete case analysis (CCA), 4/21 used multiple imputation under the assumption of missing at random (MAR), and 6/21models did not report how missing data were handled and therefore we assumed CCA was used. Following Harrell's guidelines,[45] CCA should only be used if the percentage of missingness is < 5% but the appropriateness of the CCA approach could not be ascertained as most of the included studies failed to report the proportion of missing data per variable. Inappropriate use of CCA results in use of only a small subset of the data which cannot be regarded as a random sample from the target population unless data are missing completely at random(MCAR),[46] a mechanism which is rare in practice.[47] Consequently, there are concerns about possible loss of precision in inferences and the potential biases of the estimated parameters[48] in the models employing CCA. While Multiple Imputation by Chained Equations (MICE) is the principled method of handling missing data, implementing this method when the data are not MAR could result in biased model estimates.[49] As a result, sensitivity analyses of the resultant imputations is recommended to investigate the departure from MAR assumption.[50] However, this was not the case in the studies that performed imputations on their data. Finally,

For peer review only - http://bmjopen.bmj.com/site/about/guidelines.xhtml

handling of continuous predictors was also a concern in this review. Of the 13 models including continuous predictors, 8 models[19 20 24-27 37 51] categorized continuous predictors where a continuous scale would have been possible. While this approach is intuitive to most researchers, its simplicity comes at a considerable cost of predictive performance.[52] The resulting prognostic models have been shown to have poor predictive accuracy because of the loss of statistical power and information. It is recommended that the nature of continuous data should be retained or be handled by using techniques e.g. regression splines, flexible parametrizations such as fractional polynomial, or apply non-parametric techniques such as locally estimated scatterplot smoothing (LOESS) functions.[52 53] In this review, appropriate methods of transforming continuous data was done by only 2 studies[21 28] which applied fractional polynomial and restricted cubic splines. Sixteen models attained the discrimination metric of above 80%, an indicator of promising models. However, given that the median mortality of the included studies was 6.7%, the performance reported should be interpreted with caution on account of heavily imbalanced data as a result of the rare nature of the outcome of interest. For instance, in a study with a mortality rate of 5%, a model predicting no deaths could easily attain 95% accuracy which could be potentially misleading[34 54]. Therefore, authors should report additional measures of model performance such as model sensitivity, specificity, accuracy, positive and negative predictive values for models to be contextualized appropriately.

Comparison with Other Studies

Methods used to assess quality measures of the included models in the current study have been applied previously to critically evaluate the quality of predictive models in other specialties.[55-57] Just like the findings of this review, other previous reviews [9 58-60] describing the development of prognostic models highlighted many flaws including inappropriate statistical analyses, poor

For peer review only - http://bmjopen.bmj.com/site/about/guidelines.xhtml

reporting of important clinical and methodological information needed for validation of the model, and lack of external validations. Detailed and transparent reporting of the methods used in model development is one of the core principles of integrity in research because this is the only way the research community is able to judge the reliability of research findings, and the assessment of risk of bias.[61] Incomplete reporting of clinical models limits future studies on prognostic research from building on the information of already existing models. This has been marked as an important source of wasted research efforts.[62] For example, external validation of prognostic models requires a full model formula to enable direct estimation of survival probabilities.[31] However, this was presented in only 4 models . Five models [19 20 22 24 37] that were reported to have undergone external validation did not report full model formula as required. It is therefore not clear whether authors of these external validation studies applied model coefficients to the external datasets, or they estimated new model coefficients (essentially model redevelopment). Thus, this review highlights the need for researchers to adhere to the TRIPOD guidelines that were created to help authors of prognostic models write complete and transparent reports. Of note, the quality of clinical predictive models does not appear to have improved over time as previous reviews from 1996,[63] 1997,[64] 2001,[65] 2005,[66] 2011,[8] 2012,[67] 2016,[68 69] 2017, [70] to 2019[71] have consistently identified suboptimal methodologies in the development of the predictive models especially in the domain of analysis. Poorly derived models may result in overoptimistic results and misleading performances. Presumably there are reasons why many prognostic models are of poor quality, including pressure to publish new predictive model regardless of the clinical value of the resultant model[72], and inadequate biostatistical support to investigators. As observed by one of the reviewers of this study, some of the issues identified in this review such as absence of the details on the model development process can be corrected

For peer review only - http://bmjopen.bmj.com/site/about/guidelines.xhtml

during the review and the editorial process by the journals publishing the work. There is therefore a role for editorial process for promoting best practices and recommendations of developing predictive models stated in the TRIPOD statement and ensuring compliance by authors as part of checklist for submission.

Implications of this study

Prognostic model development pipeline include development, validation (internal and external), impact assessment and implementation. Most of the included models are still in the first step of the pipeline. This suggests that researchers focus more on deriving new models, often using similar prognostic factors, rather than validating and improving existing prognostic models. This leaves healthcare policy makers with doubts as to which model to recommend in their setting. It is now time to move the prognostic research to the next step (external validation). Large patient-level datasets such as that of the Clinical Information Network (CIN)[3] which has been collected over time from a number of referral hospitals now exist in Kenya and it has been used to answer a number of salient clinical questions relevant across a range of resource-limited setting[73-75]. Future studies on prognostic research should leverage such datasets to externally validate competing models identified in this review for comparative performances as recommended by Collins *et al,*[76] and if necessary, predictive performance of such models should be improved by addition of new prognostic factors. We also noted that most of the included models simplified the original predictor coefficients by rounding them to a nearest integer. This practice has an implication on model performance during external validation due to loss in predictive accuracy arising from rounding coefficients to nearest integers.[47]

We now provide guidance on methodological concerns about the candidate predictors as noted in this review. While considering potential candidate predictors to include in the prediction model,

For peer review only - http://bmjopen.bmj.com/site/about/guidelines.xhtml

researchers should focus on the predictors that will be available at the time the prediction is made. We acknowledge that some predictors obtained from invasive procedures e.g. C-reactive protein, blood gas analyses, blood or cerebrospinal fluid culture, etc might have a higher predictive value for mortality compared to predictors derived from subjective clinical assessments, however in resource-limited settings results of such laboratory tests typically take days to be reported or resources might not available to perform such tests in many hospitals. Consequently, models utilising such variables might not be useful to clinicians to make a decision at typical emergency departments in LMIC. Screening of model candidate predictors based on the bivariate associations whereby predictors are selected if they meet some *p*-value threshold (commonly 0.05) have been strongly discouraged previously[77 78]. Categorising continuous model predictors is a common practice by researchers however this practice discards a lot of information and its assumptions are rarely clinically plausible.[34] Finally, there is a risk of overfitting if the model includes more predictors than the dataset can support. The ratio of the number of outcomes to the number of predictors (events per variable) have been discussed extensively in methodological papers elsewhere[79 80] and it has been recommended that ratio of the EPV should be at least 10.

Strengths and limitations

To our knowledge, this is the first review identifying models predicting in-hospital paediatric mortality in resource-limited settings. Our robust search strategy yielded a large number of potentially eligible studies, hence it is unlikely that any potentially eligible study was not included. The quality of included models was assessed based on recent reporting standards and

For peer review only - http://bmjopen.bmj.com/site/about/guidelines.xhtml

applied to the identified studies. For instance, if no mention was made of internal validation or even verification of the model assumptions, it could not be determined whether these crucial steps of model development were carried out or not. Thus, models that could have been otherwise rated as low risk of bias were rated as either unclear or high risk of bias in each domain. The PROBAST's analysis domain has most (9 out of 20) of the signalling questions and any given model in this domain had much higher chance to be defined as high risk as long as there was one negative (no or probably no) answer. This strict criterion led to all models being classified as either unclear or high risk of bias and therefore metanalysis was not performed. We acknowledge that if we somewhat relaxed this decision rule, our conclusion could change. Despite this, we still hold that authors should adhere to guidelines of transparent and complete reporting of any proposed prognostic model to facilitate its external validation and subsequent application in practice. Finally, we used Google Translate to interpret a study by Bitwe et al[51] from French to English. It is possible that some statistical terminologies were not rendered correctly, or some model characteristics were lost in translation.

Conclusion

Rigorously developed and robustly validated promising predictive models have the potential for improving child survival in resource-limited countries. This review identified models predicting in-hospital mortality for paediatrics. However, none of them is of good quality. Our research highlights the need to improve on the identified quality deficiencies when developing prognostic models in the future by adhering to existing generally accepted standardized methodological criteria. Majority of the derived models have not been externally validated as required. Inadequate reporting observed in the included models hinders rigorous external validation by other researchers in addition to undermining their application in practice. Rather than developing

For peer review only - http://bmjopen.bmj.com/site/about/guidelines.xhtml

new prognostic models, researchers should carry out comprehensive joint external validation of the identified models using large datasets ideally collected over extended time periods and different locations. This will allow head-to-head comparisons and adaptation of the competing models, if necessary, to ascertain their generalizability.

Funding

Funds from The Wellcome Trust (#207522) awarded to ME as a Senior Fellowship together with additional funds from a Wellcome Trust core grant awarded to the KEMRI-Wellcome Trust Research Programme (#092654 and #203077) supported this work. SA was supported by the Initiative to Develop African Research Leaders (IDeAL) Wellcome Trust award (#107769). The funders had no role in drafting or submitting this manuscript.

Author Statement

The roles of the contributors were as follows: ME, SA, and AA conceptualized the study. MO, LM and JA conducted electronic searches to identify eligible models and did analyses. MO drafted the initial manuscript with SA, NO, RS, AA, and ME contributed to its development. All authors read and approved the final manuscript.

Competing interests

The authors declare no conflicts of interest.

Data sharing statement

No additional data are available.

For peer review only

For peer review only - http://bmjopen.bmj.com/site/about/guidelines.xhtml

References

1. World Health Organization. Child mortality rates plunge by more than half since 1990 but global MDG target missed by wide margin: WHO; 2015 [Available from: http://www.who.int/mediacentre/news/releases/2015/child-mortality-report/en/ accessed 20 January 2018.
2. Lucia Hug DS, Danzhen You. Levels and Trends in Child Mortality: UNICEF; 2019 [Available from: https://data.unicef.org/resources/levels-and-trends-in-child-mortality/ accessed August 2019.
3. Ayieko P, Ogero M, Makone B, et al. Characteristics of admissions and variations in the use of basic investigations, treatments and outcomes in Kenyan hospitals within a new Clinical Information Network. *Archives of disease in childhood* 2015:archdischild-2015-309269.
4. World Health Organization. Serious childhood problems in countries with limited resources. Geneva2004.
5. World Health Organization. Children: reducing mortality: WHO; 2017 [Available from: http://www.who.int/mediacentre/factsheets/fs178/en/ accessed 20 January 2018.
6. World Health Organization. Handbook IMCI: integrated management of childhood illness. Geneva2005.
7. Vogenberg FR. Predictive and prognostic models: implications for healthcare decision-making in a modern recession. *American health & drug benefits* 2009;2(6):218.
8. Maguire JL, Kulik DM, Laupacis A, et al. Clinical prediction rules for children: a systematic review. *Pediatrics* 2011;128(3):e666-e77. doi: 10.1542/peds.2011-0043
9. Bouwmeester W, Zuithoff NP, Mallett S, et al. Reporting and methods in clinical prediction research: a systematic review. *PLoS medicine* 2012;9(5):e1001221.
10. Altman DG. Systematic reviews in health care: Systematic reviews of evaluations of prognostic variables. *BMJ: British Medical Journal* 2001;323(7306):224.
11. Ogero M, Sarguta R, Malla L, et al. Methodological rigor of prognostic models for predicting in-hospital paediatric mortality in low-and middle-income countries: a systematic review protocol. *Wellcome Open Research* 2020;5(106):106.
12. Ogero M, Malla L, Agweyu A, et al. Prognostic models predicting in-hospital mortality of paediatrics in resource-limited countries: systematic review. 2018
13. Moher D, Liberati A, Tetzlaff J, et al. Preferred reporting items for systematic reviews and meta-analyses: the PRISMA statement. *PLoS medicine* 2009;6(7):e1000097.
14. WORLD BANK. World Bank Country and Lending Groups: WOLD BANK; 2019 [Available from: https://datahelpdesk.worldbank.org/knowledgebase/articles/906519-world-bank-country-and-lending-groups accessed September 2019.
15. Shmueli G. To explain or to predict? *Statistical science* 2010:289-310.
16. Moons KG, de Groot JA, Bouwmeester W, et al. Critical appraisal and data extraction for systematic reviews of prediction modelling studies: the CHARMS checklist. *PLoS medicine* 2014;11(10)
17. Wolff RF, Moons KG, Riley RD, et al. PROBAST: a tool to assess the risk of bias and applicability of prediction model studies. *Annals of internal medicine* 2019;170(1):51-58.
18. Moons KG, Wolff RF, Riley RD, et al. PROBAST: a tool to assess risk of bias and applicability of prediction model studies: explanation and elaboration. *Annals of internal medicine* 2019;170(1):W1-W33.
19. Reed C, Madhi SA, Klugman KP, et al. Development of the Respiratory Index of Severity in Children (RISC) score among young children with respiratory infections in South Africa. *Plos One* 2012;7(1):e27793-e93. doi: 10.1371/journal.pone.0027793

For peer review only - http://bmjopen.bmj.com/site/about/guidelines.xhtml

20. Berkley JA, Ross A, Mwangi I, et al. Prognostic indicators of early and late death in children admitted to district hospital in Kenya: cohort study. *BMJ (Clinical Research Ed)* 2003;326(7385):361-61.
21. George EC, Walker AS, Kiguli S, et al. Predicting mortality in sick African children: the FEAST Paediatric Emergency Triage (PET) Score. *BMC Medicine* 2015;13(1):1-12. doi: 10.1186/s12916-015-0407-3
22. Helbok R, Kendjo E, Issifou S, et al. The Lambaréné Organ Dysfunction Score (LODS) is a simple clinical predictor of fatal malaria in African children. *Journal of Infectious Diseases* 2009;200(12):1834-41. doi: 10.1086/648409
23. Erdman LK, Dhabangi A, Musoke C, et al. Combinations of host biomarkers predict mortality among Ugandan children with severe malaria: a retrospective case-control study. *Plos One* 2011;6(2):e17440-e40. doi: 10.1371/journal.pone.0017440
24. Emukule GO, McMorrow M, Ulloa C, et al. Predicting mortality among hospitalized children with respiratory illness in Western Kenya, 2009-2012. *Plos One* 2014;9(3):e92968-e68. doi: 10.1371/journal.pone.0092968
25. Hooli S, Colbourn T, Lufesi N, et al. Predicting Hospitalised Paediatric Pneumonia Mortality Risk: An External Validation of RISC and mRISC, and Local Tool Development (RISC-Malawi) from Malawi. *PLoS ONE* 2016;11(12):1-13. doi: 10.1371/journal.pone.0168126
26. Gallagher KE, Knoll MD, Prosperi C, et al. The Predictive Performance of a Pneumonia Severity Score in Human Immunodeficiency Virus–negative Children Presenting to Hospital in 7 Low-and Middle-income Countries. 2019
27. Dramaix M, Brasseur D, Donnen P, et al. Prognostic indices for mortality of hospitalized children in central Africa. *American Journal Of Epidemiology* 1996;143(12):1235-43.
28. Olson D, Davis NL, Milazi R, et al. Development of a severity of illness scoring system (inpatient triage, assessment and treatment) for resource-constrained hospitals in developing countries. *Tropical Medicine & International Health* 2013;18(7):871-78. doi: 10.1111/tmi.12137
29. Rosman SL, Karangwa V, Law M, et al. Provisional validation of a pediatric early warning score for resource-limited settings. *Pediatrics* 2019;143(5):e20183657.
30. Collins GSP, Reitsma JBMDP, Altman DGD, et al. Transparent Reporting of a multivariable prediction model for Individual Prognosis Or Diagnosis (TRIPOD): The TRIPOD Statement. *Annals of Internal Medicine* 2015;162(1):55-63.
31. Moons KGMP, Altman DGD, Reitsma JBMDP, et al. Transparent Reporting of a multivariable prediction model for Individual Prognosis Or Diagnosis (TRIPOD): Explanation and Elaboration. *Annals of Internal Medicine* 2015;162(1):W1-W73.
32. Mpimbaza A, Sears D, Sserwanga A, et al. Admission Risk Score to Predict Inpatient Pediatric Mortality at Four Public Hospitals in Uganda. *Plos One* 2015;10(7):e0133950-e50. doi: 10.1371/journal.pone.0133950
33. Concato J, Feinstein AR, Holford TR. The risk of determining risk with multivariable models. *Annals of internal medicine* 1993;118(3):201-10.
34. Leisman DE, Harhay MO, Lederer DJ, et al. Development and reporting of prediction models: guidance for authors from editors of respiratory, sleep, and critical care journals. *Critical care medicine* 2020;48(5):623.
35. Vijay Kotu BD. Feature Selection. In: Vijay Kotu BD, ed. Data Science Concepts and Practice2019:467-90.
36. Debray T, Moons KG, Ahmed I, et al. A framework for developing, implementing, and evaluating clinical prediction models in an individual participant data meta-analysis. *Statistics in medicine* 2013;32(18):3158-80.
37. Kumar N, Thomas N, Singhal D, et al. Triage score for severity of illness. *Indian pediatrics* 2003;40(3):204-10.

For peer review only - http://bmjopen.bmj.com/site/about/guidelines.xhtml

38. Lowlaavar N, Larson CP, Kumbakumba E, et al. Pediatric in-Hospital Death from Infectious Disease in Uganda: Derivation of Clinical Prediction Models. *Plos One* 2016;11(3):e0150683-e83. doi: 10.1371/journal.pone.0150683
39. Justice AC, Covinsky KE, Berlin JA. Assessing the generalizability of prognostic information. *Annals of internal medicine* 1999;130(6):515-24.
40. Kattan MW, Harrell FE. We should not be so quick to abandon the use of domain experts and full models (letter commenting: J Clin Epidemiol. 2015; 71C: 76--85.). *Journal of clinical epidemiology* 2016;75:131.
41. Concato J, Peduzzi P, Holford TR, et al. Importance of events per independent variable in proportional hazards analysis I. Background, goals, and general strategy. *Journal of clinical epidemiology* 1995;48(12):1495-501.
42. Peduzzi P, Concato J, Feinstein AR, et al. Importance of events per independent variable in proportional hazards regression analysis II. Accuracy and precision of regression estimates. *Journal of clinical epidemiology* 1995;48(12):1503-10.
43. Peduzzi P, Concato J, Kemper E, et al. A simulation study of the number of events per variable in logistic regression analysis. *Journal of clinical epidemiology* 1996;49(12):1373-79.
44. A bootstrapping approach to reduce over-fitting in genetic programming. Proceedings of the 15th annual conference companion on Genetic and evolutionary computation; 2013. ACM.
45. Harrell FE. Regression Modeling Strategies. *BIOS* 2014;330
46. White IR, Royston P, Wood AM. Multiple imputation using chained equations: Issues and guidance for practice. *Stat Med* 2011;30(4):377-99. doi: 10.1002/sim.4067
47. Moons KG, de Groot JA, Bouwmeester W, et al. Critical appraisal and data extraction for systematic reviews of prediction modelling studies: the CHARMS checklist. *PLoS medicine* 2014;11(10):e1001744.
48. Molenberghs G, Fitzmaurice G, Kenward MG, et al. Handbook of missing data methodology: CRC Press 2014.
49. Azur MJ, Stuart EA, Frangakis C, et al. Multiple imputation by chained equations: what is it and how does it work? *International journal of methods in psychiatric research* 2011;20(1):40-49.
50. Gachau S, Quartagno M, Njagi EN, et al. Handling missing data in modelling quality of clinician-prescribed routine care: Sensitivity analysis of departure from missing at random assumption. *Statistical Methods in Medical Research* 2020:0962280220918279.
51. Bitwe R, Dramaix M, Hennart P. Simplified prognostic model of overall intrahospital mortality of children in central Africa. *Tropical medicine & international health: TM & IH* 2006;11(1):73.
52. Collins GS, Ogundimu EO, Cook JA, et al. Quantifying the impact of different approaches for handling continuous predictors on the performance of a prognostic model. *Statistics in medicine* 2016;35(23):4124-35.
53. Royston P, Altman DG, Sauerbrei W. Dichotomizing continuous predictors in multiple regression: a bad idea. *Statistics in medicine* 2006;25(1):127-41.
54. Leisman DE. Rare events in the ICU: An emerging challenge in classification and prediction. *Critical Care Medicine* 2018;46(3):418-24.
55. Ensor J, Riley RD, Moore D, et al. Systematic review of prognostic models for recurrent venous thromboembolism (VTE) post-treatment of first unprovoked VTE. *BMJ open* 2016;6(5):e011190.
56. Smit HA, Pinart M, Antó JM, et al. Childhood asthma prediction models: a systematic review. *The Lancet Respiratory Medicine* 2015;3(12):973-84. doi: 10.1016/S2213-2600(15)00428-2
57. Hodgson LE, Sarnowski A, Roderick PJ, et al. Systematic review of prognostic prediction models for acute kidney injury (AKI) in general hospital populations. *BMJ open* 2017;7(9):e016591.

For peer review only - http://bmjopen.bmj.com/site/about/guidelines.xhtml

58. Collins GS, de Groot JA, Dutton S, et al. External validation of multivariable prediction models: a systematic review of methodological conduct and reporting. *BMC medical research methodology* 2014;14(1):40.
59. Collins GS, Omar O, Shanyinde M, et al. A systematic review finds prediction models for chronic kidney disease were poorly reported and often developed using inappropriate methods. *Journal of clinical epidemiology* 2013;66(3):268-77.
60. Fahey M, Crayton E, Wolfe C, et al. Clinical prediction models for mortality and functional outcome following ischemic stroke: A systematic review and meta-analysis. *PloS one* 2018;13(1):e0185402.
61. Logullo P, MacCarthy A, Kirtley S, et al. Reporting guideline checklists are not quality evaluation forms: they are guidance for writing. *Health Science Reports* 2020;3(2)
62. Glasziou P, Altman DG, Bossuyt P, et al. Reducing waste from incomplete or unusable reports of biomedical research. *The Lancet* 2014;383(9913):267-76.
63. Kwakkel G, Wagenaar RC, Kollen BJ, et al. Predicting disability in stroke—a critical review of the literature. *Age and ageing* 1996;25(6):479-89.
64. Laupacis A, Sekar N. Clinical prediction rules: a review and suggested modifications of methodological standards. *Jama* 1997;277(6):488-94.
65. Counsell C, Dennis M. Systematic review of prognostic models in patients with acute stroke. *Cerebrovascular diseases* 2001;12(3):159-70.
66. Jacob M, Lewsey JD, Sharpin C, et al. Systematic Review and Validation of Prognostic Models in Liver Transplantation. *Liver Transplantation* 2005;11(7):814-25.
67. Moons KGM, Kengne AP, Grobbee DE, et al. Risk prediction models: II. External validation, model updating, and impact assessment. *Heart* 2012;98(9):691-98.
68. Damen JA, Hooft L, Schuit E, et al. Prediction models for cardiovascular disease risk in the general population: systematic review. *Bmj* 2016;353:i2416.
69. Cruz GD, Strathe AB, Rossow HA, et al. Characterizing bovine adipocyte distribution and its relationship with carcass and meat characteristics using a finite mixture model. *J Anim Sci* 2012;90(9):2995-3002. doi: 10.2527/jas.2011-4838
70. Singendonk MMJ, Tabbers MM, Benninga MA, et al. Pediatric Gastroesophageal Reflux Disease: Systematic Review on Prognosis and Prognostic Factors. *Journal Of Pediatric Gastroenterology And Nutrition* 2017 doi: 10.1097/MPG.0000000000001697
71. Carrillo-Larco RM, Altez-Fernandez C, Pacheco-Barrios N, et al. Cardiovascular Disease Prognostic Models in Latin America and the Caribbean: A Systematic Review. *Global heart* 2019;14(1):81-93.
72. Vickers AJ, Cronin AM. Everything you always wanted to know about evaluating prediction models (but were too afraid to ask). *Urology* 2010;76(6):1298-301.
73. Irimu G, Ogero M, Mbevi G, et al. Approaching quality improvement at scale: a learning health system approach in Kenya. *Archives of disease in childhood* 2018;103(11):1013-19.
74. Ogero M, Akech S, Malla L, et al. Examining which clinicians provide admission hospital care in a high mortality setting and their adherence to guidelines: an observational study in 13 hospitals. *Archives of Disease in Childhood* 2020
75. Ogero M, Ayieko P, Boniface Makone TJ, et al. An observational study of monitoring of vital signs in children admitted to Kenyan hospitals: an insight into the quality of nursing care? *Journal of global health* 2018;8(1)
76. Collins GS, Moons KG. Comparing risk prediction models: British Medical Journal Publishing Group, 2012.
77. Sun G-W, Shook TL, Kay GL. Inappropriate use of bivariable analysis to screen risk factors for use in multivariable analysis. *Journal of clinical epidemiology* 1996;49(8):907-16.

For peer review only - http://bmjopen.bmj.com/site/about/guidelines.xhtml

78. Wasserstein RL, Lazar NA. The ASA statement on p-values: context, process, and purpose: Taylor & Francis, 2016.
79. Austin PC, Allignol A, Fine JP. The number of primary events per variable affects estimation of the subdistribution hazard competing risks model. *Journal of clinical epidemiology* 2017;83:75-84.
80. Austin PC, Steyerberg EW. Events per variable (EPV) and the relative performance of different strategies for estimating the out-of-sample validity of logistic regression models. *Statistical methods in medical research* 2017;26(2):796-808.

Captions

Figure 1: PRISMA flow diagram showing the process used to identify prognostic models predicting in-hospital paediatric mortality included in this review.

Figure 2: Prognostic models predicting in-hospital paediatric mortality identified by country. Text highlighted in red are the names of the models with their corresponding discrimination measures (area under the curve). Key: PEWS-RL score=Paediatric Early Warning Score for Resource-Limited Settings; SICK score=Signs of Inflammation in Children that Kill; PET score= Paediatric Emergency Triage; mRISC score= Modified Respiratory Index of Severity in Children score; RISC score= Respiratory Index of Severity in Children score; PERCH severity score= Pneumonia Etiology Research for Child Health severity score; LOD score= Lambarene Organ Dysfunction score; CRT= Classification and Regression Trees; ITAT Score= Inpatient Triage Assessment and Treatment score; PEDIA score= Paediatric Early Death Index for Africa score.

Figure 3: Top four categories of predictors in the models of the reviewed reports: altered consciousness (coma, prostration, not alert, unconscious); malnutrition indicators (kwashiorkor, edema, weight-for-height z-score, weight-for-age z-score, mid-upper arm circumference-MUAC, wasting); vital signs (temperature, respiratory rate, heart rate, oxygen saturation); signs of respiratory distress (indrawing, lung crepitation, difficult breathing, grunting).

For peer review only - http://bmjopen.bmj.com/site/about/guidelines.xhtml

Figure 4: Summary of the risk of bias of the included models using PROBAST (Prediction study Risk of Bias Assessment Tool).

Figure 5: Risk of bias assessment. Low means low risk of bias, High means a high risk of bias, and Unclear bias means it was not possible to assess the risk of bias. Key: PEWS-RL score=Paediatric Early Warning Score for Resource-Limited Settings; SICK score=Signs of Inflammation in Children that Kill; PET score= Paediatric Emergency Triage; mRISC score= Modified Respiratory Index of Severity in Children score; RISC score= Respiratory Index of Severity in Children score; PERCH severity score= Pneumonia Etiology Research for Child Health severity score; LOD score= Lambarene Organ Dysfunction score; CRT= Classification and Regression Trees; ITAT Score= Inpatient Triage Assessment and Treatment score; PEDIA score= Paediatric Early Death Index for Africa score.

For peer review only

For peer review only - http://bmjopen.bmj.com/site/about/guidelines.xhtml

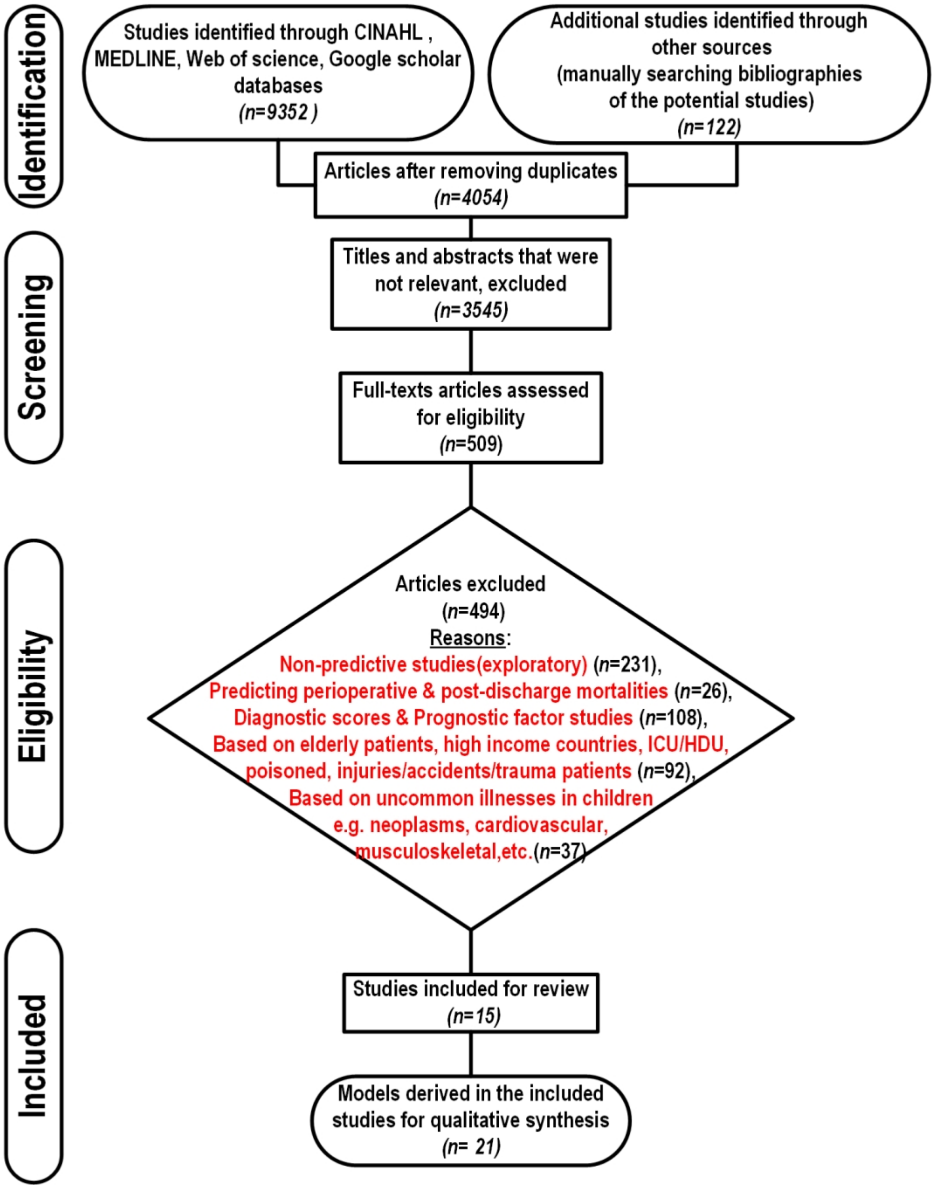

Figure 1: PRISMA flow diagram showing the process used to identify prognostic models predicting in-hospital paediatric mortality included in this review

127x164mm (300 x 300 DPI)

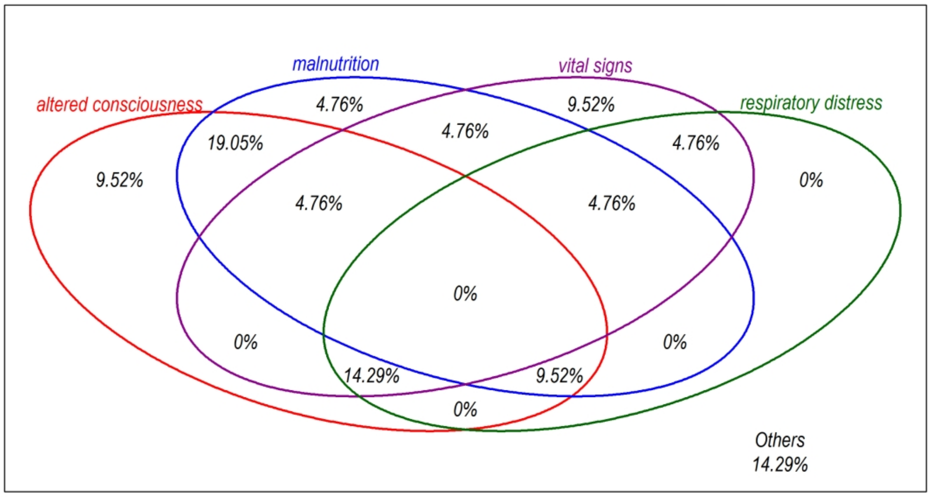

Figure 3: Top four categories of predictors in the models of the reviewed reports: altered consciousness (coma, prostration, not alert, unconscious); malnutrition indicators (kwashiorkor, edema, weight-for-height z-score, weight-for-age z-score, mid-upper arm circumference-MUAC, wasting); vital signs (temperature, respiratory rate, heart rate, oxygen saturation); signs of respiratory distress (indrawing, lung crepitation, difficult breathing, grunting).

228x127mm (300 x 300 DPI)

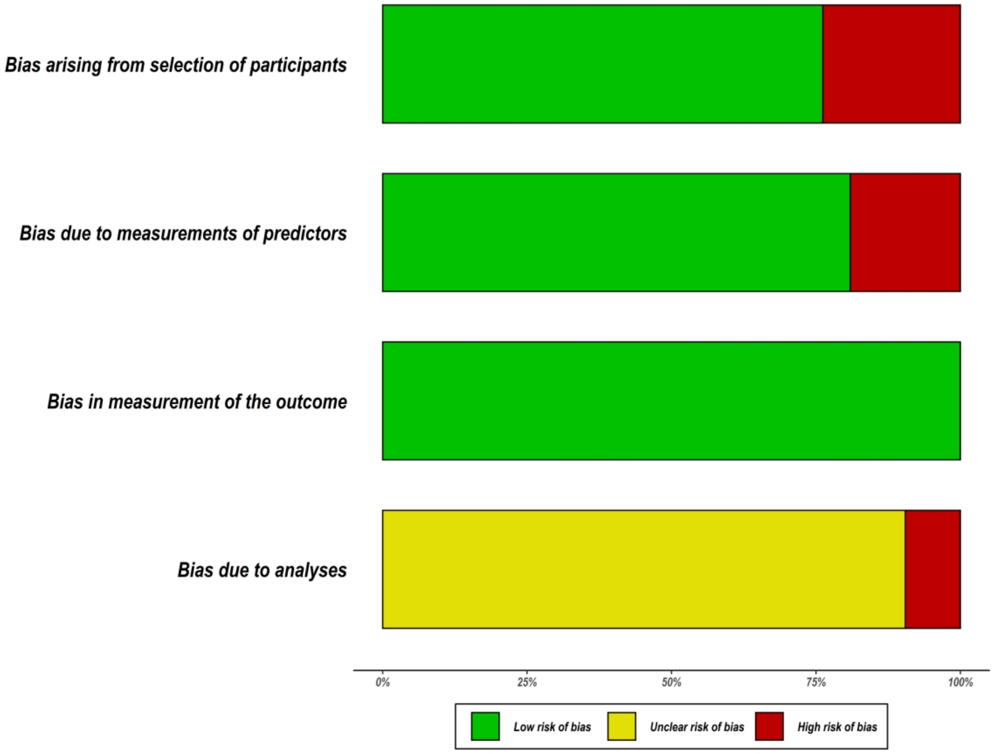

Figure 4: Summary of the risk of bias of the included models using PROBAST (Prediction study Risk of Bias Assessment Tool).

254x203mm (300 x 300 DPI)

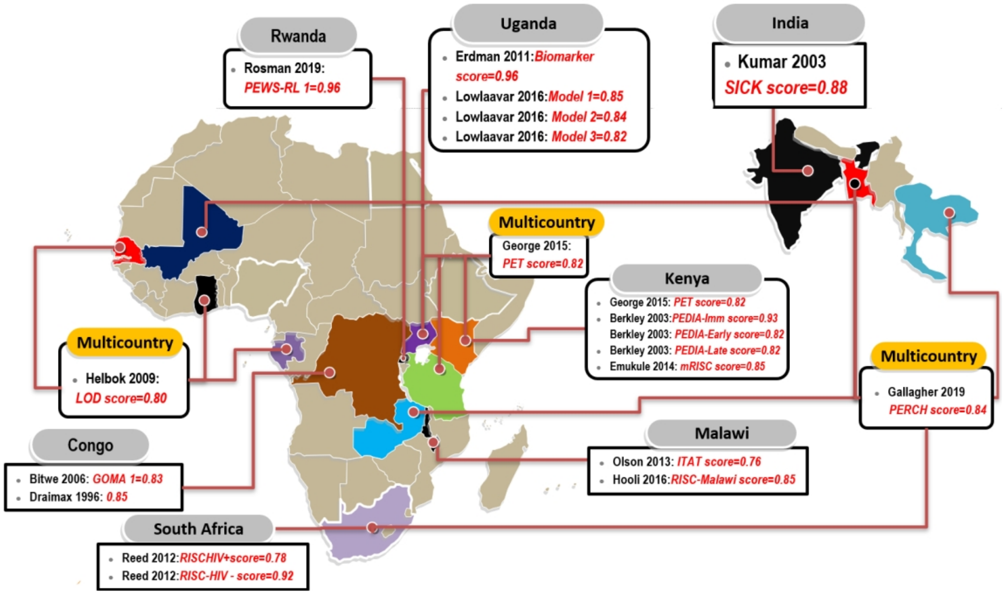

Figure 2: Prognostic models predicting in-hospital paediatric mortality identified by country. Text highlighted in red are the names of the models with their corresponding discrimination measures (area under the curve). Key: PEWS-RL score=Paediatric Early Warning Score for Resource-Limited Settings; SICK score=Signs of Inflammation in Children that Kill; PET score= Paediatric Emergency Triage; mRISC score= Modified Respiratory Index of Severity in Children score; RISC score= Respiratory Index of Severity in Children score; PERCH severity score= Pneumonia Etiology Research for Child Health severity score; LOD score= Lambarene Organ Dysfunction score; CRT= Classification and Regression Trees; ITAT Score= Inpatient Triage Assessment and Treatment score; PEDIA score= Paediatric Early Death Index for Africa score.

137x81mm (300 x 300 DPI)

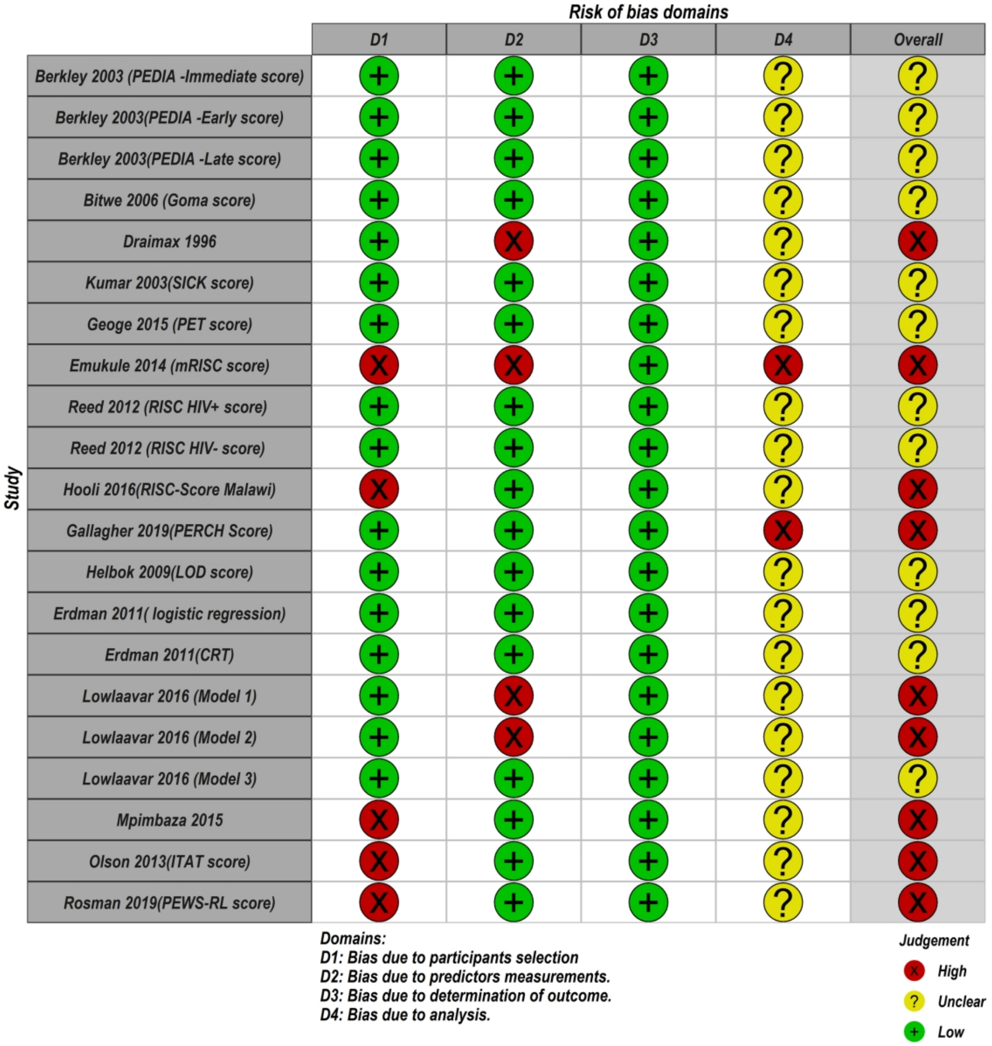

Figure 5: Risk of bias assessment. Low means low risk of bias, High means a high risk of bias, and Unclear bias means it was not possible to assess the risk of bias. Key: PEWS-RL score=Paediatric Early Warning Score for Resource-Limited Settings; SICK score=Signs of Inflammation in Children that Kill; PET score= Paediatric Emergency Triage; mRISC score= Modified Respiratory Index of Severity in Children score; RISC score= Respiratory Index of Severity in Children score; PERCH severity score= Pneumonia Etiology Research for Child Health severity score; LOD score= Lambarene Organ Dysfunction score; CRT= Classification and Regression Trees; ITAT Score= Inpatient Triage Assessment and Treatment score; PEDIA score= Paediatric Early Death Index for Africa score.

199x219mm (300 x 300 DPI)

Table 1: Systematic review framework as recommended by CHARMS checklist

Item	Criteria
Prognostic or diagnostic model	Prognostic model predicting in-hospital mortality.
Scope	Prognostic models to inform clinicians about the risk of deterioration or death.
Type of prediction models	Prognostic models with and/or without external validation.
Prediction target population	Children aged > 1 month to 15 years admitted in pediatric wards in developing countries
Outcome of interest	All-cause in-hospital mortality.
Prediction period	Any
Intended moment to apply the prediction tool	Prognostic model to be used in primary prevention to assess risk of deterioration and thus guide prevention/treatment.

KEY:

CHARMS= Checklist for critical Appraisal and data extraction for systematic Reviews of prediction Modelling Studies

For peer review only

Table 2: Search terms for prognostic models

Search ID	Sub-heading	Search Terms
S4	Children	paediatric* OR pediatric* OR (MH "Pediatrics+") OR child*
S3	Hospital based	(MH "Hospitals+") OR hospital*
S2	Low-income countries	(MH "Developing Countries+") OR (MH "Africa+") OR TI ("low income" OR "low and middle income"OR "LMIC" OR "LIC" OR "limited resource*" OR "poor resource*" OR "resource* poor" OR ("developing countries") OR ("developing nations") OR ("third world") OR "resource-constrained" OR ("global south")
S1	Predictive models	prognos* OR (MH "prognosis") OR (Predict* AND (Outcome* OR Risk* OR Model* OR Mortality OR Index OR Rule* OR decision* OR scor*)) OR "risk score" OR "scor* system" OR "logistic model*" "risk prediction" OR "risk calculation" OR "risk assessment" OR "c statistic" OR discrimination OR calibration OR AUC OR "area under the curve" OR "area under the receiver operator characteristic curve"

For peer review only

Table 3: List of domains and signaling questions used for assessment of risk of bias according to the PROBAST tool.

Domain	Signalling question
**Participants selection**	Were appropriate data sources used, e.g., cohort, RCT, or nested case–control study data?
	Were all inclusions and exclusions of participants appropriate?
**Predictors**	Were predictors defined and assessed in a similar way for all participants?
	Were predictor assessments made without knowledge of outcome data?
	Are all predictors available at the time the model is intended to be used?
**Outcome**	Was the outcome determined appropriately?
	Was a prespecified or standard outcome definition used?
	Were predictors excluded from the outcome definition?
	Was the outcome defined and determined in a similar way for all participants?
	Was the outcome determined without knowledge of predictor information?
	Was the time interval between predictor assessment and outcome determination appropriate?
**Analysis**	Were there a reasonable number of participants with the outcome?
	Were continuous and categorical predictors handled appropriately?
	Were all enrolled participants included in the analysis?
	Were participants with missing data handled appropriately?
	Was selection of predictors based on univariable analysis avoided?
	Were complexities in the data (e.g., censoring, competing risks, sampling of control participants) accounted for appropriately?
	Were relevant model performance measures evaluated appropriately?
	Were model overfitting, underfitting, and optimism in model performance accounted for?
	Do predictors and their assigned weights in the final model correspond to the results from the reported multivariable analysis?

KEY:

PROBAST= Prediction study Risk of Bias Assessment Tool

For peer review only

Study	***Model name***	***Country***	***Source of data***	***Study year***	***Inclusion criteria***	***Age***	***Ouctome***
Berkley 2003	PEDIA Immediate death	Kenya	Prospective cohort	1998-2000	Aged over 90 days	3 months-13 years	Mortality
Berkley 2003	PEDIA Early death	Kenya	Prospective cohort	1998-2000	Aged over 90 days	3 months-13 years	Mortality
Berkley 2003	PEDIA Late death	Kenya	Prospective cohort	1998-2000	Aged over 90 days	3 months-13 years	Mortality
Bitwe 2006	Goma 1 Model	Democratic Republic of Congo	Prospective cohort	2003-2004	<12 months	Median: 12.8 months	Mortality

For peer review only

Draimax 1996		Congo	Prospective cohort	1986-1988	Malnutrition	Median: 27 months	Mortality
Kumar 2003	SICK score	India	Prospective cohort	1998-1999	Paediatric patients	No Information	Mortality
Geoge 2015	PET Score	Kenya, Uganda, Tanzania	RCT	2009-2011	Malaria	Median: 24 (IQR=13-38)	Mortality
Emukule 2014	mRISC	Kenya	Surveillance	2009-2012	Under 5 years hospitalized with severe acute respiratory illness	<59 months	Mortality
Reed 2012	RISC-HIV positive	South Africa	RCT	1998-2001	LRTI hospitalizations under 24 months with HIV infection	<24 months	Mortality

For peer review only

Reed 2012	RISC-HIV Negative	South Africa	RCT	1998-2001	LRTI hospitalizations under 24 months without HIV infection	<24 months	Mortality
Hooli 2016	RISC-Malawi	Malawi	Retrospective observational study	2011-2014	0-59 months hospitalized with pneumonia	<59 months	Mortality
Gallagher 2019	PERCH Score	Kenya, Zambia, South Africa, Mali, Gambia, Bangladesh, Thailand	Case-control study	2011–2014	1–59 months HIV negative hospitalized with severe or very severe pneumonia	Median: 9(4-19) months	In-hospita
Helbok 2009	LOD score	Gambia,Malawi,Kenya, Ghana,Gabon	Prospective cohort	2000-2005	Hospitalized children with severe malaria	28(0-180)	Mortality
Erdman 2011 (Logistic regression)	Biomarker score	Uganda	Retrospective neste	2007-2009	6 months - 12 years	6 months - 12 years	Mortality
Erdman 2011 (Classification tree)		Uganda	Retrospective neste	2007-2009	6 months - 12 years	6 months - 12 years	Mortality

For peer review only

 BMJ Open

Lowlaavar 2016	Model 1	Uganda	Prospective observa	2012-2013	6–60 months admitted with infectious illness	Median 18.2 (IQR 11.9–33.1) months	Mortality
Lowlaavar 2016	Model 2	Uganda	Prospective observa	2012-2013	6–60 months admitted with infectious illness	Median 18.2 (IQR 11.9–33.1) months	Mortality
Lowlaavar 2016	Model 3	Uganda	Prospective observa	2012-2013	6–60 months admitted with infectious illness	Median 18.2 (IQR 11.9–33.1) months	Mortality
Mpimbaza 2015		Uganda	Surveillance	2010-2013	General paediatrics	18 months (IQR 9–36)	Mortality
Olson 2013	ITAT score	Malawi	Nested case–control	2010-2011	age <15 years on the acute care and malnutrition wards	≤15 years	Mortality
Rosman 2019	PEWS-RL	Rwanda	Case-control study	2016-2017	0-18 years patients admitted to pediatric department	0-18 years	Mortality

For peer review only

Sample size	*Number of outcome events*	*Missing data handling*	*Number of participant with missing data reported?*	*Regression method*	*Were model assumptions verified*	*Predictor selection*	*Was a shrinkage method used*
429	60	No Information	No Information	Spiegelhalter/Knill-Jones method	Yes	Univariate	No Information
439	193	No Information	No Information	Spiegelhalter/Knill-Jones method	Yes	Univariate	No Information
436	183	No Information	No Information	Spiegelhalter/Knill-Jones method	Yes	Univariate	No Information
414	66	No Information	No Information	Logistic regression	Yes	Univariate & Stepwise	No Information

For peer review only

1129	196	No Information	No Information	Logistic regression	No Information	A priori	No Information
1099	44	No Information	No Information	Logistic regression	No Information	Univariate(but included all variables in final model)	No Information
3170	315	Complete case analyses	Yes	Cox proportional hazards regression	No Information	A priori	No Information
3581	218	Complete case analyses	No Information	Logistic regression	Yes	Univariate	Yes
1502	265	Complete case analyses	No Information	Logistic regression	No Information	Univariate	No Information

For peer review only

2646	33	Complete case analyses	No Information	Logistic regression	No Information	Univariate	No Information
14665	464	Multiple imputation	Yes	Logistic regression	Yes	A priori	No Information
1802	120	Complete case analyses	No Information	Logistic regression	No Information	Univariate	No Information
23980	1004	Complete case analyses	Yes	Logistic regression	No Information	Forward & backward Stepwise	No Information
103	23	No missing values	Yes	Logistic regression	Yes	Univariate	No Information
103	23	No missing values	Yes	Classification tree	No Information	No Information	No Information

For peer review only

1307	65	Multiple imputation	No Information	Logistic regression	No Information	Univariate & Stepwise	No Information
1307	65	Multiple imputation	No Information	Logistic regression	No Information	Univariate & Stepwise	No Information
1307	65	Multiple imputation	No Information	Logistic regression	No Information	Univariate & Stepwise	No Information
50249	1742	Complete case analyses	Yes	Logistic regression	No Information	Backward	No Information
1606	54	Single imputation	Yes	Logistic regression	Yes	Univariate	No Information
168	57	Complete case analyses	No Information	Logistic regression	No Information	Univariate	No Information

For peer review only

Calibration method	***Discrimination***	***Classification measures reported***	***Method used for internal validation***	***External validation***	***Was a simplified model presented***	***Were coefficients(including intercept) of the regression model presented***	***Number of predictors in final model***
No Information	0.93(0.92-0.94)	No Information	Separate dataset	Yes	Yes	NA	10
No Information	0.82(0.80-0.83)	No Information	Separate dataset	Yes	Yes	NA	8
No Information	0.82(0.81-0.84)	No Information	Separate dataset	Yes	Yes	NA	9
Yes	0.83 (0.78-0.88)	No Information	No Information	No	Yes	No	8

For peer review only

No Information	0.85(No information)	Positive predictive values 40% and negative predictive value of 97.9%	Separate dataset	No	Yes	No	4
No Information	0.89	Maximum discrimination was observed at a score of 2.5 with a sensitivity of 84.1% and of specificity 82.2%	No Information	Yes	Yes	No	9
Hosmer-Lemeshow test, P=0.30	0.82(0.77–0.87)	No Information	Separate dataset	No	Yes	No	11
Calibration plot	0.85	A score of >6 has a sensitivity of 1.8% and specificity 99.9%	Bootstrapping	Yes	Yes	No	9
Hosmer-Lemeshow test, P=0.95	0.78	Score of 7 has a sensitivity of 4% and specificity of 99%	Bootstrapping	No	Yes	No	7

For peer review only

Hosmer-Lemeshow test, P=0.87	0.92	Score of 6 has a sensitivity: 16% Specificity: 99%	Bootstrapping	Yes	Yes	No	5
Risk predictiveness curve	0.79 (95% CI: 0.76±0.82)	a score of 8 has sensitivity of 57% and specificity of 88%	No Information	No	Yes	Yes	5
Calibration plot	0.84(No Information)	positive predictive value 23.6%, positive predictive value 95.8%	Bootstrapping & s	No	Yes	No	12
No Information	80 (79–82)	LODS ┐>=1, sensitivi ty was 85% and specif icity was 63%	No Information	Yes	Yes	No	8
Hosmer-Lemeshow test and calibration slope analysis	0.96(0.90–0. 99)	sensitivity of 95.7% (95% CI: 78.1–99.9) and specificity of 88.8% (79.7–94.7) predicting death	Boostrappling	No	Yes	No	8
No Information	No Information	100% sensitivity and 92.5% specificity for predicting outcome	10-fold cross vali	No	No	No	3

For peer review only

No Information	0.85 (0.80–0.89)	Sensitive: 0.83 (0.74–0.92), Specificity: 0.76 (0.73–0.78)	No Information	No	No	Yes	3
No Information	0.84 (0.79–0.89)	Sensitive: 0.80 (0.70–0.90), Specificity: 0.76 (0.74–0.79)	No Information	No	No	Yes	3
No Information	0.82 (0.72–0.91)	Sensitive: 0.82 (0.72–0.91), Specificity: 0.71 (0.68–0.73)	No Information	No	No	Yes	2
General paediatrics	0.76(No information)	No Information	Separate dataset	No	Yes	No	13
No Information	0.76(No information)	sensitivity: 0.44, specificity: 0.86, PPV: 0.18, NPV: 0.96 for a cut-off of 4	No Information	No	Yes	No	4
No Information	0.96 (95% CI 0.93–0.99).	PEWS-RL of >=3, sensitivity was 96.2%, and specificity was 87.3%	No Information	No	Yes	No	6

For peer review only

Predictors in the final model	***Are there laboratory based predictors***	***Handling of continous predictors***	***Events per variable***
Severe anaemia, Jaundice, Subcostal indrawing, Deep breathing, Prostrated with seizures, Prostrated without seizures, Impaired consciousness with seizures, Impaired consciousness without seizures, Axillary temperature <36 °C, Axillary temperature >39 °C	No	NA	6
Jaundice, Subcostal indrawing, Prostrated with seizures, Prostrated without seizures, Impaired consciousness with seizures, Impaired consciousness without seizures,Wasting, Kwashiorkor	No	NA	24.125
History >7 days, Prostrated with seizures, Prostrated without seizures, Impaired consciousness with seizures, Impaired consciousness without seizures, Axillary temperature <36 °C, Axillary temperature >39 °C, Wasting, Kwashiorkor	No	Dichotomized History	20.33333
Age(<12, >=12months), Brachial Perimeter(<=115mm, >115mm), State of consciousness(Unconscious, Aware), Infectious diagnosis(Acute respiratory infection, Malaria, Gastroenteritis, Septicemia / bacteremia, Other infections)	No	Dichotomized Brachial perimeter & Age	8.25

For peer review only

MUAC, edema, Serum albumin, Transthyretin	Yes	MUAC	
Temperature(Normal, Abnormal), Heart rate(Normal, Abnormal), Respiratory rate(Normal, Abnormal), Systolic blood pressure(Normal, Abnormal), Capillary refill time(Normal, Abnormal), Consciousness(Normal, Abnormal), Age(≥60, ≥12 to <60, ≥1to<12, <1)	No	Dichotomized most variables	4.888889
Temperature(≤37, >37), Heart rate(<80 bpm, ≥80 to <105 bpm, ≥220 bpm), Capillary refill time(≥2sec, <2sec), Conscious level(prostrate, coma), Respiratory distress, Lung crepitations, Severe pallor, Weak pulse, Weight(<6 kg, 6–8 kg), Deep breathing	No	multivariable fractional polynomials	28.63636
Lab confirmed malaria, Weight for age(Low, Very Low), Dehydration, Unconscious, Unable to drink/breastfeed, Night sweats, Chest wall in-drawing, Interaction between malaria and chest wall in-drawing, A.V.P.U scale - Not alert	Yes	Categorized weight for age	24.22222
Oxygen saturation <90%, Chest indrawing, Wheezing, Refusing feeds, HIV classification(Severe, Mild or moderate), IMCI age group(<2 months, 3–12 months)	No		37.85714

For peer review only

Oxygen saturation <90%, Chest indrawing, Wheezing, Refusing feeds, Weight for age(Low (<= -2 z-score), Very Low (<= -3 z-score))	No	Categorized weight for age	6.6
Oxygen saturation(moderate, severe), MUAC(moderate, severe), Gender, Wheeze, Consciousness	No	Categorized MUAC and Oxygen saturation	92.8
Age(1-11, 12-59), sex, Unresponsiveness and/or deep breathing(Deep breathing, but alert, Unresponsive but no deep breathing, Unresponsive and deep breathing), cough, grunting, hypoxemia, length of illness(0–2, 3–5, >5), Weight-for-height z-score(Very low (< –3), Low (≥ –3 to < –2), Normal-high (≥ –2))	No	Categorized most variables	10
Convulsion, vomiting, deep breathing, intercostal recession, Coma, Prostration,hyperparastemia, severe anemia	Yes	NA	125.5
angiopoietin-2, soluble ICAM-1, soluble Flt-1, procalcitonin, IP-10, soluble TREM-1, age, parasitemia	Yes	NA	2.875
IP-10, Ang-2, sICAM-1	Yes	NA	7.666667

For peer review only

Abnormal BCS, Positive HIV diagnosis, Weight-age z-score	Yes	Treated as continuous	21.66667
Abnormal BCS, HIV diagnosis, MUAC	Yes	Treated as continuous	21.66667
Abnormal BCS, MUAC	No	Treated as continuous	32.5
Age, fever, difficulty breathing, altered consciousness, unable to drink or breastfeed, convulsions, temperature, unconsciousness, pallor, jaundice, deep breathing, meningeal signs, unable to sit up	No	NA	134
Oxygen saturation, Temperature, Heart rate, Respiratory rate	No	Used splines	13.5
PEWS-RL score(0 to 6)	No	NA	9.5

For peer review only

**Study**	**Participants**	
	Were appropriate data sources used, e.g., cohort, RCT, or nested case–control study data?	*Were all inclusions and exclusions of participants appropriate?*
Berkley 2003 (PEDIA -Immediate)	Yes	Yes
Berkley 2003(PEDIA -Early)	Yes	Yes
Berkley 2003(PEDIA -Late)	Yes	Yes
Bitwe 2006 (Goma score)	Yes	Yes
Draimax 1996	Yes	Yes
Kumar 2003(SICK score)	Yes	Yes
Geoge 2015 (PET score)	Yes	Yes
Emukule 2014 (mRISC score)	No	Yes
Reed 2012 (RISC HIV+)	Yes	Yes
Reed 2012 (RISC HIV-)	Yes	Yes
Hooli 2016(RISC-Malawi)	No	Yes
Gallagher 2019(PERCH Score)	Yes	Yes
Helbok 2009(LOD score)	Yes	Yes
Erdman 2011(logistic regression)	Yes	Yes
Erdman 2011(CRT)	Yes	Yes
Lowlaavar 2016 (Model 1)	Yes	Yes
Lowlaavar 2016 (Model 2)	Yes	Yes
Lowlaavar 2016 (Model 3)	Yes	Yes
Mpimbaza 2015	No	Yes
Olson 2013(ITAT score)	Yes	Yes
Rosman 2019(PEWS-RL score)	No	Yes

For peer review only

Risk of Bias in participants	Predictors			Risk of Bias in predictors
	Were predictors defined and assessed in a similar way for all participants	*Were predictor assessments made without knowledge of outcome data?*	*Are all predictors available at the time the model is intended to be used?*	
Low	Yes	Yes	Yes	Low
Low	Yes	Yes	Yes	Low
Low	Yes	Yes	Yes	Low
Low	Yes	Yes	Yes	Low
Low	Yes	Yes	No	High
Low	Yes	Yes	Yes	Low
Low	Yes	Yes	Yes	Low
High	Yes	Yes	No	High
Low	Yes	Yes	Yes	Low
Low	Yes	Yes	Yes	Low
High	Yes	Yes	Yes	Low
Low	Yes	Yes	Yes	Low
Low	Yes	Yes	Yes	Low
Low	Yes	Yes	Yes	Low
Low	Yes	Yes	Yes	Low
Low	Yes	Yes	No	High
Low	Yes	Yes	No	High
Low	Yes	Yes	Yes	Low
High	Yes	Yes	Yes	Low
Low	Yes	Yes	Yes	Low
High	Yes	Yes	Yes	Low

For peer review only

Outcome				
Was the outcome determined appropriately?	*Was a prespecified or standard outcome definition used?*	*Were predictors excluded from the outcome definition?*	*Was the outcome defined and determined in a similar way for all participants?*	*Was the outcome determined without knowledge of predictor information?*
Yes	Yes	Yes	Yes	Yes
Yes	Yes	Yes	Yes	Yes
Yes	Yes	Yes	Yes	Yes
Yes	Yes	Yes	Yes	Yes
Yes	Yes	Yes	Yes	Yes
Yes	Yes	Yes	Yes	Yes
Yes	Yes	Yes	Yes	Yes
Yes	Yes	Yes	Yes	Yes
Yes	Yes	Yes	Yes	Yes
Yes	Yes	Yes	Yes	Yes
Yes	Yes	Yes	Yes	Yes
Yes	Yes	Yes	Yes	Yes
Yes	Yes	Yes	Yes	Yes
Yes	Yes	Yes	Yes	Yes
Yes	Yes	Yes	Yes	Yes
Yes	Yes	Yes	Yes	Yes
Yes	Yes	Yes	Yes	Yes
Yes	Yes	Yes	Yes	Yes
Yes	Yes	Yes	Yes	Yes
Yes	Yes	Yes	Yes	Yes
Yes	Yes	Yes	Yes	Yes

For peer review only

Was the time interval between predictor assessment and outcome determination appropriate?	*Risk of Bias in outcome*	*Were there a reasonable number of participants with the outcome?*	*Were continuous and categorical predictors handled appropriately?*
Yes	Low	No	Yes
Yes	Low	Yes	Yes
Yes	Low	Yes	No
Yes	Low	No	No
Yes	Low	Yes	No
Yes	Low	No	No
Yes	Low	Yes	Yes
Yes	Low	Yes	No
Yes	Low	Yes	Yes
Yes	Low	No	No
Yes	Low	Yes	No
Yes	Low	Yes	No
Yes	Low	Yes	Yes
Yes	Low	No	Yes
Yes	Low	No	Yes
Yes	Low	Yes	Yes
Yes	Low	Yes	Yes
Yes	Low	Yes	Yes
Yes	Low	Yes	Yes
Yes	Low	Yes	Yes
Yes	Low	No	Yes

For peer review only

			Analysis
Were all enrolled participants included in the analysis?	*Were participants with missing data handled appropriately?*	*Was selection of predictors based on univariable analysis avoided*	*Were complexities in the data (e.g., censoring, competing risks, sampling of control participants) accounted for appropriately?*
Yes	Probably No	No	NA
Yes	Probably No	No	NA
Yes	Probably No	No	NA
Yes	No Information	No	NA
Yes	No Information	Yes	NA
Yes	No Information	No	NA
Yes	No	Yes	No
Yes	No	No	NA
Yes	No Information	No	NA
Yes	No Information	No	NA
Yes	Yes	Yes	NA
Yes	No	No	NA
Yes	No	No	NA
Yes	Yes	No	NA
Yes	Yes	No Information	NA
Yes	Yes	No	NA
Yes	Yes	No	NA
Yes	Yes	No	NA
Yes	No	No	NA
Yes	Probably No	No	NA
Yes	No	No	NA

For peer review only

Were relevant model performance measures evaluated appropriately?	*Were model overfitting, underfitting, and optimism in model performance accounted for?*	*Do predictors and their assigned weights in the final model correspond to the results from the reported multivariable analysis?*	*Risk of Bias in analysis*
Probably No	No Information	Probably Yes	Unclear
Probably No	No Information	Probably Yes	Unclear
Probably No	No Information	Probably Yes	Unclear
Yes	No Information	Yes	Unclear
No	No Information	Probably No	Unclear
No	No Information	Yes	Unclear
No	No Information	Yes	Unclear
Yes	Yes	Yes	High
Yes	Yes	Yes	Unclear
Yes	Yes	Yes	Unclear
Yes	No Information	Yes	Unclear
Yes	Yes	Yes	High
Probably No	No Information	Probably No	Unclear
No	Yes	No Information	Unclear
No Information	Yes	No Information	Unclear
No	No Information	Yes	Unclear
No	No Information	Yes	Unclear
No	No Information	Yes	Unclear
No Information	No Information	Yes	Unclear
No	No Information	No	Unclear
No	No Information	No	Unclear

For peer review only

Reporting checklist for systematic review and meta-analysis.

Based on the PRISMA guidelines.

Instructions to authors

Complete this checklist by entering the page numbers from your manuscript where readers will find each of the items listed below.

Your article may not currently address all the items on the checklist. Please modify your text to include the missing information. If you are certain that an item does not apply, please write "n/a" and provide a short explanation.

Upload your completed checklist as an extra file when you submit to a journal.

In your methods section, say that you used the PRISMAreporting guidelines, and cite them as:

Moher D, Liberati A, Tetzlaff J, Altman DG, The PRISMA Group. Preferred Reporting Items for Systematic Reviews and Meta-Analyses: The PRISMA Statement

		Reporting Item	Page Number
**Title**			
	#1	Identify the report as a systematic review, meta-analysis, or both.	1
**Abstract**			

For peer review only

Structured summary	#2	Provide a structured summary including, as applicable: background; objectives; data sources; study eligibility criteria, participants, and interventions; study appraisal and synthesis methods; results; limitations; conclusions and implications of key findings; systematic review registration number	2
**Introduction**			
Rationale	#3	Describe the rationale for the review in the context of what is already known.	3
Objectives	#4	Provide an explicit statement of questions being addressed with reference to participants, interventions, comparisons, outcomes, and study design (PICOS).	3
**Methods**			
Protocol and registration	#5	Indicate if a review protocol exists, if and where it can be accessed (e.g., Web address) and, if available, provide registration information including the registration number.	4
Eligibility criteria	#6	Specify study characteristics (e.g., PICOS, length of follow-up) and report characteristics (e.g., years considered, language, publication status) used as criteria for eligibility, giving rational	4
Information sources	#7	Describe all information sources in the search (e.g., databases with dates of coverage, contact with study authors to identify additional studies) and date last searched.	N/A
Search	#8	Present full electronic search strategy for at least one database, including any limits used, such that it could be repeated.	5

For peer review only

Study selection	#9	State the process for selecting studies (i.e., for screening, for determining eligibility, for inclusion in the systematic review, and, if applicable, for inclusion in the meta-analysis).	6
Data collection process	#10	Describe the method of data extraction from reports (e.g., piloted forms, independently by two reviewers) and any processes for obtaining and confirming data from investigators.	6
Data items	#11	List and define all variables for which data were sought (e.g., PICOS, funding sources), and any assumptions and simplifications made.	6
Risk of bias in individual studies	#12	Describe methods used for assessing risk of bias in individual studies (including specification of whether this was done at the study or outcome level, or both), and how this information is to be used in any data synthesis.	7
Summary measures	#13	State the principal summary measures (e.g., risk ratio, difference in means).	N/A
Planned methods of analyis	#14	Describe the methods of handling data and combining results of studies, if done, including measures of consistency (e.g., I2) for each meta-analysis.	N/A
Risk of bias across studies	#15	Specify any assessment of risk of bias that may affect the cumulative evidence (e.g., publication bias, selective reporting within studies).	7
Additional analyses	#16	Describe methods of additional analyses (e.g., sensitivity or subgroup analyses, meta-regression), if done, indicating which	N/A

For peer review only

		were pre-specified.	
**Results**			
Study selection	#17	Give numbers of studies screened, assessed for eligibility, and included in the review, with reasons for exclusions at each stage, ideally with a flow diagram.	8
Study characteristics	#18	For each study, present characteristics for which data were extracted (e.g., study size, PICOS, follow-up period) and provide the citation.	8
Risk of bias within studies	#19	Present data on risk of bias of each study and, if available, any outcome-level assessment (see Item 12).	11
Results of individual studies	#20	For all outcomes considered (benefits and harms), present, for each study: (a) simple summary data for each intervention group and (b) effect estimates and confidence intervals, ideally with a forest plot.	N/A
Synthesis of results	#21	Present the main results of the review. If meta-analyses are done, include for each, confidence intervals and measures of consistency.	N/A
Risk of bias across studies	#22	Present results of any assessment of risk of bias across studies (see Item 15).	11
Additional analysis	#23	Give results of additional analyses, if done (e.g., sensitivity or subgroup analyses, meta-regression [see Item 16]).	N/A
**Discussion**			

For peer review only

Summary of Evidence	#24	Summarize the main findings, including the strength of evidence for each main outcome; consider their relevance to key groups (e.g., health care providers, users, and policy makers	12
Limitations	#25	Discuss limitations at study and outcome level (e.g., risk of bias), and at review level (e.g., incomplete retrieval of identified research, reporting bias).	15
Conclusions	#26	Provide a general interpretation of the results in the context of other evidence, and implications for future research.	16

Funding

Funding	#27	Describe sources of funding or other support (e.g., supply of data) for the systematic review; role of funders for the systematic review.	17

The PRISMA checklist is distributed under the terms of the Creative Commons Attribution License CC-BY. This checklist was completed on 15. October 2019 using https://www.goodreports.org/, a tool made by the EQUATOR Network in collaboration with Penelope.ai

For peer review only

BMJ Open

Prognostic models for predicting in-hospital paediatric mortality in resource-limited countries: a systematic review

Journal:	*BMJ Open*
Manuscript ID	bmjopen-2019-035045.R2
Article Type:	Original research
Date Submitted by the Author:	03-Sep-2020

Complete List of Authors:	ogero, morris; KEMRI-Wellcome Trust Research Programme; University of Nairobi College of Biological and Physical Sciences, School of Mathematics<br>Sarguta, Rachel ; University of Nairobi College of Biological and Physical Sciences, School of Mathematics<br>Malla, Lucas; KEMRI-Wellcome Trust Research Programme Nairobi<br>Aluvaala, Jalemba ; KEMRI-Wellcome Trust Research Programme Nairobi, Health Services Unit<br>Agweyu, Ambrose; KEMRI-Wellcome Trust Research Programme Nairobi<br>English, Mike; Oxford University, Nuffield Department of Medicine and Department of Paediatrics; KEMRI-Wellcome Trust Research Programme Nairobi<br>Onyango, Nelson ; University of Nairobi College of Biological and Physical Sciences, School of Mathematics<br>Akech, Samuel; KEMRI-Wellcome Trust Research Programme Nairobi

**Primary Subject Heading**:	Paediatrics
Secondary Subject Heading:	Research methods, Paediatrics, Health services research
Keywords:	STATISTICS & RESEARCH METHODS, PAEDIATRICS, Paediatric intensive & critical care < PAEDIATRICS

SCHOLARONE™
Manuscripts

I, the Submitting Author has the right to grant and does grant on behalf of all authors of the Work (as defined in the below author licence), an exclusive licence and/or a non-exclusive licence for contributions from authors who are: i) UK Crown employees; ii) where BMJ has agreed a CC-BY licence shall apply, and/or iii) in accordance with the terms applicable for US Federal Government officers or employees acting as part of their official duties; on a worldwide, perpetual, irrevocable, royalty-free basis to BMJ Publishing Group Ltd ("BMJ") its licensees and where the relevant Journal is co-owned by BMJ to the co-owners of the Journal, to publish the Work in this journal and any other BMJ products and to exploit all rights, as set out in our licence.

The Submitting Author accepts and understands that any supply made under these terms is made by BMJ to the Submitting Author unless you are acting as an employee on behalf of your employer or a postgraduate student of an affiliated institution which is paying any applicable article publishing charge ("APC") for Open Access articles. Where the Submitting Author wishes to make the Work available on an Open Access basis (and intends to pay the relevant APC), the terms of reuse of such Open Access shall be governed by a Creative Commons licence – details of these licences and which Creative Commons licence will apply to this Work are set out in our licence referred to above.

Other than as permitted in any relevant BMJ Author's Self Archiving Policies, I confirm this Work has not been accepted for publication elsewhere, is not being considered for publication elsewhere and does not duplicate material already published. I confirm all authors consent to publication of this Work and authorise the granting of this licence.

For peer review only

Prognostic models for predicting in-hospital paediatric mortality in resource-limited countries: a systematic review

Morris Ogero[1, 2], Rachel Sarguta[2], Lucas Malla[1], Jalemba Aluvaala[1], Ambrose Agweyu[1], Mike English[1, 3], Nelson Onyango[2], Samuel Akech[1]

[1]Kenya Medical Research Institute (KEMRI)-Wellcome Trust Research Programme, Nairobi, Kenya; [2]School of Mathematics, University of Nairobi, Kenya; and [3]Nuffield Department of Medicine, University of Oxford, UK.

Correspondence: Morris Ogero,

KEMRI / Wellcome Trust Research Programme**,**

P.O Box 43640-00100 Nairobi, Kenya.

mogero@kemri-wellcome.org

For peer review only

For peer review only - http://bmjopen.bmj.com/site/about/guidelines.xhtml

Abstract

Objectives: To identify and appraise the methodological rigor of multivariable prognostic models predicting in-hospital paediatric mortality in low- and middle-income countries (LMIC).

Design: Systematic review of peer-reviewed journals.

Data sources: MEDLINE, CINAHL, Google Scholar, and Web of Science electronic databases since inception to August 2019.

Eligibility criteria: We included model development studies predicting in-hospital paediatric mortality in LMIC.

Data extraction and synthesis: This systematic review followed the CHARMS (Checklist for critical Appraisal and data extraction for systematic Reviews of prediction Modelling Studies) framework. The risk of bias assessment was conducted using PROBAST (Prediction model Risk of Bias Assessment Tool). No quantitative summary was conducted due to substantial heterogeneity that was observed after assessing the studies included.

Results: Our search strategy identified a total of 4054 unique articles. Among these, 3545 articles were excluded after review of titles and abstracts as they covered non-relevant topics. Full texts of 509 articles were screened for eligibility, of which 15 studies reporting 21 models met the eligibility criteria. Based on the PROBAST tool, risk of bias was assessed in four domains; participant, predictors, outcome, and analyses. The domain of statistical analyses was the main area of concern where none of the included models was judged to be of low risk of bias.

Conclusion: This review identified 21 models predicting in-hospital paediatric mortality in LMIC. However, most reports characterising these models are of poor quality when judged against recent reporting standards due to a high risk of bias. Future studies should adhere to

For peer review only - http://bmjopen.bmj.com/site/about/guidelines.xhtml

standardized methodological criteria and progress from identifying new risk scores to validating or adapting existing scores.

Review registration number: CRD42018088599

Article summary

Strengths and limitations of this study

- This is the first review on methodological rigor of models predicting paediatric mortality in resource-limited settings.
- We used a robust search strategy with no language restriction yielding many potentially eligible studies.
- Due to substantial heterogeneity in the models included no meta-analyses was conducted.
- We relied on what was reported to determine the risk of bias in prognostic models included.
- Google Translate was used to translate one study from French to English.

For peer review only

Introduction

Over recent decades, there has been considerable progress in improving child survival[1] but child mortality remains high in sub-Saharan Africa relative to the rest of the world.[2] Paediatric deaths in hospitalized children mostly occur soon after admission,[3] and are caused by common conditions such as malaria, pneumonia, and diarrhoeal diseases among others, which are readily treatable by cost-effective interventions.[3-5] In low- and middle-income countries (LMIC), clinicians often use a set of clinical signs as recommended in the guidelines by World Health Organization (WHO) to identify patients at risk of deterioration while making decisions on appropriate treatment.[6] Clinical criteria recommended by WHO were developed following expert recommendations based on review of evidence from studies reporting risk factors for mortality. Prognostic/predictive models use statistical equations to predict high-risk patients based on the combination of risk factors. Use of these models by clinicians may improve patients' outcomes by enhancing clinicians' ability in identifying patients at the risk of deterioration.[7]

Several prognostic models for hospitalised children have been published over the last 3 decades,[8] however, there are doubts as to whether authors of these models used the appropriate methodology in their development.[9] Notably, in the current clinical practice guidelines, none of these models have been recommended for use in resource-limited setting and reviews of the methodology utilized in their development have been highly recommended.[10] This systematic review addresses this need and aims at identifying and summarizing existing studies reporting prognostic models or scoring systems predicting in-hospital paediatric mortality in LMIC. Specifically, the research summarises the evidence from the published studies and appraises the methodological rigor of each existing model.

For peer review only - http://bmjopen.bmj.com/site/about/guidelines.xhtml

Methods

Protocol and registration

As recommended, a research protocol for this review was published in a peer-reviewed journal,[11] and is also registered with the International Prospective Register of Systematic Reviews (PROSPERO) the registration number is CRD42018088599.[12] This study is reported as per guidelines by the Preferred Reporting Items for Systematic Reviews and Meta-Analyses (PRISMA).[13]

Eligibility criteria

We used the following eligibility criteria for inclusion of articles:

1. Study design: we included peer-reviewed studies whose study design was either a case-control, cohort (prospective or retrospective), cross-sectional, or randomized controlled trial.
2. Outcome: we included studies predicting all-cause in-hospital mortality. Studies predicting operative, trauma or post-discharge mortality were excluded.
3. Setting and target population: we focused on studies targeting over 1 month old children admitted in paediatric wards within resource-limited settings as specified by the World Bank[14]. Studies whose target population were children in HDU (High Dependency Unit) or ICU (Intensive Care Unit) were excluded because of limited availability of such facilities in LMIC. We also excluded studies whose target population included conditions not common in children, such as diabetes, cancer, chronic kidney disease, musculoskeletal disorders, etc. However, if a study focused on one of the common

For peer review only - http://bmjopen.bmj.com/site/about/guidelines.xhtml

childhood illnesses such as malaria, pneumonia, meningitis, anaemia, and diarrhoea/dehydration[3], then it was included.

4. Prognostic research studies: we included studies whose main objective was deriving a predictive model(s) or scoring system(s). We excluded case-series, conference proceedings, editorials, commentaries, expert views, case reports, reviews and studies that mainly generate hypothesis such as explanatory studies[15].
5. Predictors in the model: studies that reported multivariable model with at least 2 variables/predictors were included.
6. Full text and language: no language restrictions were made, we used Google Translate to translate non-English language studies. We excluded studies that were not available in full text.

Search strategy of articles

Based on CHARMS (Checklist for critical Appraisal and data extraction for systematic Reviews of prediction Modelling Studies) checklist[16], we identified seven core items (see supplementary file 1 Table 1) specific to our study that guided the formulation of the eligibility criteria, review aims, and the search strategy.

Where applicable, MeSH (Medical Subject Headlines) terms and keywords were used to identify research papers developing predictive models relevant for this review (see supplementary file 1 Table 2). We conducted a search of articles in CINAHL (via EbscoHost), Google Scholar, MEDLINE, and Web of Science published since inception to August 2019. To identify other potentially eligible studies, we manually searched reference lists of the identified articles and collated the final search results in EndNoteX7™ bibliography tool.

For peer review only - http://bmjopen.bmj.com/site/about/guidelines.xhtml

Screening of articles for inclusion

Prior to screening titles and abstracts, 2 reviewers (MO and LM) standardized the approach to be used in the process of screening and a sample of 30 articles were used to familiarize and train reviewers (MO, LM, and JA) on the process of screening of articles and data abstraction. Two reviewers (MO and LM) screened articles' titles and abstracts. Disagreements were resolved via discussion and the third reviewer (JA) adjudicated the final decision where necessary.

Data extraction from the included articles

For each of the study included, we used CHARMS guidelines to abstract the following data items; participant enrolment, study design, study population characteristics, location, sample size, number and selection of predictors, study dates, handling of continuous predictors, missing data, method of modelling (e.g. logistic regression, or survival), verification of model assumptions, internal validation methods (e.g. resampling techniques such as cross validations and bootstrapping, or random split of data); model presentation (e.g. full regression formula with coefficients, score chart, or nomogram); and model performance metrics including discrimination -area under the curve (AUC) accompanied with 95% CI (confidence intervals); calibration; classification metrics including specificity, sensitivity, positive, and negative predictive values. We further explored literature to determine if included models have been externally validated elsewhere. We treated each model separately for articles that developed multiple prognostic models. Data extracted from articles by the two reviewers (MO & LM) were compared and disagreements were resolved via discussion with the third reviewer (JA). Due to substantial heterogeneity that was observed after assessing studies included, we did not conduct a quantitative summary of the identified models.

For peer review only - http://bmjopen.bmj.com/site/about/guidelines.xhtml

Assessment of methodological rigor of the identified prognostic models

Based on PROBAST (Prediction study Risk Of Bias Assessment Tool) a Cochrane tool for assessing risk of bias(RoB) in predictive models,[17 18] we assessed the RoB for each model in four domains: selection of the study participant, predictors domain (e.g. selection of the candidate predictors), statistical analysis domain (e.g. sample size, continuous predictors, missing data), and outcome domain. See Table 3 in supplementary file 1 for details. In each domain there were a set of signalling questions each with five possible answers: yes; probably yes; probably no; no; and no information. Any positive answer (yes, or probably yes) suggests low RoB. There were three possible outcomes per domain namely: low; high; or unclear RoB. Using these outcomes, we came up with an overall rating of RoB for each model. As recommended by PROBAST, a prognostic model was rated to be of "low RoB" if all four domains had an outcome of "low". A prognostic model was rated "high RoB" if at least one domain had an outcome of "high". Finally, a prognostic model was rated as "unclear RoB" if at least one domain had an outcome of "unclear" and the rest of the domains had an outcome of "low".

Patient and public involvement

No patient or public involvement.

For peer review only - http://bmjopen.bmj.com/site/about/guidelines.xhtml

Results

Characteristics of the included studies

Our search strategy identified a total of 4054 unique articles, 3545 articles were excluded after review of titles and abstracts as they reported non-relevant topics. Full texts of 509 articles were assessed for eligibility, of which 15 primary studies reporting 21 developed models met the eligibility criteria (Figure 1). The eligible studies analysed data for patients who were below 15 years of age with median mortality being 6.7% (range 1.2% to 43.9%).[19 20] While majority of the models were developed for general cases in paediatric wards (n=9), some were tailored for specific paediatric groups defined by common diagnoses such as febrile illness (n=1),[21] malaria (n=2), [22 23] pneumonia (n=4),[19 24-26] malnutrition (n=2) [27 28] and other infectious diseases (n=3) (see supplementary file 2).

Most of the included studies have been published post year 2000 (n = 20) except for one study[27] published in 1996. The latest data used in the models under review were from 2016 to 2017 by Rosman *et al.*[29] and the oldest data were utilized by Draimax *et al.*[27] from 1986 to 1988.

Five reports of the 15 included studies utilized data from at least two hospitals of which 3 studies[21 22 26] were conducted in multiple countries including sub-Saharan Africa and Asian countries (Figure 2). Of the reviewed studies, most of the information we were abstracting were either not reported or were partially reported, an indication of non-adherence to the Transparent Reporting of a Multivariable Prognostic Model for Individual Prognosis or Diagnosis guidelines (TRIPOD).[30 31]

For peer review only - http://bmjopen.bmj.com/site/about/guidelines.xhtml

Summary of issues in methodology of the reviewed models

Candidate predictors

There were 61 distinct predictors used in the final reported models with a median of 7 predictors in any one model. Initial selection of the independent candidate predictors was mostly based on univariable analyses except for three studies[21 25 27] where the selection was based on literature reviews or clinical relevance. Backward stepwise selection method was used in 6 models in a multivariable analysis to determine final model predictors. Commonly included predictors in the final models included altered consciousness, malnutrition indicators, vital signs, and signs of respiratory distress (see Figure 3). Some models included predictors that were either not easy to obtain or required laboratory techniques. Of the 13 models that used continuous predictors, 8 models categorized these continuous predictors where a continuous scale would have been possible. Two out of 13 models applied other techniques such as fractional polynomial[21] and restricted cubic splines[28] to determine the suitable functional form of the continuous predictors (see supplementary file 2).

Sample size, events per variable (EPV) and missing data

Sample size ranged from 168[29] to 50249[32] with a median of 1307. The median EPV was 21(IQR 8.3 – 32.5) of which 7 models had less than 10 EPVs, suggestive of insufficient sample sizes which is prone to over-fitting. For instance, 60 deaths were reported in the dataset used to develop *PEDIA-Immediate* score in the study by Berkley *et al*. In reference to the rule that a study developing a predictive model should have a minimum of 10 events (deaths) for each independent candidate predictor in a predictive model,[33] a model with a maximum of 6

For peer review only - http://bmjopen.bmj.com/site/about/guidelines.xhtml

predictors should have been considered but 10 predictors were considered instead hence making EPV to be 6.

Proportions of missing data was not always reported. Handling of missing data varied across the reviewed studies as follows: 6 models did not report handling of missing data; 8 used complete case analysis; 4 used multiple imputations by chained equations; and one study[28] used single imputation.

Model development

Majority of the studies applied logistic regression, one study[21] used Cox regression, one study[20] used Spiegelhalter/Knill-Jones method, and another study[23] used a machine learning technique (classification and regression trees) in model development. Verification of model assumptions was not reported in most of the studies. For instance, George *et al*[21] despite utilizing Cox regression to develop their model, did not report the verification of proportional hazard assumption nor explore the possibility of competing risks as recommended[34]. Other regression assumptions e.g. multicollinearity was equally not reported. However, since backward elimination method disregards redundant variables, we inferred the satisfaction of multicollinearity assumption if this method was applied.[35] Five studies developed models using data from different countries/centres but none of them clustered their analysis by source of data in a multilevel model to account for heterogeneity. Ignoring clustering leads to a biased predictor effect.[36]

Model performance evaluation & presentation

Performance measures (both calibration and discrimination) were poorly reported in most of the studies and in most cases (n=20) AUC for discrimination was reported. Performance of the derived models was evaluated in 12 models using either split-sample, resampling methods, or

For peer review only - http://bmjopen.bmj.com/site/about/guidelines.xhtml

For peer review only

separate datasets. Except for the model derived by George *et al,*[21] all other models did not report both apparent discrimination (without any adjustment for optimism) and optimism-corrected discrimination measures. Despite inadequate reporting of the models' performance, 16 models reported AUCs ≥ 0.80, an indication of promising models. Apart from the following exceptions; *Lambarene Organ Dysfunction* (LOD) score,[22] *Paediatric Early Death Index for Africa* (PEDIA) score,[20] *Signs of Inflammation in Children that Kill* (SICK) score,[37] *Respiratory Index of Severity in Children*(RISC) score,[19] and *Modified Respiratory Index of Severity in Children* (mRISC) score,[24] other prognostic models in this review have not been externally validated (by independent investigators using diverse populations). Only 2 studies[25 38] developing 4 models provided a full model formula (both coefficients and intercept/baseline function) in their results as recommended.[30 31] While most of the models (n=17) were presented as simplified integer scores, only a few were assigned weights according to the regression coefficients.

Risk of bias (RoB)

Based on the PROBAST tool, RoB was assessed in four domains; participants, predictors, outcome, and analyses. Figure 4 summarizes the RoB assessment across all models included in this review where the domain of outcome was deemed to be of low RoB in all models. The domain of statistical analyses was the main area of concern where 19 out of 21 models did not report comprehensive details of model development as expected to warrant a proper risk of bias assessment using the 9 signalling questions under the analyses domain. As a result, these models were judged to be of unclear RoB under this domain (see Figure 5). Details on how models were scored against each of the PROBAST criterion (20 signalling questions) across the four domains are provided in the supplementary file 3. In the overall judgement of RoB, 9 out of 21 models were judged to be of high risk of bias because at least one out of four domains in these models

For peer review only - http://bmjopen.bmj.com/site/about/guidelines.xhtml

were rated as high RoB. The remaining models (12/21) were judged to be of unclear RoB on account of being rated low and unclear RoB in the domains. No model was rated low RoB in all four domains.

Discussion

Summary of key findings

We conducted a systematic review to identify scores predicting in-hospital mortality for paediatrics in resource-limited countries. Fifteen studies that described the development of 21 prognostic models were identified. We describe characteristics of these studies as well as the methodological quality of the included models by using agreed recent guidelines applicable to predictive models. We have identified several important quality deficiencies such as inadequate reporting and other methodological concerns, including poor handling of missing data, automated selection of predictors, categorization of continuous predictors, inadequate EPV and the poor presentation of the proposed model for use. As a result, no model was found to be of good methodological quality and consequently judged to be potentially high or unclear risk of bias in predictions (Figure 5).

Our findings suggest that predictive models fail to meet recently agreed methodological criteria in various ways. Firstly, in this review we observed that univariable analysis was routinely used in 18 out of 21 models in the selection of candidate predictors to be used in a multivariable analysis. This strategy tends to leave out possibly important prognostic factors which might be insignificant in a univariable analysis but turn out to be significant when combined with other predictors.[30 31] *A priori* selection of predictors using expert opinion, clinical intuition or literature is recommended for this purpose,[39 40] however only three studies in this review employed this approach.[21 25 27]

For peer review only - http://bmjopen.bmj.com/site/about/guidelines.xhtml

Small sample sizes in model development can lead to poor predictive performance, over-fitting, and biased effect estimates. Prognostic models must have a minimum of ten events per candidate independent predictor, as this is the accepted norm[41-43] and underpowered models arising from inadequate events per variable (EPV) increases the possibility of spurious results.[33] In this review, 7/21 models had inadequate sample sizes (EPV<10) and there was no information on whether bootstrapping, which serves to reduce overfitting was used in these models.[44]

Just like most of the epidemiological studies, missing data is a common problem which is solved using multiple imputation or other appropriate approaches, but this was rarely the case in the model development studies under this review. For instance, 8/21 models used Complete Case Analysis (CCA), 4/21 used multiple imputation under the MAR (Missing at Random) assumption, and 6/21models did not report handling of missing data and therefore we assumed CCA was used. Following Harrell's guidelines,[45] CCA should only be used if the percentage of missingness is < 5% but the appropriateness of the CCA approach could not be ascertained as most of the included studies failed to report the proportion of missing data per variable. Inappropriate use of CCA results in use of only a small subset of the data which cannot be regarded as a random sample from the target population unless data are MCAR (Missing Completely At Random),[46] a mechanism which is rare in practice.[47] Consequently, there are concerns about possible loss of precision in inferences and the potential biases of the estimated parameters[48] in the models employing CCA. While Multiple Imputation by Chained Equations (MICE) is the principled method of imputing missing data, implementing this method when the data are not missing at random could result in biased model quantities.[49] As a result, sensitivity analyses of the resultant imputations is recommended to investigate the departure from MAR assumption.[50] However, this was not the case in the studies that performed imputations on their

For peer review only - http://bmjopen.bmj.com/site/about/guidelines.xhtml

data. Finally, handling of continuous predictors was also a concern in this review. Of the 13 models including continuous predictors, 8 models[19 20 24-27 37 51] categorized continuous predictors where a continuous scale would have been possible. While this approach is intuitive to most researchers, its simplicity comes at a considerable cost of predictive performance.[52] The resulting prognostic models have been shown to have poor predictive accuracy because of the loss of statistical power and information. It is recommended that the nature of continuous data should be reserved or be handled by appropriate techniques e.g. flexible parametrizations such as fractional polynomial, regression splines, or apply non-parametric techniques such as locally estimated scatterplot smoothing (LOESS) functions.[52 53] In this review, appropriate methods of transforming continuous data was done by only 2 studies[21 28] which applied restricted cubic splines and fractional polynomial.

Sixteen models attained the discrimination metric of above 80%, an indicator of promising models. However, given that the median mortality of the included studies was 6.7%, the performance reported should be interpreted with caution on account of heavily imbalanced data as a result of the rare nature of the outcome of interest. For instance, in a study with a mortality rate of 5%, a model predicting no deaths could easily attain 95% accuracy which could be potentially misleading[34 54]. Therefore, authors should report additional measures of model performance such as model specificity, sensitivity, accuracy, positive and negative predictive values for models to be contextualized appropriately.

For peer review only - http://bmjopen.bmj.com/site/about/guidelines.xhtml

Comparison with Other Studies

Methods used to assess quality measures of the included models in the current study have been applied previously to critically evaluate the quality of predictive models in other specialties.[55-57] Just like the findings of this review, other previous reviews [9 58-60] describing the development of prognostic models highlighted many flaws including inappropriate statistical analyses, poor reporting of key methodological information necessary for model validation, and lack of external validations in general. Detailed and transparent reporting of the methods used in model development is one of the core principles of integrity in research because this is the only way the research community is able to evaluate study findings, and the assessment of risk of bias.[61] Incomplete reporting of clinical models limits future studies on prognostic research from building on the information of already existing models. This has been marked as an important source of wasted research efforts.[62] For example, external validation of prognostic models requires a full model formula to enable direct estimation of survival probabilities.[31] However, this was presented in only 4 models. Five models [19 20 22 24 37] that were reported to have undergone external validation did not report full model formula as required. It is therefore not clear whether authors of these external validation studies applied model coefficients to the external datasets, or they estimated new model coefficients (essentially model redevelopment). Thus, this review highlights the need for researchers to adhere to the TRIPOD guidelines that were created to help authors of prognostic models write complete and transparent reports. Of note, the quality of clinical predictive models does not appear to have improved over time as previous reviews from 1996,[63] 1997,[64] 2001,[65] 2005,[66] 2011,[8] 2012,[67] 2016,[68 69] 2017, [70] to 2019[71] have consistently identified suboptimal methodologies in the development of the score/predictive models especially in the domain of analysis. Poorly derived models may result

in overoptimistic results and misleading performances. Presumably there are reasons why many prognostic models are of poor quality, including pressure to publish new predictive model regardless of the clinical value of the resultant model[72], and inadequate biostatistical support to investigators. As observed by one of the reviewers of this study, some of the issues identified in this review such as absence of the details on the model development process can be corrected during the review and the editorial process by the journals publishing the work. There is therefore a role for editorial process for promoting best practices and recommendations of developing predictive models stated in the TRIPOD statement and ensuring compliance by authors as part of checklist for submission.

Implications of this study

Prognostic model development pipeline include development, validation (internal and external), impact assessment and implementation. Most of the included models are still in the first step of the pipeline. This suggests that researchers focus more on deriving new models, often using similar prognostic factors, rather than validating and improving existing prognostic models. This leaves healthcare policy makers with doubts as to which model to recommend in their setting. It is now time to move the prognostic research to the next step (external validation). Large patient-level datasets such as that of the Clinical Information Network (CIN)[3] which has been collected over time from a number of referral hospitals now exist in Kenya and it has been used to answer a number of salient clinical questions relevant across a range of resource-limited setting[73-75]. Future studies on prognostic research should leverage such datasets to externally validate competing models identified in this review for comparative performances as recommended by Collins *et al,*[76] and if necessary, predictive performance of such models should be improved by addition of new prognostic factors. We also noted that most of the included models simplified

For peer review only - http://bmjopen.bmj.com/site/about/guidelines.xhtml

the original predictor coefficients by rounding them to a nearest integer. This practice has an implication on model performance during external validation due to loss in predictive accuracy arising from rounding coefficients to nearest integers.[47]

We now provide guidance on methodological concerns about the candidate predictors as noted in this review. While considering potential candidate predictors to include in the prediction model, researchers should focus on the predictors that will be available at the time the prediction is made. We acknowledge that some predictors obtained from invasive procedures e.g. C-reactive protein, blood gas analyses, blood or cerebrospinal fluid culture, etc might have a higher predictive value for mortality compared to predictors derived from subjective clinical assessments, however in resource-limited settings results of such laboratory tests typically take days to be reported or resources might not available to perform such tests in many hospitals. Consequently, models utilising such variables might not be useful to clinicians to make a decision at typical emergency departments in LMIC. Screening of model candidate predictors based on the bivariate associations whereby predictors are selected if they meet some *p*-value threshold (commonly 0.05) have been strongly discouraged previously[77 78]. Categorising continuous model predictors is a common practice by researchers however this practice discards a lot of information and its assumptions are rarely clinically plausible.[34] Finally, there is a risk of overfitting if the model includes more predictors than the dataset can support. The ratio of the events (deaths) to the number of independent candidate predictors have been discussed extensively in methodological papers elsewhere[79 80] and it has been recommended that ratio of the EPV should be at least 10.

For peer review only - http://bmjopen.bmj.com/site/about/guidelines.xhtml

Strengths and limitations

To our knowledge, this is the first systematic review identifying prognostic models and scoring systems predicting in-hospital all-cause paediatric mortality in low-and-middle income settings. Our robust search strategy yielded a number of potentially eligible studies, hence it is unlikely that any potentially eligible study was not included. The quality of included models was assessed based on recent reporting standards and applied to the identified studies. For instance, if no mention was made of internal validation or even verification of the model assumptions, we could determine whether these crucial steps of model development were carried out or not. Thus, models that could have been otherwise rated as low risk of bias were rated as either unclear or high risk of bias in each domain. The PROBAST's analysis domain has most (9 out of 20) of the signalling questions and any given model in this domain had much higher chance to be defined as high risk as long as there was one negative (no or probably no) answer. This strict criterion led to all models being classified as either unclear or high risk of bias and therefore metanalysis was not performed. We acknowledge that if we somewhat relaxed this decision rule, our conclusion could change. Despite this, we still hold that authors should adhere to guidelines of transparent and complete reporting of any proposed prognostic model to facilitate its external validation and subsequent application in practice. Finally, we used Google Translate to interpret a study by Bitwe et al[51] from French to English. It is possible that some statistical terminologies were not rendered correctly, or some model characteristics were lost in translation.

Conclusion

Rigorously developed and robustly validated promising predictive models have the potential for improving child survival in resource-limited countries. This review identified models predicting in-hospital mortality for paediatrics. However, none of them is of good quality. Our research

For peer review only - http://bmjopen.bmj.com/site/about/guidelines.xhtml

highlights the need to improve on the identified quality deficiencies when developing prognostic models in the future by adhering to existing generally accepted standardized methodological criteria. Majority of the derived models have not been externally validated as required. Inadequate reporting observed in the included models hinders rigorous external validation by other researchers in addition to undermining their application. Rather than developing new prognostic models, researchers should carry out comprehensive joint external validation of the identified models using large datasets ideally collected over extended time periods and different locations. This will allow model comparisons and adaptation of the competing models, if necessary, to ascertain their generalizability.

Funding

Funds from The Wellcome Trust (#207522) awarded to ME as a Senior Fellowship together with additional funds from a Wellcome Trust core grant awarded to the KEMRI-Wellcome Trust Research Programme (#092654 and #203077) supported this work. SA was supported by the Initiative to Develop African Research Leaders (IDeAL) Wellcome Trust award (#107769). The funders had no role in drafting or submitting this manuscript.

Author Statement

The roles of the contributors were as follows: ME, SA, and AA conceptualized the study. MO, LM and JA conducted electronic searches to identify eligible models and did analyses. MO drafted the initial manuscript with SA, NO, RS, AA, and ME contributed to its development. All authors read and approved the final manuscript.

Competing interests

The authors declare no conflicts of interest.

For peer review only - http://bmjopen.bmj.com/site/about/guidelines.xhtml

Data availability statement

All data are provided within the manuscript and supplementary files.

For peer review only

For peer review only - http://bmjopen.bmj.com/site/about/guidelines.xhtml

References

1. World Health Organization. Child mortality rates plunge by more than half since 1990 but global MDG target missed by wide margin: WHO; 2015 [Available from: http://www.who.int/mediacentre/news/releases/2015/child-mortality-report/en/ accessed 20 January 2018.
2. Lucia Hug DS, Danzhen You. Levels and Trends in Child Mortality: UNICEF; 2019 [Available from: https://data.unicef.org/resources/levels-and-trends-in-child-mortality/ accessed August 2019.
3. Ayieko P, Ogero M, Makone B, et al. Characteristics of admissions and variations in the use of basic investigations, treatments and outcomes in Kenyan hospitals within a new Clinical Information Network. *Archives of disease in childhood* 2015:archdischild-2015-309269.
4. World Health Organization. Serious childhood problems in countries with limited resources. Geneva2004.
5. World Health Organization. Children: reducing mortality: WHO; 2017 [Available from: http://www.who.int/mediacentre/factsheets/fs178/en/ accessed 20 January 2018.
6. World Health Organization. Handbook IMCI: integrated management of childhood illness. Geneva2005.
7. Vogenberg FR. Predictive and prognostic models: implications for healthcare decision-making in a modern recession. *American health & drug benefits* 2009;2(6):218.
8. Maguire JL, Kulik DM, Laupacis A, et al. Clinical prediction rules for children: a systematic review. *Pediatrics* 2011;128(3):e666-e77. doi: 10.1542/peds.2011-0043
9. Bouwmeester W, Zuithoff NP, Mallett S, et al. Reporting and methods in clinical prediction research: a systematic review. *PLoS medicine* 2012;9(5):e1001221.
10. Altman DG. Systematic reviews in health care: Systematic reviews of evaluations of prognostic variables. *BMJ: British Medical Journal* 2001;323(7306):224.
11. Ogero M, Sarguta R, Malla L, et al. Methodological rigor of prognostic models for predicting in-hospital paediatric mortality in low-and middle-income countries: a systematic review protocol. *Wellcome Open Research* 2020;5(106):106.
12. Ogero M, Malla L, Agweyu A, et al. Prognostic models predicting in-hospital mortality of paediatrics in resource-limited countries: systematic review. 2018
13. Moher D, Liberati A, Tetzlaff J, et al. Preferred reporting items for systematic reviews and meta-analyses: the PRISMA statement. *PLoS medicine* 2009;6(7):e1000097.
14. WORLD BANK. World Bank Country and Lending Groups: WOLD BANK; 2019 [Available from: https://datahelpdesk.worldbank.org/knowledgebase/articles/906519-world-bank-country-and-lending-groups accessed September 2019.
15. Shmueli G. To explain or to predict? *Statistical science* 2010:289-310.
16. Moons KG, de Groot JA, Bouwmeester W, et al. Critical appraisal and data extraction for systematic reviews of prediction modelling studies: the CHARMS checklist. *PLoS medicine* 2014;11(10)
17. Wolff RF, Moons KG, Riley RD, et al. PROBAST: a tool to assess the risk of bias and applicability of prediction model studies. *Annals of internal medicine* 2019;170(1):51-58.
18. Moons KG, Wolff RF, Riley RD, et al. PROBAST: a tool to assess risk of bias and applicability of prediction model studies: explanation and elaboration. *Annals of internal medicine* 2019;170(1):W1-W33.
19. Reed C, Madhi SA, Klugman KP, et al. Development of the Respiratory Index of Severity in Children (RISC) score among young children with respiratory infections in South Africa. *Plos One* 2012;7(1):e27793-e93. doi: 10.1371/journal.pone.0027793

For peer review only - http://bmjopen.bmj.com/site/about/guidelines.xhtml

20. Berkley JA, Ross A, Mwangi I, et al. Prognostic indicators of early and late death in children admitted to district hospital in Kenya: cohort study. *BMJ (Clinical Research Ed)* 2003;326(7385):361-61.
21. George EC, Walker AS, Kiguli S, et al. Predicting mortality in sick African children: the FEAST Paediatric Emergency Triage (PET) Score. *BMC Medicine* 2015;13(1):1-12. doi: 10.1186/s12916-015-0407-3
22. Helbok R, Kendjo E, Issifou S, et al. The Lambaréné Organ Dysfunction Score (LODS) is a simple clinical predictor of fatal malaria in African children. *Journal of Infectious Diseases* 2009;200(12):1834-41. doi: 10.1086/648409
23. Erdman LK, Dhabangi A, Musoke C, et al. Combinations of host biomarkers predict mortality among Ugandan children with severe malaria: a retrospective case-control study. *Plos One* 2011;6(2):e17440-e40. doi: 10.1371/journal.pone.0017440
24. Emukule GO, McMorrow M, Ulloa C, et al. Predicting mortality among hospitalized children with respiratory illness in Western Kenya, 2009-2012. *Plos One* 2014;9(3):e92968-e68. doi: 10.1371/journal.pone.0092968
25. Hooli S, Colbourn T, Lufesi N, et al. Predicting Hospitalised Paediatric Pneumonia Mortality Risk: An External Validation of RISC and mRISC, and Local Tool Development (RISC-Malawi) from Malawi. *PLoS ONE* 2016;11(12):1-13. doi: 10.1371/journal.pone.0168126
26. Gallagher KE, Knoll MD, Prosperi C, et al. The Predictive Performance of a Pneumonia Severity Score in Human Immunodeficiency Virus–negative Children Presenting to Hospital in 7 Low-and Middle-income Countries. 2019
27. Dramaix M, Brasseur D, Donnen P, et al. Prognostic indices for mortality of hospitalized children in central Africa. *American Journal Of Epidemiology* 1996;143(12):1235-43.
28. Olson D, Davis NL, Milazi R, et al. Development of a severity of illness scoring system (inpatient triage, assessment and treatment) for resource-constrained hospitals in developing countries. *Tropical Medicine & International Health* 2013;18(7):871-78. doi: 10.1111/tmi.12137
29. Rosman SL, Karangwa V, Law M, et al. Provisional validation of a pediatric early warning score for resource-limited settings. *Pediatrics* 2019;143(5):e20183657.
30. Collins GSP, Reitsma JBMDP, Altman DGD, et al. Transparent Reporting of a multivariable prediction model for Individual Prognosis Or Diagnosis (TRIPOD): The TRIPOD Statement. *Annals of Internal Medicine* 2015;162(1):55-63.
31. Moons KGMP, Altman DGD, Reitsma JBMDP, et al. Transparent Reporting of a multivariable prediction model for Individual Prognosis Or Diagnosis (TRIPOD): Explanation and Elaboration. *Annals of Internal Medicine* 2015;162(1):W1-W73.
32. Mpimbaza A, Sears D, Sserwanga A, et al. Admission Risk Score to Predict Inpatient Pediatric Mortality at Four Public Hospitals in Uganda. *Plos One* 2015;10(7):e0133950-e50. doi: 10.1371/journal.pone.0133950
33. Concato J, Feinstein AR, Holford TR. The risk of determining risk with multivariable models. *Annals of internal medicine* 1993;118(3):201-10.
34. Leisman DE, Harhay MO, Lederer DJ, et al. Development and reporting of prediction models: guidance for authors from editors of respiratory, sleep, and critical care journals. *Critical care medicine* 2020;48(5):623.
35. Vijay Kotu BD. Feature Selection. In: Vijay Kotu BD, ed. Data Science Concepts and Practice2019:467-90.
36. Debray T, Moons KG, Ahmed I, et al. A framework for developing, implementing, and evaluating clinical prediction models in an individual participant data meta-analysis. *Statistics in medicine* 2013;32(18):3158-80.
37. Kumar N, Thomas N, Singhal D, et al. Triage score for severity of illness. *Indian pediatrics* 2003;40(3):204-10.

For peer review only - http://bmjopen.bmj.com/site/about/guidelines.xhtml

38. Lowlaavar N, Larson CP, Kumbakumba E, et al. Pediatric in-Hospital Death from Infectious Disease in Uganda: Derivation of Clinical Prediction Models. *Plos One* 2016;11(3):e0150683-e83. doi: 10.1371/journal.pone.0150683
39. Justice AC, Covinsky KE, Berlin JA. Assessing the generalizability of prognostic information. *Annals of internal medicine* 1999;130(6):515-24.
40. Kattan MW, Harrell FE. We should not be so quick to abandon the use of domain experts and full models (letter commenting: J Clin Epidemiol. 2015; 71C: 76--85.). *Journal of clinical epidemiology* 2016;75:131.
41. Concato J, Peduzzi P, Holford TR, et al. Importance of events per independent variable in proportional hazards analysis I. Background, goals, and general strategy. *Journal of clinical epidemiology* 1995;48(12):1495-501.
42. Peduzzi P, Concato J, Feinstein AR, et al. Importance of events per independent variable in proportional hazards regression analysis II. Accuracy and precision of regression estimates. *Journal of clinical epidemiology* 1995;48(12):1503-10.
43. Peduzzi P, Concato J, Kemper E, et al. A simulation study of the number of events per variable in logistic regression analysis. *Journal of clinical epidemiology* 1996;49(12):1373-79.
44. A bootstrapping approach to reduce over-fitting in genetic programming. Proceedings of the 15th annual conference companion on Genetic and evolutionary computation; 2013. ACM.
45. Harrell FE. Regression Modeling Strategies. *BIOS* 2014;330
46. White IR, Royston P, Wood AM. Multiple imputation using chained equations: Issues and guidance for practice. *Stat Med* 2011;30(4):377-99. doi: 10.1002/sim.4067
47. Moons KG, de Groot JA, Bouwmeester W, et al. Critical appraisal and data extraction for systematic reviews of prediction modelling studies: the CHARMS checklist. *PLoS medicine* 2014;11(10):e1001744.
48. Molenberghs G, Fitzmaurice G, Kenward MG, et al. Handbook of missing data methodology: CRC Press 2014.
49. Azur MJ, Stuart EA, Frangakis C, et al. Multiple imputation by chained equations: what is it and how does it work? *International journal of methods in psychiatric research* 2011;20(1):40-49.
50. Gachau S, Quartagno M, Njagi EN, et al. Handling missing data in modelling quality of clinician-prescribed routine care: Sensitivity analysis of departure from missing at random assumption. *Statistical Methods in Medical Research* 2020:0962280220918279.
51. Bitwe R, Dramaix M, Hennart P. Simplified prognostic model of overall intrahospital mortality of children in central Africa. *Tropical medicine & international health: TM & IH* 2006;11(1):73.
52. Collins GS, Ogundimu EO, Cook JA, et al. Quantifying the impact of different approaches for handling continuous predictors on the performance of a prognostic model. *Statistics in medicine* 2016;35(23):4124-35.
53. Royston P, Altman DG, Sauerbrei W. Dichotomizing continuous predictors in multiple regression: a bad idea. *Statistics in medicine* 2006;25(1):127-41.
54. Leisman DE. Rare events in the ICU: An emerging challenge in classification and prediction. *Critical Care Medicine* 2018;46(3):418-24.
55. Ensor J, Riley RD, Moore D, et al. Systematic review of prognostic models for recurrent venous thromboembolism (VTE) post-treatment of first unprovoked VTE. *BMJ open* 2016;6(5):e011190.
56. Smit HA, Pinart M, Antó JM, et al. Childhood asthma prediction models: a systematic review. *The Lancet Respiratory Medicine* 2015;3(12):973-84. doi: 10.1016/S2213-2600(15)00428-2
57. Hodgson LE, Sarnowski A, Roderick PJ, et al. Systematic review of prognostic prediction models for acute kidney injury (AKI) in general hospital populations. *BMJ open* 2017;7(9):e016591.

For peer review only - http://bmjopen.bmj.com/site/about/guidelines.xhtml

58. Collins GS, de Groot JA, Dutton S, et al. External validation of multivariable prediction models: a systematic review of methodological conduct and reporting. *BMC medical research methodology* 2014;14(1):40.
59. Collins GS, Omar O, Shanyinde M, et al. A systematic review finds prediction models for chronic kidney disease were poorly reported and often developed using inappropriate methods. *Journal of clinical epidemiology* 2013;66(3):268-77.
60. Fahey M, Crayton E, Wolfe C, et al. Clinical prediction models for mortality and functional outcome following ischemic stroke: A systematic review and meta-analysis. *PloS one* 2018;13(1):e0185402.
61. Logullo P, MacCarthy A, Kirtley S, et al. Reporting guideline checklists are not quality evaluation forms: they are guidance for writing. *Health Science Reports* 2020;3(2)
62. Glasziou P, Altman DG, Bossuyt P, et al. Reducing waste from incomplete or unusable reports of biomedical research. *The Lancet* 2014;383(9913):267-76.
63. Kwakkel G, Wagenaar RC, Kollen BJ, et al. Predicting disability in stroke—a critical review of the literature. *Age and ageing* 1996;25(6):479-89.
64. Laupacis A, Sekar N. Clinical prediction rules: a review and suggested modifications of methodological standards. *Jama* 1997;277(6):488-94.
65. Counsell C, Dennis M. Systematic review of prognostic models in patients with acute stroke. *Cerebrovascular diseases* 2001;12(3):159-70.
66. Jacob M, Lewsey JD, Sharpin C, et al. Systematic Review and Validation of Prognostic Models in Liver Transplantation. *Liver Transplantation* 2005;11(7):814-25.
67. Moons KGM, Kengne AP, Grobbee DE, et al. Risk prediction models: II. External validation, model updating, and impact assessment. *Heart* 2012;98(9):691-98.
68. Damen JA, Hooft L, Schuit E, et al. Prediction models for cardiovascular disease risk in the general population: systematic review. *Bmj* 2016;353:i2416.
69. Cruz GD, Strathe AB, Rossow HA, et al. Characterizing bovine adipocyte distribution and its relationship with carcass and meat characteristics using a finite mixture model. *J Anim Sci* 2012;90(9):2995-3002. doi: 10.2527/jas.2011-4838
70. Singendonk MMJ, Tabbers MM, Benninga MA, et al. Pediatric Gastroesophageal Reflux Disease: Systematic Review on Prognosis and Prognostic Factors. *Journal Of Pediatric Gastroenterology And Nutrition* 2017 doi: 10.1097/MPG.0000000000001697
71. Carrillo-Larco RM, Altez-Fernandez C, Pacheco-Barrios N, et al. Cardiovascular Disease Prognostic Models in Latin America and the Caribbean: A Systematic Review. *Global heart* 2019;14(1):81-93.
72. Vickers AJ, Cronin AM. Everything you always wanted to know about evaluating prediction models (but were too afraid to ask). *Urology* 2010;76(6):1298-301.
73. Irimu G, Ogero M, Mbevi G, et al. Approaching quality improvement at scale: a learning health system approach in Kenya. *Archives of disease in childhood* 2018;103(11):1013-19.
74. Ogero M, Akech S, Malla L, et al. Examining which clinicians provide admission hospital care in a high mortality setting and their adherence to guidelines: an observational study in 13 hospitals. *Archives of Disease in Childhood* 2020
75. Ogero M, Ayieko P, Boniface Makone TJ, et al. An observational study of monitoring of vital signs in children admitted to Kenyan hospitals: an insight into the quality of nursing care? *Journal of global health* 2018;8(1)
76. Collins GS, Moons KG. Comparing risk prediction models: British Medical Journal Publishing Group, 2012.
77. Sun G-W, Shook TL, Kay GL. Inappropriate use of bivariable analysis to screen risk factors for use in multivariable analysis. *Journal of clinical epidemiology* 1996;49(8):907-16.

For peer review only - http://bmjopen.bmj.com/site/about/guidelines.xhtml

78. Wasserstein RL, Lazar NA. The ASA statement on p-values: context, process, and purpose: Taylor & Francis, 2016.
79. Austin PC, Allignol A, Fine JP. The number of primary events per variable affects estimation of the subdistribution hazard competing risks model. *Journal of clinical epidemiology* 2017;83:75-84.
80. Austin PC, Steyerberg EW. Events per variable (EPV) and the relative performance of different strategies for estimating the out-of-sample validity of logistic regression models. *Statistical methods in medical research* 2017;26(2):796-808.

Captions

Figure 1: PRISMA flow diagram showing the process used to identify prognostic models predicting in-hospital paediatric mortality included in this review.

Figure 2: Prognostic models predicting in-hospital paediatric mortality identified by country. Text highlighted in red are the names of the models with their corresponding discrimination measures (area under the curve). Key: PEWS-RL score=Paediatric Early Warning Score for Resource-Limited Settings; SICK score=Signs of Inflammation in Children that Kill; PET score= Paediatric Emergency Triage; mRISC score= Modified Respiratory Index of Severity in Children score; RISC score= Respiratory Index of Severity in Children score; PERCH severity score= Pneumonia Etiology Research for Child Health severity score; LOD score= Lambarene Organ Dysfunction score; CRT= Classification and Regression Trees; ITAT Score= Inpatient Triage Assessment and Treatment score; PEDIA score= Paediatric Early Death Index for Africa score.

Figure 3: Top four categories of predictors in the models of the reviewed reports: altered consciousness (coma, prostration, not alert, unconscious); malnutrition indicators (kwashiorkor, edema, weight-for-height z-score, weight-for-age z-score, mid-upper arm circumference-MUAC, wasting); vital signs (temperature, respiratory rate, heart rate, oxygen saturation); signs of respiratory distress (indrawing, lung crepitation, difficult breathing, grunting).

For peer review only - http://bmjopen.bmj.com/site/about/guidelines.xhtml

Figure 4: Summary of the risk of bias of the included models using PROBAST (Prediction study Risk of Bias Assessment Tool).

Figure 5: Risk of bias assessment. Low means low risk of bias, High means a high risk of bias, and Unclear bias means it was not possible to assess the risk of bias. Key: PEWS-RL score=Paediatric Early Warning Score for Resource-Limited Settings; SICK score=Signs of Inflammation in Children that Kill; PET score= Paediatric Emergency Triage; mRISC score= Modified Respiratory Index of Severity in Children score; RISC score= Respiratory Index of Severity in Children score; PERCH severity score= Pneumonia Etiology Research for Child Health severity score; LOD score= Lambarene Organ Dysfunction score; CRT= Classification and Regression Trees; ITAT Score= Inpatient Triage Assessment and Treatment score; PEDIA score= Paediatric Early Death Index for Africa score.

For peer review only

For peer review only - http://bmjopen.bmj.com/site/about/guidelines.xhtml

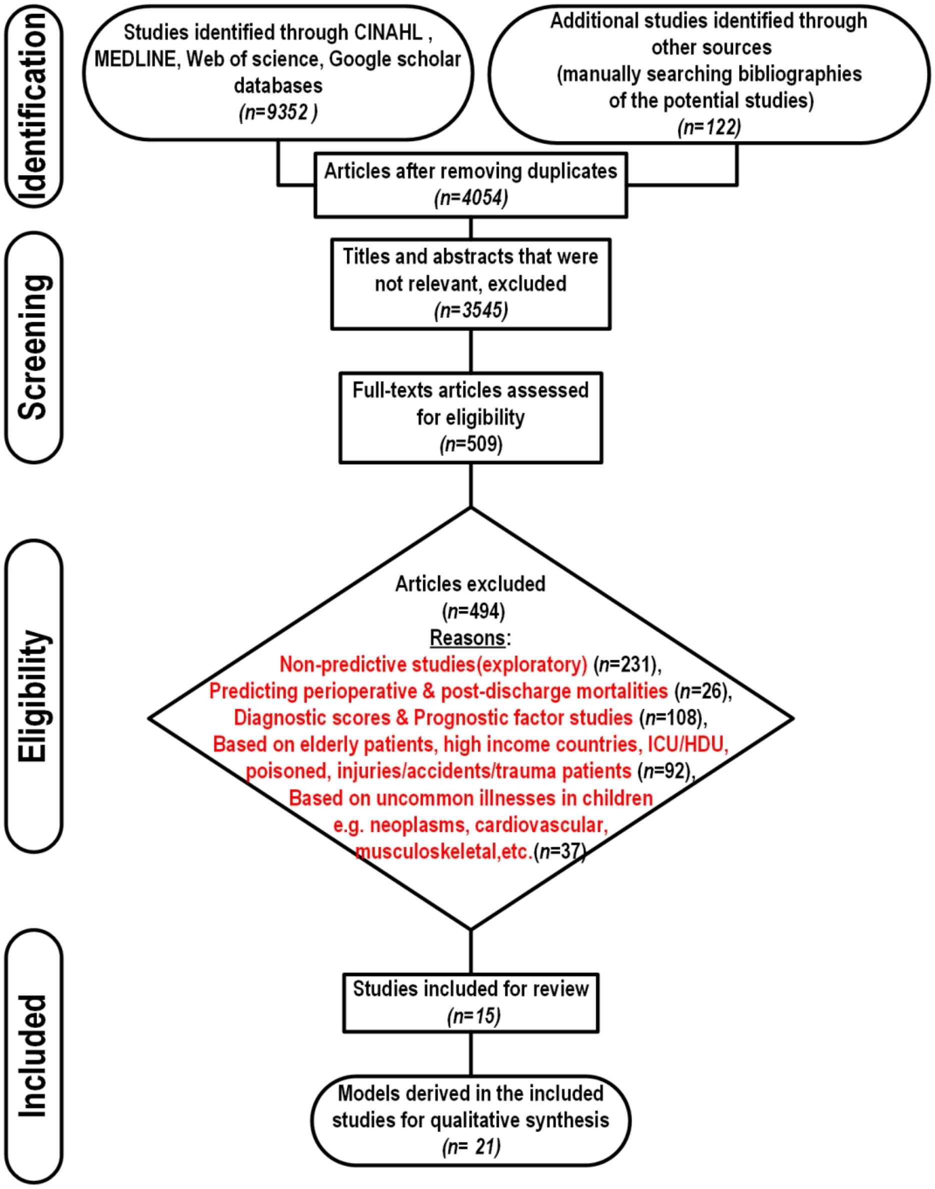

Figure 1: PRISMA flow diagram showing the process used to identify prognostic models predicting in-hospital paediatric mortality included in this review

127x164mm (300 x 300 DPI)

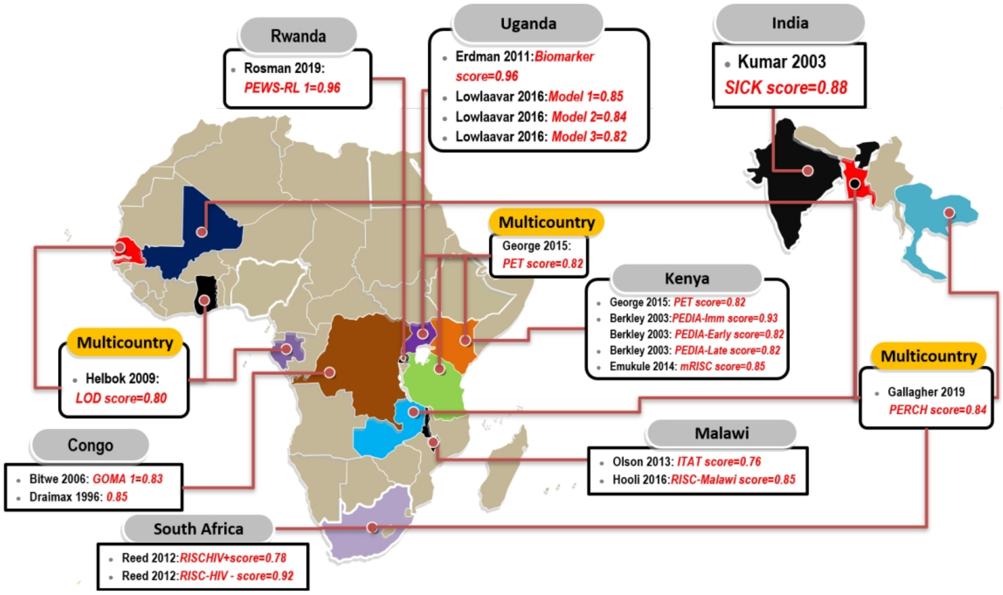

Figure 2: Prognostic models predicting in-hospital paediatric mortality identified by country. Text highlighted in red are the names of the models with their corresponding discrimination measures (area under the curve). Key: PEWS-RL score=Paediatric Early Warning Score for Resource-Limited Settings; SICK score=Signs of Inflammation in Children that Kill; PET score= Paediatric Emergency Triage; mRISC score= Modified Respiratory Index of Severity in Children score; RISC score= Respiratory Index of Severity in Children score; PERCH severity score= Pneumonia Etiology Research for Child Health severity score; LOD score= Lambarene Organ Dysfunction score; CRT= Classification and Regression Trees; ITAT Score= Inpatient Triage Assessment and Treatment score; PEDIA score= Paediatric Early Death Index for Africa score.

137x81mm (300 x 300 DPI)

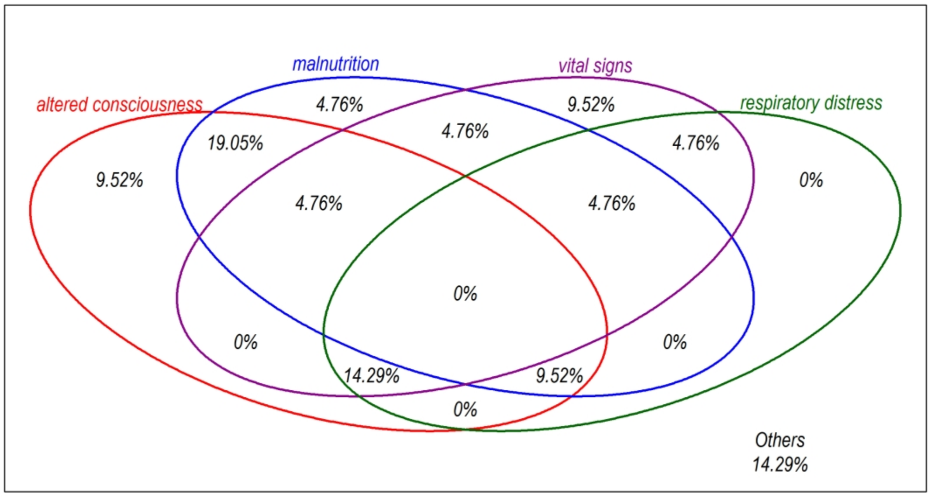

Figure 3: Top four categories of predictors in the models of the reviewed reports: altered consciousness (coma, prostration, not alert, unconscious); malnutrition indicators (kwashiorkor, edema, weight-for-height z-score, weight-for-age z-score, mid-upper arm circumference-MUAC, wasting); vital signs (temperature, respiratory rate, heart rate, oxygen saturation); signs of respiratory distress (indrawing, lung crepitation, difficult breathing, grunting).

228x127mm (300 x 300 DPI)

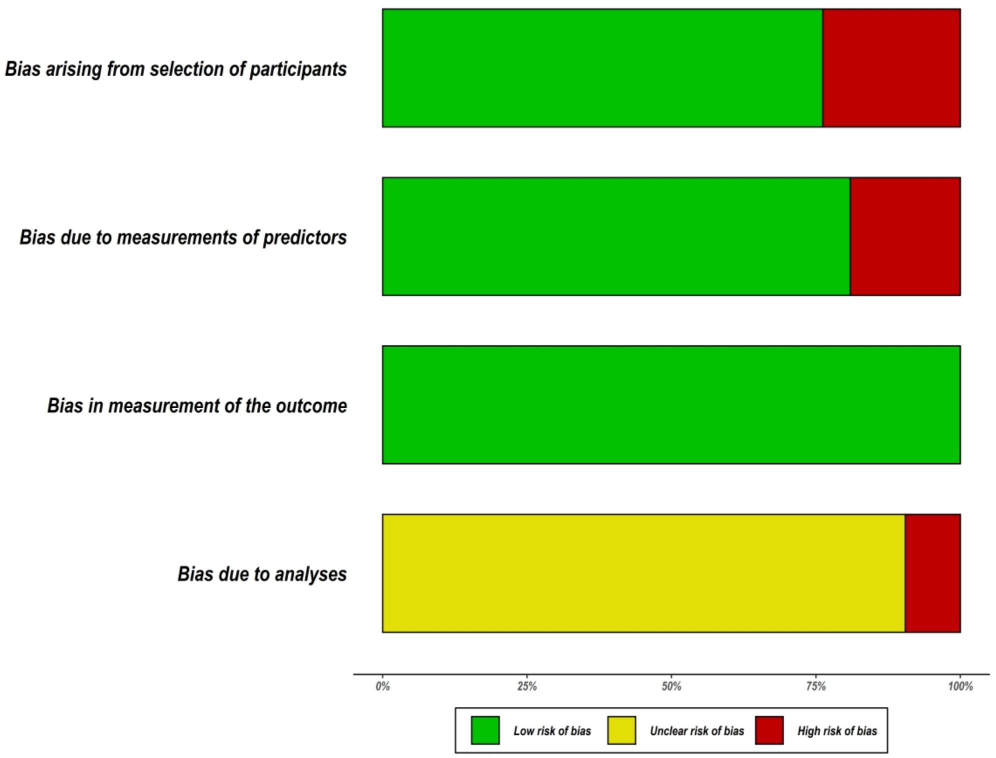

Figure 4: Summary of the risk of bias of the included models using PROBAST (Prediction study Risk of Bias Assessment Tool).

254x203mm (600 x 600 DPI)

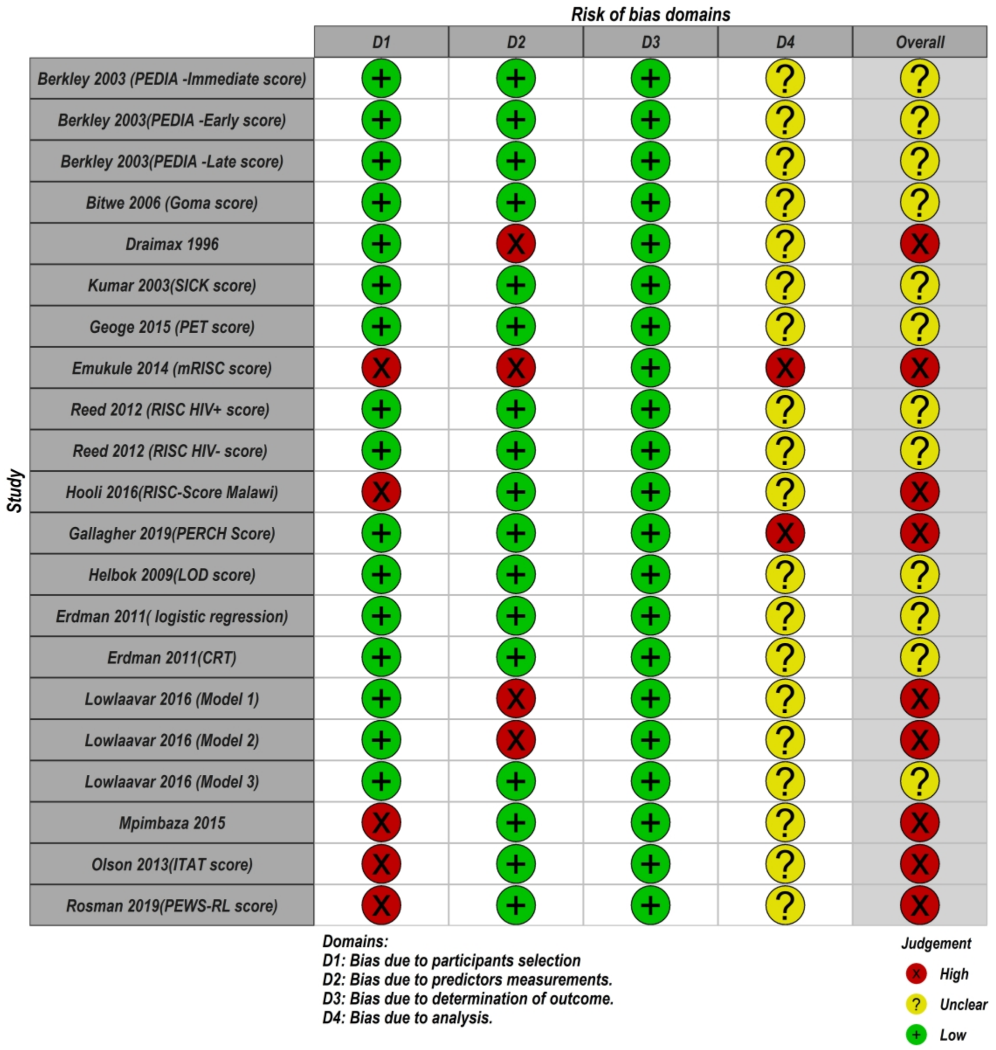

Figure 5: Risk of bias assessment. Low means low risk of bias, High means a high risk of bias, and Unclear bias means it was not possible to assess the risk of bias. Key: PEWS-RL score=Paediatric Early Warning Score for Resource-Limited Settings; SICK score=Signs of Inflammation in Children that Kill; PET score= Paediatric Emergency Triage; mRISC score= Modified Respiratory Index of Severity in Children score; RISC score= Respiratory Index of Severity in Children score; PERCH severity score= Pneumonia Etiology Research for Child Health severity score; LOD score= Lambarene Organ Dysfunction score; CRT= Classification and Regression Trees; ITAT Score= Inpatient Triage Assessment and Treatment score; PEDIA score= Paediatric Early Death Index for Africa score.

199x219mm (600 x 600 DPI)

Table 1: Systematic review framework as recommended by CHARMS checklist

Item	Criteria
Prognostic or diagnostic model	Prognostic model predicting in-hospital mortality.
Scope	Prognostic models to inform clinicians about the risk of deterioration or death.
Type of prediction models	Prognostic models with and/or without external validation.
Prediction target population	Children aged > 1 month to 15 years admitted in pediatric wards in developing countries
Outcome of interest	All-cause in-hospital mortality.
Prediction period	Any
Intended moment to apply the prediction tool	Prognostic model to be used in primary prevention to assess risk of deterioration and thus guide prevention/treatment.

KEY:

CHARMS= Checklist for critical Appraisal and data extraction for systematic Reviews of prediction Modelling Studies

For peer review only

Table 2: Search terms for prognostic models

Search ID	Sub-heading	Search Terms
S4	Children	paediatric* OR pediatric* OR (MH "Pediatrics+") OR child*
S3	Hospital based	(MH "Hospitals+") OR hospital*
S2	Low-income countries	(MH "Developing Countries+") OR (MH "Africa+") OR TI ("low income" OR "low and middle income"OR "LMIC" OR "LIC" OR "limited resource*" OR "poor resource*" OR "resource* poor" OR ("developing countries") OR ("developing nations") OR ("third world") OR "resource-constrained" OR ("global south")
S1	Predictive models	prognos* OR (MH "prognosis") OR (Predict* AND (Outcome* OR Risk* OR Model* OR Mortality OR Index OR Rule* OR decision* OR scor*)) OR "risk score" OR "scor* system" OR "logistic model*" "risk prediction" OR "risk calculation" OR "risk assessment" OR "c statistic" OR discrimination OR calibration OR AUC OR "area under the curve" OR "area under the receiver operator characteristic curve"

For peer review only

Table 3: List of domains and signaling questions used for assessment of risk of bias according to the PROBAST tool.

Domain	Signalling question
**Participants selection**	Were appropriate data sources used, e.g., cohort, RCT, or nested case–control study data?
	Were all inclusions and exclusions of participants appropriate?
**Predictors**	Were predictors defined and assessed in a similar way for all participants?
	Were predictor assessments made without knowledge of outcome data?
	Are all predictors available at the time the model is intended to be used?
**Outcome**	Was the outcome determined appropriately?
	Was a prespecified or standard outcome definition used?
	Were predictors excluded from the outcome definition?
	Was the outcome defined and determined in a similar way for all participants?
	Was the outcome determined without knowledge of predictor information?
	Was the time interval between predictor assessment and outcome determination appropriate?
**Analysis**	Were there a reasonable number of participants with the outcome?
	Were continuous and categorical predictors handled appropriately?
	Were all enrolled participants included in the analysis?
	Were participants with missing data handled appropriately?
	Was selection of predictors based on univariable analysis avoided?
	Were complexities in the data (e.g., censoring, competing risks, sampling of control participants) accounted for appropriately?
	Were relevant model performance measures evaluated appropriately?
	Were model overfitting, underfitting, and optimism in model performance accounted for?
	Do predictors and their assigned weights in the final model correspond to the results from the reported multivariable analysis?

KEY:

PROBAST= Prediction study Risk of Bias Assessment Tool

For peer review only

Study	*Model name*	*Country*	*Source of data*	*Study year*	*Inclusion criteria*	*Age*	*Outcome*
Berkley 2003	PEDIA Immediate death	Kenya	Prospective cohort	1998-2000	Aged over 90 days	3 months-13 years	Mortality
Berkley 2003	PEDIA Early death	Kenya	Prospective cohort	1998-2000	Aged over 90 days	3 months-13 years	Mortality
Berkley 2003	PEDIA Late death	Kenya	Prospective cohort	1998-2000	Aged over 90 days	3 months-13 years	Mortality
Bitwe 2006	Goma 1 Model	Democratic Republic of Congo	Prospective cohort	2003-2004	<12 months	Median: 12.8 months	Mortality
Draimax 1996		Congo	Prospective cohort	1986-1988	Malnutrition	Median: 27 months	Mortality
Kumar 2003	SICK score	India	Prospective cohort	1998-1999	Paediatric patients	No Information	Mortality
Geoge 2015	PET Score	Kenya, Uganda, Tanzania	RCT	2009-2011	Malaria	Median: 24 (IQR=13-38)	Mortality
Emukule 2014	mRISC	Kenya	Surveillance	2009-2012	Under 5 years hospitalized with severe acute respiratory illness	<59 months	Mortality
Reed 2012	RISC-HIV positive	South Africa	RCT	1998-2001	LRTI hospitalizations under 24 months with HIV infection	<24 months	Mortality
Reed 2012	RISC-HIV Negative	South Africa	RCT	1998-2001	LRTI hospitalizations under 24 months without HIV infection	<24 months	Mortality
Hooli 2016	RISC-Malawi	Malawi	Retrospective observational study	2011-2014	0-59 months hospitalized with pneumonia	<59 months	Mortality
Gallagher 2019	PERCH Score	Kenya, Zambia, South Africa, Mali, Gambia, Bangladesh, Thailand	Case-control study	2011–2014	1–59 months HIV negative hospitalized with severe or very severe pneumonia	Median: 9(4-19) months	In-hospital mortality and 7-days post-

							discharge mortality
Helbok 2009	LOD score	Gambia,Malawi, Kenya,Ghana,Gabon	Prospective cohort	2000-2005	Hospitalized children with severe malaria	28(0-180)	Mortality
Erdman 2011 (Logistic regression)	Biomarker score	Uganda	Retrospective nested case-control study	2007-2009	6 months - 12 years	6 months - 12 years	Mortality
Erdman 2011 (Classification tree)		Uganda	Retrospective nested case-control study	2007-2009	6 months - 12 years	6 months - 12 years	Mortality
Lowlaavar 2016	Model 1	Uganda	Prospective observational study	2012-2013	6–60 months admitted with infectious illness	Median 18.2 (IQR 11.9–33.1) months	Mortality
Lowlaavar 2016	Model 2	Uganda	Prospective observational study	2012-2013	6–60 months admitted with infectious illness	Median 18.2 (IQR 11.9–33.1) months	Mortality
Lowlaavar 2016	Model 3	Uganda	Prospective observational study	2012-2013	6–60 months admitted with infectious illness	Median 18.2 (IQR 11.9–33.1) months	Mortality
Mpimbaza 2015		Uganda	Surveillance	2010-2013	General paediatrics	18 months (IQR 9–36)	Mortality
Olson 2013	ITAT score	Malawi	Nested case–control	2010-2011	age <15 years on the acute care and malnutrition wards	≤15 years	Mortality
Rosman 2019	PEWS-RL	Rwanda	Case-control study	2016-2017	0-18 years patients admitted to pediatric department	0-18 years	Mortality

Study	***Sample size***	***Number of outcome events***	***Missing data handling***	***Number of participants with missing data reported?***	***Regression method***	***Were model assumptions verified***
Berkley 2003	429	60	No Information	No Information	Spiegelhalter/Knill-Jones method	Yes

For peer review only

Berkley 2003	439	193	No Information	No Information	Spiegelhalter/Knill-Jones method	Yes
Berkley 2003	436	183	No Information	No Information	Spiegelhalter/Knill-Jones method	Yes
Bitwe 2006	414	66	No Information	No Information	Logistic regression	Yes
Draimax 1996	1129	196	No Information	No Information	Logistic regression	No Information
Kumar 2003	1099	44	No Information	No Information	Logistic regression	No Information
Geoge 2015	3170	315	Complete case analyses	Yes	Cox proportional hazards regression	No Information
Emukule 2014	3581	218	Complete case analyses	No Information	Logistic regression	Yes
Reed 2012	1502	265	Complete case analyses	No Information	Logistic regression	No Information
Reed 2012	2646	33	Complete case analyses	No Information	Logistic regression	No Information
Hooli 2016	14665	464	Multiple imputation	Yes	Logistic regression	Yes
Gallagher 2019	1802	120	Complete case analyses	No Information	Logistic regression	No Information
Helbok 2009	23980	1004	Complete case analyses	Yes	Logistic regression	No Information
Erdman 2011 (Logistic regression)	103	23	No missing values	Yes	Logistic regression	Yes
Erdman 2011 (Classification tree)	103	23	No missing values	Yes	Classification tree	No Information
Lowlaavar 2016	1307	65	Multiple imputation	No Information	Logistic regression	No Information
Lowlaavar 2016	1307	65	Multiple imputation	No Information	Logistic regression	No Information
Lowlaavar 2016	1307	65	Multiple imputation	No Information	Logistic regression	No Information

For peer review only

Mpimbaza 2015	50249	1742	Complete case analyses	Yes	Logistic regression	No Information
Olson 2013	1606	54	Single imputation	Yes	Logistic regression	Yes
Rosman 2019	168	57	Complete case analyses	No Information	Logistic regression	No Information

Study	***Predictor selection***	***Was a shrinkage method used***	***Calibration method***	***Discrimination***
Berkley 2003	Univariate	No Information	No Information	0.93(0.92-0.94)
Berkley 2003	Univariate	No Information	No Information	0.82(0.80-0.83)
Berkley 2003	Univariate	No Information	No Information	0.82(0.81-0.84)
Bitwe 2006	Univariate & Stepwise	No Information	Yes	0.83 (0.78-0.88)
Draimax 1996	A priori	No Information	No Information	0.85(No information)
Kumar 2003	Univariate(but included all variables in final model)	No Information	No Information	0.89
Geoge 2015	A priori	No Information	Hosmer-Lemeshow test, P=0.30	0.82(0.77–0.87)
Emukule 2014	Univariate	Yes	Calibration plot	0.85
Reed 2012	Univariate	No Information	Hosmer-Lemeshow test, P=0.95	0.78
Reed 2012	Univariate	No Information	Hosmer-Lemeshow test, P=0.87	0.92
Hooli 2016	A priori	No Information	Risk predictiveness curve	0.79 (95% CI: 0.76±0.82)
Gallagher 2019	Univariate	No Information	Calibration plot	0.84(No Information)
Helbok 2009	Forward & backward Stepwise	No Information	No Information	80 (79–82)

For peer review only

Erdman 2011 (Logistic regression)	Univariate	No Information	Hosmer-Lemeshow test and calibration slope analysis	0.96(0.90–0.99)
Erdman 2011 (Classification tree)	No Information	No Information	No Information	No Information
Lowlaavar 2016	Univariate & Stepwise	No Information	No Information	0.85 (0.80–0.89)
Lowlaavar 2016	Univariate & Stepwise	No Information	No Information	0.84 (0.79–0.89)
Lowlaavar 2016	Univariate & Stepwise	No Information	No Information	0.82 (0.72–0.91)
Mpimbaza 2015	Backward	No Information	General paediatrics	0.76(No information)
Olson 2013	Univariate	No Information	No Information	0.76(No information)
Rosman 2019	Univariate	No Information	No Information	0.96 (95% CI 0.93–0.99).

Study	***Classification measures reported***	***Method used for internal validation***	***External validation***	***Was a simplified model presented***	***Were coefficients (including intercept) of the regression model presented***
Berkley 2003	No Information	Separate dataset	Yes	Yes	NA
Berkley 2003	No Information	Separate dataset	Yes	Yes	NA
Berkley 2003	No Information	Separate dataset	Yes	Yes	NA
Bitwe 2006	No Information	No Information	No	Yes	No
Draimax 1996	Positive predictive values 40% and negative predictive value of 97.9%	Separate dataset	No	Yes	No
Kumar 2003	Maximum discrimination was observed at a score of 2.5 with a sensitivity of 84.1% and of specificity 82.2%	No Information	Yes	Yes	No
Geoge 2015	No Information	Separate dataset	No	Yes	No
Emukule 2014	A score of >6 has a sensitivity of 1.8% and specificity 99.9%	Bootstrapping	Yes	Yes	No
Reed 2012	Score of 7 has a sensitivity of 4% and specificity of 99%	Bootstrapping	No	Yes	No

Reed 2012	Score of 6 has a sensitivity: 16% Specificity: 99%	Bootstrapping	Yes	Yes	No
Hooli 2016	a score of 8 has sensitivity of 57% and specificity of 88%	No Information	No	Yes	Yes
Gallagher 2019	positive predictive value 23.6%, positive predictive value 95.8%	Bootstrapping & separate dataset	No	Yes	No
Helbok 2009	LODS >=1, sensitivity was 85% and specificity was 63%	No Information	Yes	Yes	No
Erdman 2011 (Logistic regression)	sensitivity of 95.7% (95% CI: 78.1–99.9) and specificity of 88.8% (79.7–94.7) predicting death	Boostrappling	No	Yes	No
Erdman 2011 (Classification tree)	100% sensitivity and 92.5% specificity for predicting outcome	10-fold cross validation	No	No	No
Lowlaavar 2016	Sensitive: 0.83 (0.74–0.92), Specificity: 0.76 (0.73–0.78)	No Information	No	No	Yes
Lowlaavar 2016	Sensitive: 0.80 (0.70–0.90), Specificity: 0.76 (0.74–0.79)	No Information	No	No	Yes
Lowlaavar 2016	Sensitive: 0.82 (0.72–0.91), Specificity: 0.71 (0.68–0.73)	No Information	No	No	Yes
Mpimbaza 2015	No Information	Separate dataset	No	Yes	No
Olson 2013	sensitivity: 0.44, specificity: 0.86,PPV: 0.18, NPV: 0.96 for a cut-off of 4	No Information	No	Yes	No
Rosman 2019	PEWS-RL of >=3, sensitivity was 96.2%, and specificity was 87.3%	No Information	No	Yes	No

Study	***Number of predictors in final model***	***Predictors in the final model***	***Are there laboratory-based predictors***	***Handling of continuous predictors***	***Events per variable***
Berkley 2003	10	Severe anaemia, Jaundice, Subcostal indrawing, Deep breathing, prostrated with seizures, prostrated without	No	NA	6

For peer review only

		seizures, Impaired consciousness with seizures, Impaired consciousness without seizures, Axillary temperature <36 °C, Axillary temperature >39 °C			
Berkley 2003	8	Jaundice, Subcostal indrawing, Prostrated with seizures, Prostrated without seizures, Impaired consciousness with seizures, Impaired consciousness without seizures,Wasting, Kwashiorkor	No	NA	24.125
Berkley 2003	9	History >7 days, Prostrated with seizures, Prostrated without seizures, Impaired consciousness with seizures, Impaired consciousness without seizures, Axillary temperature <36 °C, Axillary temperature >39 °C, Wasting, Kwashiorkor	No	Dichotomized History	20.33333
Bitwe 2006	8	Age(<12, >=12months), Brachial Perimeter(<=115mm, >115mm), State of consciousness(Unconscious, Aware), Infectious diagnosis(Acute respiratory infection, Malaria, Gastroenteritis, Septicemia / bacteremia, Other infections)	No	Dichotomized Brachial perimeter & Age	8.25

For peer review only

Draimax 1996	4	MUAC, edema, Serum albumin, Transthyretin	Yes	MUAC	49
Kumar 2003	9	Temperature(Normal, Abnormal), Heart rate(Normal, Abnormal), Respiratory rate(Normal, Abnormal), Systolic blood pressure(Normal, Abnormal), Capillary refill time(Normal, Abnormal), Consciousness(Normal, Abnormal), Age(≥60, ≥12 to <60, ≥1to<12, <1)	No	Dichotomized most variables	4.888889
Geoge 2015	11	Temperature(≤37, >37), Heart rate(<80 bpm, ≥80 to <105 bpm, ≥220 bpm), Capillary refill time(≥2sec, <2sec), Conscious level(prostrate, coma), Respiratory distress, Lung crepitations, Severe pallor, Weak pulse, Weight(<6 kg, 6–8 kg), Deep breathing	No	multivariable fractional polynomials	28.63636
Emukule 2014	9	Lab confirmed malaria, Weight for age(Low, Very Low), Dehydration, Unconscious, Unable to drink/breastfeed, Night sweats, Chest wall in-drawing, Interaction between malaria and chest wall in-drawing, A.V.P.U scale - Not alert	Yes	Categorized weight for age	24.22222

Reed 2012	7	Oxygen saturation <90%, Chest indrawing, Wheezing, Refusing feeds, HIV classification(Severe, Mild or moderate), IMCI age group(<2 months, 3–12 months)	No		37.85714
Reed 2012	5	Oxygen saturation <90%, Chest indrawing, Wheezing, Refusing feeds, Weight for age(Low (<= -2 z-score), Very Low (<= -3 z-score))	No	Categorized weight for age	6.6
Hooli 2016	5	Oxygen saturation(moderate, severe), MUAC(moderate, severe), Gender, Wheeze, Consciousness	No	Categorized MUAC and Oxygen saturation	92.8
Gallagher 2019	12	Age(1-11, 12-59), sex, Unresponsiveness and/or deep breathing(Deep breathing, but alert, Unresponsive but no deep breathing, Unresponsive and deep breathing), cough, grunting, hypoxemia, length of illness(0–2, 3–5, >5), Weight-for-height z-score(Very low (< –3), Low (≥ –3 to < –2), Normal-high (≥ –2))	No	Categorized most variables	10
Helbok 2009	8	Convulsion, vomiting, deep breathing, intercostal recession, Coma,	Yes	NA	125.5

For peer review only

		Prostration,hyperparastemia, severe anemia			
Erdman 2011 (Logistic regression)	8	angiopoietin-2, soluble ICAM-1, soluble Flt-1, procalcitonin, IP-10, soluble TREM-1, age, parasitemia	Yes	NA	2.875
Erdman 2011 (Classification tree)	3	IP-10, Ang-2, sICAM-1	Yes	NA	7.666667
Lowlaavar 2016	3	Abnormal BCS, Positive HIV diagnosis, Weight-age z-score	Yes	Treated as continuous	21.66667
Lowlaavar 2016	3	Abnormal BCS, HIV diagnosis, MUAC	Yes	Treated as continuous	21.66667
Lowlaavar 2016	2	Abnormal BCS, MUAC	No	Treated as continuous	32.5
Mpimbaza 2015	13	Age, fever, difficulty breathing, altered consciousness, unable to drink or breastfeed, convulsions, temperature, unconsciousness, pallor, jaundice, deep breathing, meningeal signs, unable to sit up	No	NA	134
Olson 2013	4	Oxygen saturation, Temperature, Heart rate, Respiratory rate	No	Used splines	13.5
Rosman 2019	6	PEWS-RL score(0 to 6)	No	NA	9.5

For peer review only

Study	Participant Domain		
	Were appropriate data sources used, e.g., cohort, RCT, or nested case–control study data?	*Were all inclusions and exclusions of participants appropriate?*	*Risk of Bias in participants*
Berkley 2003 (PEDIA -Immediate)	Yes	Yes	Low
Berkley 2003(PEDIA -Early)	Yes	Yes	Low
Berkley 2003(PEDIA -Late)	Yes	Yes	Low
Bitwe 2006 (Goma score)	Yes	Yes	Low
Draimax 1996	Yes	Yes	Low
Kumar 2003(SICK score)	Yes	Yes	Low
Geoge 2015 (PET score)	Yes	Yes	Low
Emukule 2014 (mRISC score)	No	Yes	High
Reed 2012 (RISC HIV+)	Yes	Yes	Low
Reed 2012 (RISC HIV-)	Yes	Yes	Low
Hooli 2016(RISC-Malawi)	No	Yes	High
Gallagher 2019(PERCH Score)	Yes	Yes	Low
Helbok 2009(LOD score)	Yes	Yes	Low
Erdman 2011(logistic regression)	Yes	Yes	Low
Erdman 2011(CRT)	Yes	Yes	Low
Lowlaavar 2016 (Model 1)	Yes	Yes	Low
Lowlaavar 2016 (Model 2)	Yes	Yes	Low
Lowlaavar 2016 (Model 3)	Yes	Yes	Low
Mpimbaza 2015	No	Yes	High
Olson 2013(ITAT score)	Yes	Yes	Low
Rosman 2019(PEWS-RL score)	No	Yes	High

For peer review only

Study	Predictor Domain			
	Were predictors defined and assessed in a similar way for all participants	*Were predictor assessments made without knowledge of outcome data?*	*Are all predictors available at the time the model is intended to be used?*	*Risk of Bias in predictors*
Berkley 2003 (PEDIA -Immediate)	Yes	Yes	Yes	Low
Berkley 2003(PEDIA -Early)	Yes	Yes	Yes	Low
Berkley 2003(PEDIA -Late)	Yes	Yes	Yes	Low
Bitwe 2006 (Goma score)	Yes	Yes	Yes	Low
Draimax 1996	Yes	Yes	No	High
Kumar 2003(SICK score)	Yes	Yes	Yes	Low
Geoge 2015 (PET score)	Yes	Yes	Yes	Low
Emukule 2014 (mRISC score)	Yes	Yes	No	High
Reed 2012 (RISC HIV+)	Yes	Yes	Yes	Low
Reed 2012 (RISC HIV-)	Yes	Yes	Yes	Low
Hooli 2016(RISC-Malawi)	Yes	Yes	Yes	Low
Gallagher 2019(PERCH Score)	Yes	Yes	Yes	Low
Helbok 2009(LOD score)	Yes	Yes	Yes	Low
Erdman 2011(logistic regression)	Yes	Yes	Yes	Low
Erdman 2011(CRT)	Yes	Yes	Yes	Low
Lowlaavar 2016 (Model 1)	Yes	Yes	No	High
Lowlaavar 2016 (Model 2)	Yes	Yes	No	High
Lowlaavar 2016 (Model 3)	Yes	Yes	Yes	Low
Mpimbaza 2015	Yes	Yes	Yes	Low
Olson 2013(ITAT score)	Yes	Yes	Yes	Low
Rosman 2019(PEWS-RL score)	Yes	Yes	Yes	Low

For peer review only

Study	Outcome Domain						
	Was the outcome determined appropriately?	*Was a prespecified or standard outcome definition used?*	*Were predictors excluded from the outcome definition?*	*Was the outcome defined and determined in a similar way for all participants?*	*Was the outcome determined without knowledge of predictor information?*	*Was the time interval between predictor assessment and outcome determination appropriate?*	*Risk of Bias in outcome*
Berkley 2003 (PEDIA -Immediate)	Yes	Yes	Yes	Yes	Yes	Yes	Low
Berkley 2003(PEDIA -Early)	Yes	Yes	Yes	Yes	Yes	Yes	Low
Berkley 2003(PEDIA -Late)	Yes	Yes	Yes	Yes	Yes	Yes	Low
Bitwe 2006 (Goma score)	Yes	Yes	Yes	Yes	Yes	Yes	Low
Draimax 1996	Yes	Yes	Yes	Yes	Yes	Yes	Low
Kumar 2003(SICK score)	Yes	Yes	Yes	Yes	Yes	Yes	Low
Geoge 2015 (PET score)	Yes	Yes	Yes	Yes	Yes	Yes	Low
Emukule 2014 (mRISC score)	Yes	Yes	Yes	Yes	Yes	Yes	Low
Reed 2012 (RISC HIV+)	Yes	Yes	Yes	Yes	Yes	Yes	Low
Reed 2012 (RISC HIV-)	Yes	Yes	Yes	Yes	Yes	Yes	Low
Hooli 2016(RISC-Malawi)	Yes	Yes	Yes	Yes	Yes	Yes	Low
Gallagher 2019(PERCH Score)	Yes	Yes	Yes	Yes	Yes	Yes	Low
Helbok 2009(LOD score)	Yes	Yes	Yes	Yes	Yes	Yes	Low
Erdman 2011(logistic regression)	Yes	Yes	Yes	Yes	Yes	Yes	Low
Erdman 2011(CRT)	Yes	Yes	Yes	Yes	Yes	Yes	Low
Lowlaavar 2016 (Model 1)	Yes	Yes	Yes	Yes	Yes	Yes	Low
Lowlaavar 2016 (Model 2)	Yes	Yes	Yes	Yes	Yes	Yes	Low
Lowlaavar 2016 (Model 3)	Yes	Yes	Yes	Yes	Yes	Yes	Low
Mpimbaza 2015	Yes	Yes	Yes	Yes	Yes	Yes	Low
Olson 2013(ITAT score)	Yes	Yes	Yes	Yes	Yes	Yes	Low
Rosman 2019(PEWS-RL score)	Yes	Yes	Yes	Yes	Yes	Yes	Low

For peer review only

Study	Analyses Domain				
	Were there a reasonable number of participants with the outcome?	*Were continuous and categorical predictors handled appropriately?*	*Were all enrolled participants included in the analysis?*	*Were participants with missing data handled appropriately?*	*Was selection of predictors based on univariable analysis avoided*
Berkley 2003 (PEDIA -Immediate)	No	Yes	Yes	Probably No	No
Berkley 2003(PEDIA -Early)	Yes	Yes	Yes	Probably No	No
Berkley 2003(PEDIA -Late)	Yes	No	Yes	Probably No	No
Bitwe 2006 (Goma score)	No	No	Yes	No Information	No
Draimax 1996	Yes	No	Yes	No Information	Yes
Kumar 2003(SICK score)	No	No	Yes	No Information	No
Geoge 2015 (PET score)	Yes	Yes	Yes	No	Yes
Emukule 2014 (mRISC score)	Yes	No	Yes	No	No
Reed 2012 (RISC HIV+)	Yes	Yes	Yes	No Information	No
Reed 2012 (RISC HIV-)	No	No	Yes	No Information	No
Hooli 2016(RISC-Malawi)	Yes	No	Yes	Yes	Yes
Gallagher 2019(PERCH Score)	Yes	No	Yes	No	No
Helbok 2009(LOD score)	Yes	Yes	Yes	No	No
Erdman 2011(logistic regression)	No	Yes	Yes	Yes	No
Erdman 2011(CRT)	No	Yes	Yes	Yes	No Information
Lowlaavar 2016 (Model 1)	Yes	Yes	Yes	Yes	No
Lowlaavar 2016 (Model 2)	Yes	Yes	Yes	Yes	No
Lowlaavar 2016 (Model 3)	Yes	Yes	Yes	Yes	No
Mpimbaza 2015	Yes	Yes	Yes	No	No
Olson 2013(ITAT score)	Yes	Yes	Yes	Probably No	No
Rosman 2019(PEWS-RL score)	No	Yes	Yes	No	No

For peer review only

Study	Analyses Domain				
	Were complexities in the data (e.g., censoring, competing risks, sampling of control participants) accounted for appropriately?	*Were relevant model performance measures evaluated appropriately?*	*Were model overfitting, underfitting, and optimism in model performance accounted for?*	*Do predictors and their assigned weights in the final model correspond to the results from the reported multivariable analysis?*	*Risk of Bias in analysis*
Berkley 2003 (PEDIA -Immediate)	NA	Probably No	No Information	Probably Yes	Unclear
Berkley 2003(PEDIA -Early)	NA	Probably No	No Information	Probably Yes	Unclear
Berkley 2003(PEDIA -Late)	NA	Probably No	No Information	Probably Yes	Unclear
Bitwe 2006 (Goma score)	NA	Yes	No Information	Yes	Unclear
Draimax 1996	NA	No	No Information	Probably No	Unclear
Kumar 2003(SICK score)	NA	No	No Information	Yes	Unclear
Geoge 2015 (PET score)	No	No	No Information	Yes	Unclear
Emukule 2014 (mRISC score)	NA	Yes	Yes	Yes	High
Reed 2012 (RISC HIV+)	NA	Yes	Yes	Yes	Unclear
Reed 2012 (RISC HIV-)	NA	Yes	Yes	Yes	Unclear
Hooli 2016(RISC-Malawi)	NA	Yes	No Information	Yes	Unclear
Gallagher 2019(PERCH Score)	NA	Yes	Yes	Yes	High
Helbok 2009(LOD score)	NA	Probably No	No Information	Probably No	Unclear
Erdman 2011(logistic regression)	NA	No	Yes	No Information	Unclear
Erdman 2011(CRT)	NA	No Information	Yes	No Information	Unclear
Lowlaavar 2016 (Model 1)	NA	No	No Information	Yes	Unclear
Lowlaavar 2016 (Model 2)	NA	No	No Information	Yes	Unclear
Lowlaavar 2016 (Model 3)	NA	No	No Information	Yes	Unclear
Mpimbaza 2015	NA	No Information	No Information	Yes	Unclear
Olson 2013(ITAT score)	NA	No	No Information	No	Unclear
Rosman 2019(PEWS-RL score)	NA	No	No Information	No	Unclear

For peer review only

For peer review only

Reporting checklist for systematic review and meta-analysis.

Based on the PRISMA guidelines.

Instructions to authors

Complete this checklist by entering the page numbers from your manuscript where readers will find each of the items listed below.

Your article may not currently address all the items on the checklist. Please modify your text to include the missing information. If you are certain that an item does not apply, please write "n/a" and provide a short explanation.

Upload your completed checklist as an extra file when you submit to a journal.

In your methods section, say that you used the PRISMAreporting guidelines, and cite them as:

Moher D, Liberati A, Tetzlaff J, Altman DG, The PRISMA Group. Preferred Reporting Items for Systematic Reviews and Meta-Analyses: The PRISMA Statement

For peer review only

		Reporting Item	Page Number
**Title**			
	#1	Identify the report as a systematic review, meta-analysis, or both.	1
**Abstract**			

Structured summary	#2	Provide a structured summary including, as applicable: background; objectives; data sources; study eligibility criteria, participants, and interventions; study appraisal and synthesis methods; results; limitations; conclusions and implications of key findings; systematic review registration number	2
**Introduction**			
Rationale	#3	Describe the rationale for the review in the context of what is already known.	3
Objectives	#4	Provide an explicit statement of questions being addressed with reference to participants, interventions, comparisons, outcomes, and study design (PICOS).	3
**Methods**			
Protocol and registration	#5	Indicate if a review protocol exists, if and where it can be accessed (e.g., Web address) and, if available, provide registration information including the registration number.	4
Eligibility criteria	#6	Specify study characteristics (e.g., PICOS, length of follow-up) and report characteristics (e.g., years considered, language, publication status) used as criteria for eligibility, giving rational	4
Information sources	#7	Describe all information sources in the search (e.g., databases with dates of coverage, contact with study authors to identify additional studies) and date last searched.	N/A
Search	#8	Present full electronic search strategy for at least one database, including any limits used, such that it could be repeated.	5

For peer review only

Study selection	#9	State the process for selecting studies (i.e., for screening, for determining eligibility, for inclusion in the systematic review, and, if applicable, for inclusion in the meta-analysis).	6
Data collection process	#10	Describe the method of data extraction from reports (e.g., piloted forms, independently by two reviewers) and any processes for obtaining and confirming data from investigators.	6
Data items	#11	List and define all variables for which data were sought (e.g., PICOS, funding sources), and any assumptions and simplifications made.	6
Risk of bias in individual studies	#12	Describe methods used for assessing risk of bias in individual studies (including specification of whether this was done at the study or outcome level, or both), and how this information is to be used in any data synthesis.	7
Summary measures	#13	State the principal summary measures (e.g., risk ratio, difference in means).	N/A
Planned methods of analyis	#14	Describe the methods of handling data and combining results of studies, if done, including measures of consistency (e.g., I2) for each meta-analysis.	N/A
Risk of bias across studies	#15	Specify any assessment of risk of bias that may affect the cumulative evidence (e.g., publication bias, selective reporting within studies).	7
Additional analyses	#16	Describe methods of additional analyses (e.g., sensitivity or subgroup analyses, meta-regression), if done, indicating which	N/A

For peer review only

		were pre-specified.	
**Results**			
Study selection	#17	Give numbers of studies screened, assessed for eligibility, and included in the review, with reasons for exclusions at each stage, ideally with a flow diagram.	8
Study characteristics	#18	For each study, present characteristics for which data were extracted (e.g., study size, PICOS, follow-up period) and provide the citation.	8
Risk of bias within studies	#19	Present data on risk of bias of each study and, if available, any outcome-level assessment (see Item 12).	11
Results of individual studies	#20	For all outcomes considered (benefits and harms), present, for each study: (a) simple summary data for each intervention group and (b) effect estimates and confidence intervals, ideally with a forest plot.	N/A
Synthesis of results	#21	Present the main results of the review. If meta-analyses are done, include for each, confidence intervals and measures of consistency.	N/A
Risk of bias across studies	#22	Present results of any assessment of risk of bias across studies (see Item 15).	11
Additional analysis	#23	Give results of additional analyses, if done (e.g., sensitivity or subgroup analyses, meta-regression [see Item 16]).	N/A
**Discussion**			

For peer review only

Summary of Evidence	#24	Summarize the main findings, including the strength of evidence for each main outcome; consider their relevance to key groups (e.g., health care providers, users, and policy makers	12
Limitations	#25	Discuss limitations at study and outcome level (e.g., risk of bias), and at review level (e.g., incomplete retrieval of identified research, reporting bias).	15
Conclusions	#26	Provide a general interpretation of the results in the context of other evidence, and implications for future research.	16

Funding

Funding	#27	Describe sources of funding or other support (e.g., supply of data) for the systematic review; role of funders for the systematic review.	17

The PRISMA checklist is distributed under the terms of the Creative Commons Attribution License CC-BY. This checklist was completed on 15. October 2019 using https://www.goodreports.org/, a tool made by the EQUATOR Network in collaboration with Penelope.ai

For peer review only
